# Fermentation of *Abelmoschus manihot* Extract with Halophilic *Bacillus licheniformis* CP6 Results in Enhanced Anti-Inflammatory Activities

**DOI:** 10.3390/nu15020309

**Published:** 2023-01-07

**Authors:** Mi Hwa Park, Yu Jeong Yeom, Dariimaa Ganbat, Min Kyeong Kim, Seong-Bo Kim, Yong-Jik Lee, Sang-Jae Lee

**Affiliations:** 1 Department of Food and Nutrition, College of Medical and Life Science, Silla University, Busan 46958, Republic of Korea; 2 Department of Food Biotechnology, Research Center for Extremophiles & Marine Microbiology, Silla University, Busan 46958, Republic of Korea; 3 Bio-Living Engineering Major, Global Leaders College, Yonsei University, Seoul 03722, Republic of Korea; 4 Department of Bio-Cosmetics, Seowon University, Chungju 28674, Republic of Korea

**Keywords:** *Abelmoschus manihot*, *Bacillus licheniformis* CP6, fermentation, NF-κB, MAPK, anti-inflammation, antioxidation

## Abstract

Microbial fermentation provides a valorization strategy, through biotransformation, to convert plant-derived raw materials into health-promoting agents. In this study, we have investigated the antioxidative activity of *Abelmoschus manihot* fermented with various *Bacillaceae* strains from specific environments and demonstrated the anti-inflammatory effects of *Bacillus licheniformis* CP6 fermented *A. manihot* extract (FAME) in lipopolysaccharide (LPS)-stimulated Raw264.7 macrophages. Of 1500 bacteria isolated from various specific environments, 47 extracellular protease- and amylase-producing strains with qualified presumption safety status, belonging to the family *Bacillaceae*, were selected for *A. manihot* fermentation. Among them, strain CP6, a halophilic bacterium isolated from Tongyeong seawater in Korea and identified as *B. licheniformis*, showed the highest antioxidant activity. In particular, FAME exerted anti-inflammatory effects on LPS-stimulated Raw264.7 macrophages. Consequently, FAME had a potent inhibitory effect on nitric oxide (NO) production in LPS-stimulated macrophages, without cytotoxicity. Moreover, FAME downregulated LPS-induced pro-inflammatory mediator and enzyme levels in LPS-induced Raw264.7 cells, including IL-1β, IL-6, TNF-α, iNOS, and COX-2, compared to levels when cells were incubated in *A. manihot* extract (IAME). Further detailed characterization indicated that FAME suppresses inflammation by blocking NF-κB via IKK phosphorylation inhibition and IκB-α degradation and by downregulating NO production, and inflammatory mediators also decreased NF-κB translocation. Furthermore, FAME inhibited LPS-stimulated activation of MAPKs, including ERK1/2, JNK, and p38, compared to that with either IAME. Therefore, we suggest that FAME could be used for inflammation-related disorders.

## 1. Introduction

In biological systems, inflammation is the main physiological defense mechanism that can protect against injuries caused by harmful stimuli, such as pathogens and poisons. During this process, macrophages are key mediators of the inflammatory response that function by mobilizing the host defense against exogenous pathogens [1]. This mobilization occurs when macrophages are exposed to lipopolysaccharide (LPS) on the outer membranes of bacterial toxins [2]. Upon binding to macrophage pathogen-associated molecular pattern receptors, LPS activates various intracellular signaling pathways involved in the inflammatory burst, which includes the release of pro-inflammatory mediators and cytokines, such as interleukins, tumor necrosis factor-α (TNF-α), and inducible nitric oxide synthase (iNOS) [3,4]. Numerous inflammatory disorders, including atherosclerosis, chronic hepatitis, rheumatoid arthritis, and inflammatory brain diseases, have been linked to the pathophysiology of excessive inflammatory mediator production induced by activated macrophages [5]. Thus, the inhibition of these inflammatory mediators is a key target for the treatment of diseases associated with inflammation. 

In particular, nuclear factor (NF)-κB and mitogen-activated protein kinases (MAPKs) have a critical function in inflammation. Among transcription factors of the NF-κB family, the heterodimer NF-κB p50/p65 is bound to the inhibitor of κB (IκBα) and remains in its inactive form in the cytoplasm of unstimulated cells. However, in response to LPS stimulation, NF-κB is released and translocated into the nucleus, owing to the phosphorylation and degradation of IκBα [6,7]. The free NF-κB then translocates into the nucleus, where it binds at κB-binding sites in the promoter regions of target genes, inducing the transcription of genes encoding pro-inflammatory mediators such as COX-2, iNOS, TNF-α, IL-1β, and others [8]. Moreover, LPS induces the phosphorylation of proteins of the MAPK family, including extracellular signal-regulated kinase (ERK) 1/2, c-Jun NH2-terminal kinase (JNK), and p38, leading to the activation of NF-κB in macrophages [9]. Therefore, the inhibition of NF-κB and MAPK pathways might contribute to anti-inflammatory effects. 

Synthetic steroids are generally used as a primary treatment for chronic inflammation. However, they are thought to weaken the immune system by interfering with the cytokine network [10]. As a result, for the prevention of inflammatory diseases, effective natural therapeutic approaches that can target intraplaque macrophages and their secreted products have been suggested. Some plants that have long been used worldwide in traditional medicine have been evaluated as potential platforms for developing new drugs to treat various diseases. Among them, *Abelmoschus manihot*, belonging to the *Malvaceae* family and commonly called Jinhuakui (in Chinese) and Geumhwagyu (in Korean), is an annual or perennial herbaceous flowering plant and an edible form of hibiscus [11]. Especially, the flowers and calyces of *A. manihot* are rich in bioactive substances, which have a variety of biological activities, including anti-oxidation, anti-tumor, hypolipidemic, hypoglycemic, antipyretic, anti-inflammatory, immune regulation, and other functions [12]. 

The fermentation process has been reported to enhance the biological properties of raw materials, including the antioxidant, anti-inflammatory, and anti-obesity properties, through the production of new bioactive compounds [13]. In particular, extremophiles, microorganisms that thrive under extreme environmental conditions are of biotechnological interest, as they produce extremozymes [14]. However, the effect of fermented *A. manihot* on macrophage-mediated inflammation and its underlying mechanism remains unknown.

Thus, in this study, we selected *Bacillus licheniformis* CP6, isolated from Tongyeong seawater in the Republic of Korea, which seems to have a qualified presumption of safety (QPS) status, specifically pertaining to microorganisms that do not raise safety concerns [15]. Hence, *A. manihot* was fermented with *B. licheniformis* CP6, and total phenolic and total flavonoid contents were investigated. Moreover, we evaluated the pharmacologic effects of *B. licheniformis* CP6-fermented *A. manihot* extract (FAME) on the production of inflammatory mediators, including nitric oxide (NO) and pro-inflammatory cytokines, in LPS-stimulated mouse macrophages and identified the mechanism underlying such effects based on the regulation of transcription, MAPK, and NF-κB signaling pathways. 

## 2. Materials and Methods

### 2.1. Bacterial Strains and Culture Conditions

Bacterial strains isolated from diverse extreme environments stored at the food biotechnology laboratory, Department of Bioscience, Silla University, were used. Strains with a QPS status for the fermentation of *Abelmoschus manihot* were selected among *Bacillaceae* species with extracellular protease and amylase activity. Strains were cultured on Nutrient agar (Difco, Sparks, MD, USA) supplemented with a 3% (*w*/*v*) NaCl concentration at the optimal temperature for the strains. 

### 2.2. Microbial Fermentation of Abelmoschus manihot

*Abelmoschus manihot* was purchased from Buan Dongjin Farm (Republic of Korea). After removing the pistil and stamen, separate flower petals and sepals were used in the experiment. The *A. manihot* petal and sepal were air-dried at 60 °C for 48 h and consequently crushed using a hood mixer (SMX-4000 DY, Cheonan, Republic of Korea). The fermentation medium was prepared using petal and sepal powder (1:1) for the experiment. Selected strains were cultured overnight on Nutrient agar supplemented with 3% (*w*/*v*) NaCl. Subsequently, colonies were grown in the same liquid medium for 12 h. Then, 1% of pre-seeding cultures were inoculated into the same liquid medium and grown for 9 h until the late exponential phase. Main culture fermentation was carried out using an *A. manihot* fermentation medium. The *A. manihot* fermentation medium was composed of 0.1% yeast extract (*w*/*v*), 3% NaCl (*w*/*v*), and 1% dried powder of *A. manihot* flower (*w*/*v*). Seed cultures were inoculated onto the main culture at a final OD_600 nm_ of 0.1 (~5.642 log colony-forming units (CFU)/mL) and incubated for 5 days. During incubation, aliquots were collected, and the growth of the strains was determined based on CFU/mL on Nutrient agar supplemented with 3% NaCl (*w*/*v*). We named the fermented product cultured by inoculating *B. licheniformis* CP6 into the above-mentioned main fermentation medium as FAME and the incubated product under the same conditions without inoculation as IAME. The experiment was repeated three times. 

### 2.3. Antioxidant Activity Measurement

The antioxidant activity was determined by estimating the reducing power and radical scavenging ability by performing a 2,2′-azinobis (3-ethylbenzothiazoline-6-sulfonic acid) (ABTS) radical scavenging activity assay. The radical cation scavenging capacity of the extract was examined against ABTS, generated based on the method in [16]. ABTS radical cation (ABTS^+^) was produced via the reaction of ABTS stock solution (7 mM) with 2.45 mM potassium persulfate (1:1) and by allowing the mixture solution to stand in a dark at room temperature for 12–16 h before use. Fresh ABTS^+^ solution was prepared each day. The ABTS^+^ solution was diluted with distilled water to an absorbance of 0.70 ± 0.02 at 734 nm. The ABTS^+^ reaction mixture contained 180 μL of ABTS^+^ and 20 μL of the antioxidant testing sample with an absorbance at 734 nm, recorded as A_sample_. The reaction was monitored for 2 min. The radical scavenging activity of each solution was then calculated as a percent according to the following equation:

Radical scavenging activity (%) = 1 − [(A_sample_ − A_Blank_)/(A_control_ − A_Blank_)] × 100

where A_Blank_ is the absorbance of the solvent. The experiment was repeated three times at each concentration. Ascorbic acid was used as a positive control.

### 2.4. Preparation of A. manihot Fermentation Extract

FAME (with inoculation) and IAME (without inoculation) were prepared via whole culture medium extraction. Twenty times (*w*/*v*) 70% ethanol was added to a 1 L culture medium (41 g of solid content) in an Erlenmeyer flask and extracted for 12 h at room temperature. Extraction was performed in triplicates. After extraction, the extract was centrifuged at 3000 rpm for 10 minutes, and the supernatant was filtered using Whatman filter paper No. 4 and evaporated at 40 °C. After completely removing the solvent with a lyophilizer, the extract was stored at −80 °C for further experiments. The yield of the final extract is IAME (obtaining 3.85 g based on 1L = yield rate 9.39%) and FAME (obtaining 5.58 g based on 1 L = yield rate 13.61%). 

### 2.5. Measurement of Phenolic and Flavonoid Contents

Total phenolic content (TPC) was determined via a colorimetric assay described by Folin-Denis [17]. Each sample (1 mg/mL) and different concentrations of tannic acid were prepared in a 96-well plate. An aliquot of 40 μL of FAME or standard was mixed with 20% Na_2_CO_3_ (60 μL, *w*/*v*), 1 M of Folin–Ciocalteu’s phenol reagent, and incubated at 37 °C in the dark for 30 min to facilitate the reaction. The absorbance of the reaction mixture was measured at 700 nm using a microplate reader (Thermo Scientific, MA, USA). The obtained data were expressed as standard equivalents (tannic acid equivalent (TAE) mg/1 g dry mass). 

Total flavonoid content was measured according to a method described previously [18]. A volume of 25 μL of FAME (1 mg/mL) and quercetin (standard) with different concentrations were mixed with 125 μL D.W in a 96-well plate. Next, 5% NaNO_3_ was added, and the mixture was kept in a shaker for 5 min. Then, 10% AlCl_3_ was added to the mixture and was kept shaking for 6 min. The reaction was stopped by adding 0.1 M NaOH and D.W. After mixing, the flavonoid content was detected at 517 nm using a microplate reader. The calculated data were expressed as standard equivalents (quercetin equivalent (QE) mg/1 g dry mass). 

### 2.6. Cell Culture

The Raw264.7 cell, a mouse macrophage line, was obtained from the Korea Cell Line Bank (Seoul, Republic of Korea). The cells were cultured at 37 °C in Dulbecco’s modified Eagle’s medium (GibcoBRL, Grand Island, NY, USA), supplemented with 100 U/mL penicillin, 100 μg/mL streptomycin, and 10% fetal bovine serum (GibcoBRL, Grand Island, NY, USA) in a 5% CO_2_ incubator. Raw264.7 cells were maintained via weekly passage and cells were utilized for experimentation at 60–80% confluence. 

### 2.7. Cell Viability

Cell viability was determined by performing an MTT (3-(4,5-dimethylthiazol-2-yl)-2,5-diphenyltetrazolium bromide) assay, as described previously [19]. Briefly, cells were seeded into flat-bottomed 96-well microplates at a density of 1 × 10^5^ cells/well and incubated for 24 h. Subsequently, the cells were incubated in a culture medium containing various concentrations of FAME and *A. manihot* extract (IAME) for 20 h. After treatment, the medium was discarded, and a fresh medium containing 5 mg/mL MTT was added per well and incubated for 4 h. Formazan salts were dissolved in DMSO (100 µL) and the absorbance was measured at 550 nm using a microplate reader.

### 2.8. Flow Cytometric Analysis of NO Production

NO levels were measured by performing flow cytometry using the Nitric Oxide kit (Luminex Co., Austin, TX, USA), according to the manufacturer’s instructions. Briefly, cells were seeded in 6-well plates at a density of 1 × 10^6^ cells/well. After 24 h, the cells were pretreated in a culture medium containing 100 μg/mL of FAME and IAME for 2 h, and then stimulated with LPS (1 μg/mL) for an additional 20 h. After treatment, cells were further incubated with an NO working solution at 37 °C for 30 min. Subsequently, cells were mixed and analyzed for NO production using the Guava Muse Cell Analyzer and Muse analysis software (Luminex co., Austin, TX, USA).

### 2.9. IL-1β, IL-6, and TNF-α Production

Raw264.7 cells were pre-incubated overnight in 6-well plates at a density of 1 × 10^6^ cells/well. After 24 h, the cells were pretreated in a culture medium containing 100 μg/mL of FAME and IAME for 2 h, and then stimulated with LPS (1 μg/mL) for an additional 20 h. IL-1β, IL-6, and TNF-α contents in the culture medium were measured using an enzyme immunoassay system (R&D System Inc., Minneapolis, MN, USA), according to the manufacturer’s instructions. The concentrations of IL-1β, IL-6, and TNF-α were calculated according to the standard curve using each of the recombinant cytokines in the ELISA kits (R&D system Inc., Minneapolis, MN, USA). 

### 2.10. Western Blotting

The cells were suspended in a lysis buffer (25 mM Tris-HCl pH 7.5, 250 mM NaCl, 1% v/v NP-40, 1 mM dithiothreitol, 1 mM phenylmethylsulfonyl fluoride (PMSF)) and a protein inhibitor cocktail (10 μg/mL aprotinin, 1 μg/mL leupeptin). The cells were centrifuged for 15 min at 20,000× *g* and 4 °C, and the supernatants were collected. The concentration of protein in the lysate was quantified using a Bio-Rad protein kit, according to the manufacturer’s protocol. The lysate was loaded onto 10% sodium dodecyl sulfate-polyacrylamide gels for separation by electrophoresis and transferred to nitrocellulose membranes. The membranes were blocked in 5% non-fat milk for 1 h and incubated overnight with primary antibodies (Cell Signaling Technology, MA, USA) at 4 °C. After washing, the membranes were then incubated for 1 h with a goat anti-mouse or goat anti-rabbit IgG horseradish peroxidase-conjugated secondary antibody at room temperature. Immunoreactive bands were visualized using an enhanced chemiluminescence (ECL) reagent and scanned by a luminescent image analyzer LAS-1000-plus (FUJIFILM, Tokyo, Japan). The band intensities were quantified using Multi Gauge V3.1, an image analysis program, (FUJIFILM Corporation, Valhalla, NY, USA), and normalized by the intensity of the corresponding β-actin band.

### 2.11. Statistical Analysis

Each experiment was performed in triplicate. The results were expressed as the mean ± SD. Statistical analysis, including analysis of variance, was performed using SAS 9.1 software. The values were evaluated by one-way analysis of variance (ANOVA), followed by post hoc Duncan’s multiple range test, and *p*-values of less than 0.05 were considered statistically significant. 

## 3. Results

### 3.1. Screening Bacterial Strains for A. manihot Fermentation

**Figure 1:**
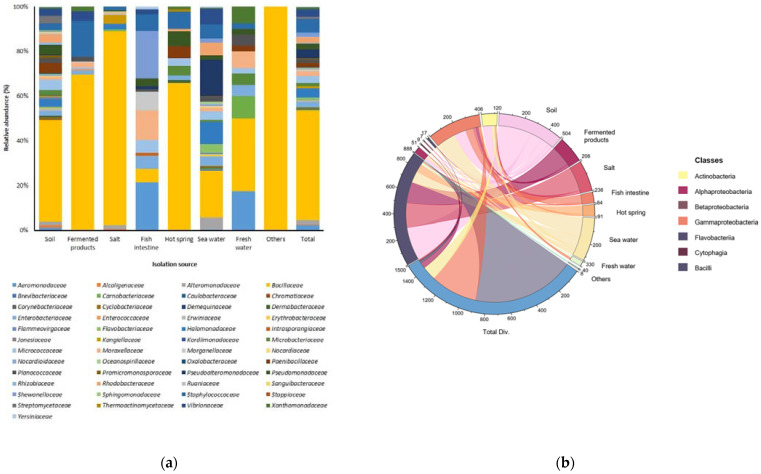
Relative abundances and bacterial diversity in specific environments. (**a**) Bacterial diversity at the family level; isolation sources were plotted on the horizontal axis, and the relative percentage of the family is visualized on the vertical axis, with each taxon shown as a specific color in the stacked columns. (**b**) A 3D chord diagram illustrating bacterial diversity at the class level; each arc shows overlapping diversity in specific environments. Total Div.: presenting the total diversity in specific environments (soil, fermented products, salt, fish intestine, hot spring, seawater, freshwater, and including other sources).

**Figure 2:**
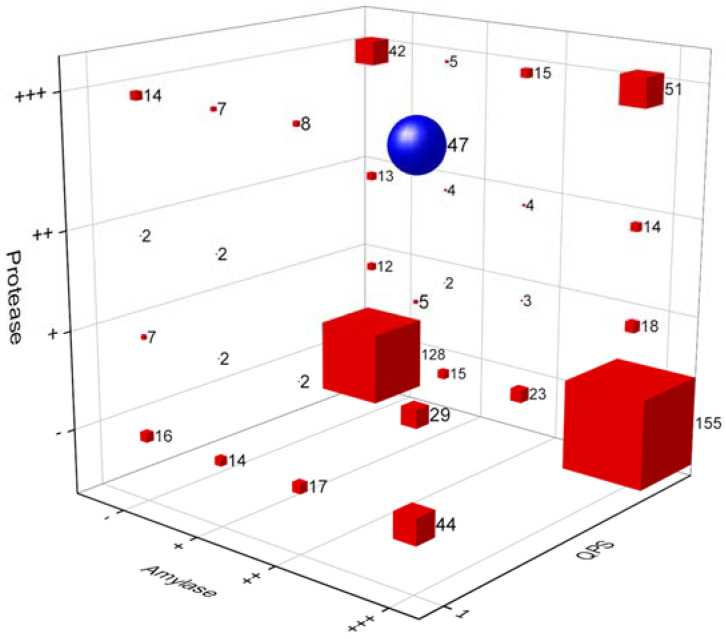
A 3D bubble plot of the primary selection of strains for the fermentation of *Abelmoschus manihot*, with a qualified presumption of safety (QPS) status among *Bacillaceae* species with extracellular protease and amylase activity. Blue circle: a group of 47 strains selected for primary fermentation. Values on the X-axis indicate amylase activity, the Y-axis is protease activity, and the Z-axis is the QPS status of the strains. Hydrolase (protease and amylase) enzyme activities were determined based on the agar diffusion method. The activity of the enzymes was measured based on the radius of the halo zone around colonies (+++: >7 mm, ++: 4–6 mm, +: 1–3 mm, -: no activity). Strains with a QPS status are labeled as 1 and other strains are labeled as 0.

**Figure 3:**
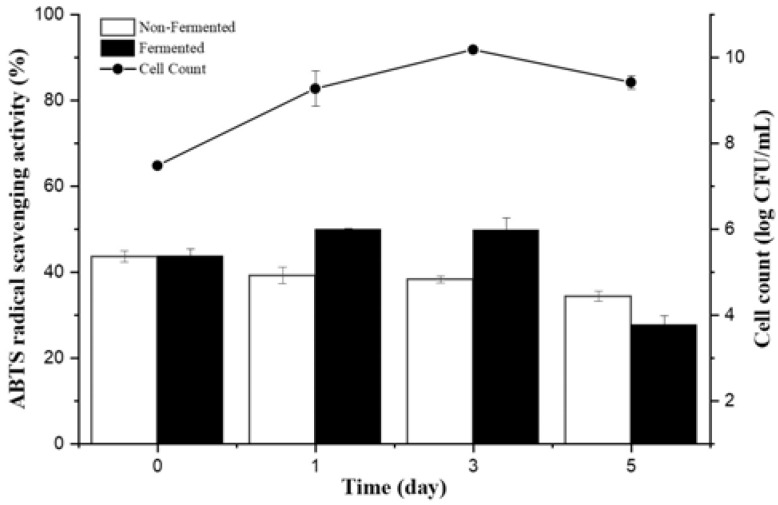
Change in bacterial viability and antioxidant activity of *Abelmoschus manihot* fermented with *Bacillus licheniformis* CP6 during fermentation.

In this study, we isolated 1500 bacterial strains (Tables S1 and S2) from various specific environments (Figure 1a,b). Of them, we selected suitable strains for the fermentation of *A. manihot* based on their QPS status, extracellular proteases, and amylase activity among *Bacillaceae* species. To select suitable strains for *A. manihot* fermentation, we grew 47 strains in *A. manihot* medium, which yielded only six strains (CP-6, CP-7, CP-35, CP-37, CP-39, and CP-42) with an average concentration of ~7.95 log CFU/mL (Figure 2). Additionally, we examined their antioxidant activity by measuring the ABTS radical scavenging activity of the fermented *A. manihot* at 0, 1, 3, and 5 days of fermentation. The results showed that four strains promoted the antioxidant activity of *A. manihot*. However, the *B. licheniformis* CP6 strain exhibited relatively better performance, in terms of ABTS activity (50.0%), compared to that of the control and was maintained for 2 days (Table 1). To determine the optimal fermentation time for CP6, we investigated the growth pattern of CP6 in *A. manihot* medium for 60 h and determined the ABTS radical scavenging activity (Figure 3). The *B. licheniformis* CP6 isolate was deposited into the Korean Collection for Type Cultures (KCTC), with culture collection number KCTC18811P.

### 3.2. Effects of FAME on Cytotoxicity and Morphological Changes in Raw264.7 Cells

Total phenolic contents of FAME and IAME were 29.52 mg and 20.8 mg TAE per 1 g of dry mass, respectively. The flavonoid contents of FAME and IAME were 33.12 mg and 18.32 mg QE per 1 g dry mass, respectively (data not shown). Interestingly, phenolic and flavonoid contents were significantly more abundant in FAME than in IAME. In particular, the flavonoid content in FAME was 2-fold higher than that in IAME. 

**Figure 4:**
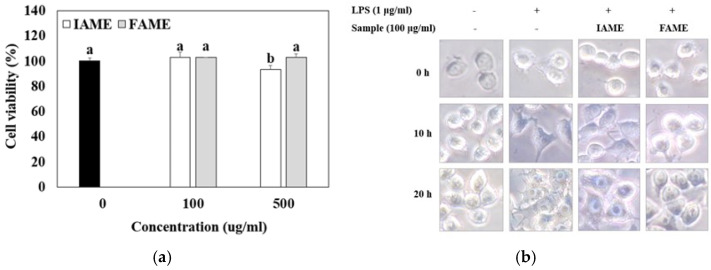
Effects of IAME and FAME on (**a**) cytotoxicity and (**b**) morphological changes in Raw264.7 cells. (**a**) Raw264.7 cells were incubated with IAME and FAME for 20 h. The cell viability of Raw264.7 cells was assessed by performing an MTT assay. (**b**) Raw264.7 cells were pretreated with IAME and FAME for 2 h and then stimulated with lipopolysaccharide (LPS) for 10 or 20 h on cover slides. Morphological changes in Raw264.7 were visualized via optical microscopy (×400). IAME, *Abelmoschus manihot* extract; *A. manihot* extract fermented by *Bacillus licheniformis* CP6.

The cytotoxic effect of FAME and IAME on the viability of Raw264.7 cells was investigated by performing an MTT assay. As shown in Figure 4a, exposing the Raw264.7 cells to 100 or 500 μg/mL of FAME for 20 h did not affect the viability of cells. However, the viability of cells exposed to 500 μg/mL of IAME for 20 h was reduced to 93% of that of the controls. The results showed that FAME and IAME had no cytotoxic effects at any of the concentrations examined. 

Meanwhile, LPS is known to cause the morphological transformation of macrophage Raw264.7 cells [20]. Thus, whether FAME and IAME affect LPS-induced morphological changes in Raw264.7 cells was investigated. As shown in Figure 4b, the untreated normal Raw264.7 cells were round, with smooth cell edges, and without pseudopodia, but those stimulated with LPS for 20 h showed characteristics of macrophage activation, such as an increase in cell size and spindle-shaped pseudopodia. FAME treatment reversed this process, and thus, the original round shape of cells was restored. Thus, fermentation was observed to increase the anti-inflammatory properties of IAME. 

### 3.3. Effects of FAME on the Inhibition of NO Production in LPS-Stimulated Raw264.7 Cells

**Figure 5:**
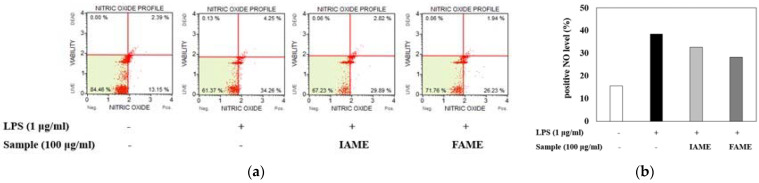
Effects of IAME and FAME on nitric oxide (NO) production in lipopolysaccharide (LPS)-stimulated Raw264.7 cells. (**a**) Quantitative measurements of NO production were performed via cytometry, using the Muse Nitric Oxide kit. Raw264.7 cells were pretreated with IAME and FAME (100 μg/mL) for 2 h and then stimulated with LPS (1 μg/mL) for 20 h. (**b**) The graph shows the NO percent positivity. IAME, *Abelmoschus manihot* extract; FAME, *A. manihot* extract fermented by *Bacillus licheniformis* CP6.

During immune responses, NO is a crucial physiological messenger [21]. To examine the inhibitory effect of FAME on LPS-induced NO production, Raw264.7 cells were treated with LPS in the presence or absence of the extract for 20 h. As shown in Figure 5, the number of NO (+) cells was elevated by LPS treatment. However, when pretreated with FAME and IAME, LPS-induced NO (+) cell production was inhibited. Moreover, FAME decreased NO production more than IAME. These findings demonstrated that fermentation can increase the anti-inflammatory properties of IAME. 

### 3.4. Effects of FAME on the Production of Inflammatory Cytokines in LPS-Stimulated Raw264.7 Cells

**Figure 6:**
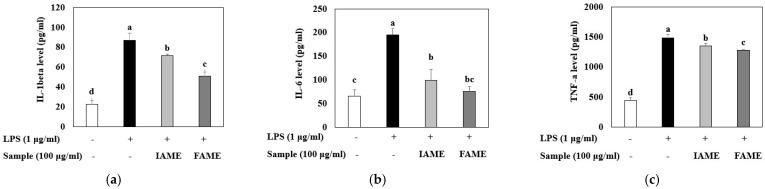
Effects of IAME and FAME on lipopolysaccharide (LPS)-stimulated IL-1β, IL-6, and TNF-α release in Raw264.7 cells. Raw264.7 cells were pretreated with IAME and FAME (100 μg/mL) for 2 h and then stimulated with LPS (1 μg/mL) for 20 h. After incubation, the levels of (**a**) IL-1β, (**b**) IL-6, and (**c**) TNF-α present in the supernatants were measured using ELISA kits. Each value is expressed as the mean ± SD (*n* = 3). ^a–d^ Values with different letters are different at *p* < 0.05, as analyzed by Duncan’s multiple range test. IAME, *Abelmoschus manihot* extract; *A. manihot* extract fermented by *Bacillus licheniformis* CP6.

Because LPS can induce pro-inflammatory cytokine production, we evaluated the effects of FAME and IAME on the levels of cytokine (IL-1β, IL-6, and TNF-α) production in LPS-stimulated macrophages using ELISA kits. The results revealed that IL-1β, IL-6, and TNF-α levels were increased in the culture media of LPS-stimulated cells; however, these increases were inhibited by treatment with FAME and IAME (Figure 6). In particular, compared to those with IAME treatment, FAME effectively inhibited the levels of cytokines. These results suggested that IAME modified by fermentation has elevated inhibitory activity with respect to pro-inflammatory cytokine production. 

### 3.5. Effects of FAME on iNOS and COX-2 Expression in LPS-Stimulated Raw264.7 Cells

**Figure 7:**
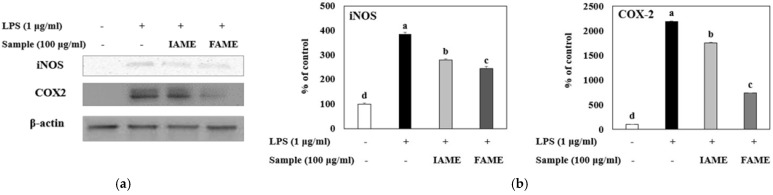
Effects of IAME and FAME on iNOS and COX-2 protein expression in lipopolysaccharide (LPS)-stimulated Raw264.7 cells. Raw264.7 cells were pretreated with IAME and FAME (100 μg/mL) for 2 h and then stimulated with LPS (1 μg/mL) for 20 h. Equal amounts of cell lysates (30 μg) were subjected to electrophoresis and analyzed for iNOS and COX-2 expression by performing Western blotting. Actin was used as an internal control. (**a**) iNOS and COX-2 protein expression. (**b**) Expression levels of iNOS and COX-2. Each value is expressed as the mean ± SD (*n* = 3). ^a–d^ Values with different letters are different at *p* < 0.05, as analyzed by Duncan’s multiple range test. IAME, *Abelmoschus manihot* extract; *A. manihot* extract fermented by *Bacillus licheniformis* CP6.

Next, we investigated whether the inhibitory effects of FAME on NO production are related to the modulation of the expression of iNOS and COX-2, which mediate their synthesis. iNOS and COX-2 genes are usually strongly activated in response to immune stimulators, such as LPS. Further, iNOS and COX-2 mediate inflammatory reactions and catalyze NO synthesis [22]. As shown in Figure 7, IAME and FAME effectively attenuated protein expressions of iNOS and COX-2, as compared to the LPS-stimulated cells. Moreover, the inhibitory effects of FAME against iNOS and COX-2 protein expression were higher than that of IAME. These results indicated that FAME represses both iNOS and COX-2 protein expression, inhibiting NO production.

### 3.6. Effects of FAME on the NF-κB Signaling Pathway in LPS-Stimulated Raw264.7 Cells

**Figure 8:**
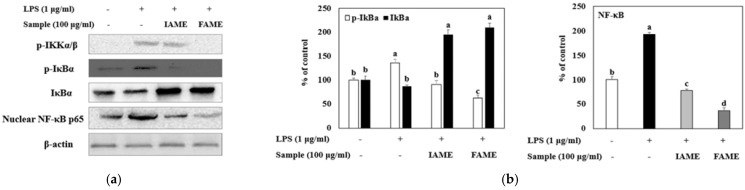
Effects of IAME and FAME on lipopolysaccharide (LPS)-stimulated NF-κB signaling pathway. Raw264.7 cells were pretreated with IAME and FAME (100 μg/mL) for 2 h and then stimulated with LPS (1 μg/mL) for 20 h. Equal amounts of cell lysates (30 μg) were subjected to electrophoresis and analyzed for p-IKKα/β, p-IκBα, IκBα, and NF-κB p65 expression by performing Western blotting. Actin was used as an internal control. (**a**) p-IKKα/β, p-IκBα, IκBα, and NF-κB p65 protein expression. (**b**) Expression levels of p-IκBα, IκBα, and NF-κB p65. Each value is expressed as the mean ± SD (*n* = 3). ^a–d^ Values with different letters are different at *p* < 0.05, as analyzed by Duncan’s multiple range test. IAME, *Abelmoschus manihot* extract; *A. manihot* extract fermented by *Bacillus licheniformis* CP6.

Inflammatory stimuli, such as LPS, activate NF-κB, mediating the transcriptional induction of pro-inflammatory gene expression [23]. We investigated whether FAME and IAME would exert anti-inflammatory effects by modulating NF-κB signaling in LPS-stimulated macrophages. As shown in Figure 8a and b, LPS stimulation resulted in a notable increase in p65 and IκBa phosphorylation. Further, the LPS-induced nuclear translocation of p65 was inhibited modestly by IAME, but the inhibitory effect of FAME was increased. In addition, FAME blocked the phosphorylation of IκBa in the cytosol more than IAME. These results showed that FAME strongly prevents IκBa degradation and NF-κB activation to a greater degree than IAME. Moreover, FAME effectively inhibits the NF-κB signaling pathway in LPS-stimulated Raw264.7 cells. 

### 3.7. Effects of FAME on the MAPK Signaling Pathway in LPS-Stimulated Raw264.7 Cells

**Figure 9:**
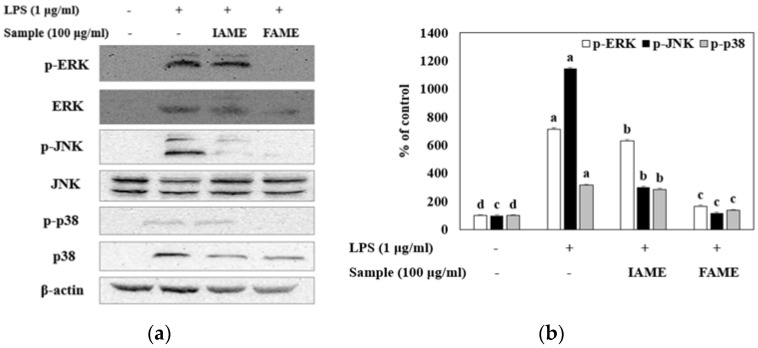
Effect of IAME and FAME on lipopolysaccharide (LPS)-stimulated MAPK signaling pathway. Raw264.7 cells were pretreated with IAME and FAME (100 μg/mL) for 2 h and then stimulated with LPS (1 μg/mL) for 20 h. Equal amounts of cell lysates (30 μg) were subjected to electrophoresis and analyzed for p-ERK1/2, ERK1/2, p-JNK, JNK, p-p38, and p38, expression by performing Western blotting. Actin was used as an internal control. (**a**) p-ERK1/2, ERK1/2, p-JNK, JNK, p-p38, and p38 protein expression. (**b**) Expression levels of p-ERK1/2, p-JNK, and p-p38. (**c**) Expression levels of ERK, JNK, and p38. Each value is expressed as the mean ± SD (*n* = 3). ^a–d^ Values with different letters are different at *p* < 0.05, as analyzed by Duncan’s multiple range test. IAME, *Abelmoschus manihot* extract; *A. manihot* extract fermented by *Bacillus licheniformis* CP6.

MAPK molecules are widely recognized as key signaling mediators that regulate cellular processes, including the release of pro-inflammatory cytokines during the inflammatory response [24]. To evaluate whether FAME and IAME could affect MAPK signaling, we analyzed their effects on the phosphorylation of MAPKs (ERK, JNK, and p38) in LPS-stimulated Raw264.7 cells by performing Western blotting. Figure 9 shows the LPS-induced phosphorylation of MAPKs. After FAME and IAME treatment, the protein expression levels of p-ERK, p-JNK, and p-p38 were decreased in LPS-induced Raw264.7 cells. Interestingly, compared to those with IAME treatment, FAME significantly suppressed the phosphorylation of all MAPKs upon LPS stimulation. These findings indicated that the fermentation of IAME by *B. licheniformis* CP6 augments its repressive effect on MAPK activation in LPS-stimulated Raw264.7 cells.

## 4. Discussion

Fermentation is the process wherein microorganisms produce substances that are useful to humans [25]. Recent research has focused on applying these fermentation techniques to natural products to obtain more useful and improved physiological activity. During fermentation, the microorganisms associated with fermentation break down plant cell walls, allowing biologically active compounds to be released during the next extraction stage. Among these organisms, extremophilic microbes, which can survive in extreme conditions, are not only of ecological importance but are also gaining significance in biotechnological research and industrial applications [26]. Thus, in the current study, we isolated *B. licheniformis* CP6, a halophilic bacterium, from Tongyeong seawater. Further, we evaluated the anti-inflammatory effect of *A. manihot* fermented by *B. licheniformis* CP6 and determined its effects on NO production, inflammatory cytokine expression, the iNOS-mediated COX-2-induced pathway, the NF-κB pathway, and the MAPK pathway in LPS-stimulated Raw264.7 cells exposed to the fermented product.

In response to LPS, macrophages play an active role in inflammatory responses by releasing pro-inflammatory cytokines, including TNF-, IL-1, and IL-6, as well as inflammatory factors, such as NO, which recruit additional immune cells to infection or tissue injury sites [5]. Thus, inhibitors of these inflammatory molecules have been considered anti-inflammatory drug candidates. NO has been recognized as a crucial mediator and regulator of inflammatory responses, and it is generated by iNOS in activated inflammatory cells [27]. iNOS, the major protein involved in NO production, is closely linked to inflammatory disorders such as neurodegeneration, atherosclerosis, and septic shock [28]. COX-2 is regarded as a central mediator of inflammation, and its modulation was suggested as a potential therapeutic target. Furthermore, NO and iNOS have a direct effect on the enzymatic activity of COX-2 [29]. In this study, our data showed that FAME inhibited LPS-stimulated NO production more effectively than IAME. FAME strongly suppressed the protein expressions of iNOS and COX-2. A previous study reported that flavonoids inhibit NO production in Raw264.7 cells and that their inhibitory activity might be due to a reduction in iNOS expression [30]. Interestingly, in this study, phenolic and flavonoid contents were significantly more abundant in FAME than in IAME. Taken together, our findings demonstrated that fermentation increases the anti-inflammatory properties of IAME. Moreover, these findings demonstrated that the inhibition of NO generation mediated by FAME might be due to the suppression of iNOS and COX-2 upregulation during macrophage activation induced by LPS.

However, pro-inflammatory cytokines, such as IL-1β, IL-6, and TNF-α, are important mediators involved in a range of acute and chronic responses to inflammatory diseases [31]. IL-1β is a key pro-inflammatory cytokine, primarily released by macrophages, and it is thought to play a major role in rheumatoid arthritis pathogenesis [32]. IL-6 also acts as a powerful stimulator of the acute-phase response during the early phases of inflammation [33]. In addition, TNF-α is an inflammatory cytokine synthesized by macrophages that can stimulate the production or expression of IL-1β, IL-6, collagenase, and adhesion molecules [34]. Our data revealed that FAME has better inhibitory effects on the production of pro-inflammatory mediators than IAME, suggesting that the fermentation of IAME using *B. licheniformis* CP6 is effective for inflammation control. Ramiro et al. reported that isoquercitrin, a flavonoid, has potent anti-inflammatory activity and functions by reducing TNF-α levels [35]. Thus, we assume that FAME, which contains an increased amount of flavonoids, owing to fermentation, could be an effective inhibitor of pro-inflammatory cytokines, which are paramount in generating an inflammatory response in activated macrophages. 

NF-κB pathways are major signaling pathways that regulate the transcription of inflammatory mediators in macrophages. NF-κB is a key factor involved in the control of cell survival genes and induces the expression of inflammatory enzymes and cytokines, such as iNOS, COX-2, IL-1β, IL-6, and TNF-α [6,36]. In general, NF-κB and IκB bind together, forming a complex in the cytoplasm. After the LPS-induced phosphorylation and degradation of IκBα in the cytosol, NF-κB p65 subunits are free to translocate to the nucleus [37]. Following nuclear translocation, these subunits bind to the promoters of target genes, such as iNOS and COX-2, ultimately resulting in the release of many cytokines and chemokines [38]. In the present study, we revealed that the LPS-induced nuclear translocation of p65 was modestly inhibited by IAME but that FAME increased the inhibitory effect on the nuclear translocation of p65 through the elevated phosphorylation of IκBα, followed by its degradation. FAME dramatically attenuated the LPS-mediated upregulation of IKKα/β, IκB, and p65 phosphorylation, IκBα degradation, and the translocation of p65 from the cytoplasm and to the nucleus. According to one report, isoquercitrin is a major compound of *A. manihot*, and it was found to potently inhibit NF-κB activation in human basophil cells [39]. These results indicate that FAME strongly prevents NF-κB activation, more so than IAME. 

MAPK signaling pathways are also important factors in a variety of biological processes, including inflammation [40]. Extracellular signal-regulated kinase 1/2 (ERK 1/2), c-Jun NH2-terminal kinase (JNK), and p38 are intracellular serine/threonine protein kinases known as MAPKs [41]. LPS treatment results in the phosphorylation of JNK, ERK-1/2, and p38, leading to NF-κB activation in macrophages. In addition, MAPKs modulate the synthesis of cytokines and the expression of pro-inflammatory enzymes, such as iNOS, COX-2, IL-1β, and TNF-α [42,43]. Therefore, targeting MAPK pathways could be an effective anti-inflammatory therapeutic strategy. In this study, compared to those with IAME treatment, FAME significantly inhibited the LPS-stimulated phosphorylation of MAPKs. These findings suggested that fermentation by *B. licheniformis* CP6 enhances the suppressive effect of IAME on MAPK activities after LPS stimulation. 

Fermentation of plant-based products commonly increases the bioactive phenolic compounds and enhances their antioxidant activity [44]. In this study, fermentation was observed to increase the anti-inflammatory properties of IAME. Moreover, our results showed that FAME (fermented IAME) had a significant effect on flavonoid and polyphenol contents. Numerous biological actions of flavonoids have been demonstrated, including antioxidant, antibacterial, anti-inflammatory, anticancer, and hepatoprotective properties [45]. The fermentation of *A. manihot* is thought to cause various compositional and functional changes, as fermentation generates a large range of bioactive compounds through the metabolic activity of diverse microbes. Thus, we assume that FAME, containing an increased amount of flavonoids owing to fermentation by *B. licheniformis* CP6, could be an effective suppressor of inflammation. We assume that through biodegradation/bioconversion of *Abelmoshcus manihot* flowers by CP6, flowers were specifically decomposed, resulting increase in phenolic compounds [46]. On the other side, the phenolic compounds may be produced through the metabolic processes of *Bacillus licheniformis* CP6 [47,48].

Taken together, our findings demonstrated fermentation can increase the anti-inflammatory properties of IAME. FAME had potent anti-inflammatory activity, inhibiting the production of pro-inflammatory mediators such as NO, TNF-α, IL-1β, and IL-6, and the enzymes that govern their synthesis, namely iNOS and COX2. Furthermore, FAME strongly inhibited the LPS-induced activation of NF-κB translocation, IκBα degradation, and the MAPK pathway. These results suggest that FAME could be developed as a new anti-inflammatory therapeutic herbal medicine, without cytotoxicity, for the treatment of inflammation-related disorders. 

## Figures and Tables

**Table 1:** Antioxidant activity of six strains that showed growth of 1% *Abelmoschus manihot* medium.

No.	Isolate	Closest Match	Growth of 1% AM Medium (log CFU/mL) after 5 Days	Antioxidant Activity (ABTS) %				Total Soluble Protein Concentration (BCA) µg/mL			
				0D	1D	3D	5D	0D	1D	3D	5D
				1% AM Medium				1% AM Medium			
1	CP-6	*Bacillus licheniformis*	8.119	43.8 ± 1.6	50.0 ± 0.3	50.0 ± 2.7	27.8 ± 2.1	412.3 ± 15.7	243.58 ± 12.78	270.7 ± 6.3	149.3 ± 0.8
2	CP-7	*Bacillus subtilis*	7.949		47.1 ± 0.5	37.5 ± 2.7	34.0 ± 1.5		299.1 ± 5.3	205.6 ± 3.2	215.2 ± 0.4
3	CP-35	*Bacillus subtilis*	9.006		51.8 ± 3.1	44.9 ± 2.6	35.2 ± 2.4		344.0 ± 39.7	214.2 ± 4.4	195.0 ± 4.6
4	CP-37	*Bacillus subtilis*	8.130		30.2 ± 5.4	43.4 ± 2.2	30.9 ± 0.6		230.5 ± 8.1	268.4 ± 19.2	215.2 ± 2.2
5	CP-39	*Bacillus firmus*	7.845		43.7 ± 2.5	34.0 ± 0.5	36.0 ± 2.0		313.1 ± 11.5	216.8 ± 1.6	236.0 ± 3.8
6	CP-42	*Bacillus velezensis*	6.667		50.1 ± 4.9	42.3 ± 1.2	39.7 ± 4.0		236.8 ± 22.9	270.1 ± 2.1	248.5 ± 20.6

AM medium: *Abelmoschus manihot* jinhuakui medium.

## Data Availability

Not applicable.

## Supplementary Materials

The following supporting information can be downloaded at: https://www.mdpi.com/article/10.3390/nu15020309/s1, Table S1: List of bacterial strains isolated from specific environments; Table S2: Characteristics of the strains with excellent extracellular enzyme activities of CP series.
