# Supplementary material for: Fermentation of Abelmoschus manihot Extract with Halophilic Bacillus licheniformis CP6 Results in Enhanced Anti-Inflammatory Activities
Source: Nutrients. 2023 Jan 7;15(2):309. doi: 10.3390/nu15020309 (PMC9864326; doi:10.3390/nu15020309)
Supplement: Supplementary file 1 [file nutrients-15-00309-s001.zip › nutrients-2088686-supplementary.pdf]

Table S1. List of bacterial strains isolated from specific environments.

No	Project name	Closest match	Source	Screening condition				Extracellular enzyme activity			
				Media	Temp. (°C)	pH	NaCl % (w/v)	Amylase	CMCase	Lipase	Protease
1		*Bacillus cereus*	Soil near Samcheonpo Fish Market	MA	37	7	3	-	-	-	+++
2	CP-01	*Bacillus megaterium*	Soil near Samcheonpo Fish Market	MA	37	7	3	+++	-	-	+++
3		*Bacillus cereus*	Soil near Samcheonpo Fish Market	MA	37	7	3	+++	-	+	++
4		*Acinetobacter radioresistens*	Soil near Samcheonpo Fish Market	MA	37	7	3	-	-	+++	-
5		*Acinetobacter radioresistens*	Soil near Samcheonpo Fish Market	MA	37	7	3	-	-	+++	-
6		*Acinetobacter radioresistens*	Soil near Samcheonpo Fish Market	MA	37	7	3	-	-	+++	-
7		*Acinetobacter radioresistens*	Soil near Samcheonpo Fish Market	MA	37	7	3	-	-	+++	-
8		*Bacillus circulans*	Soil near Samcheonpo Fish Market	MA	37	7	3	+++	-	-	-
9		*Pseudomonas luteola*	Soil near Samcheonpo Fish Market	MA	37	7	3	-	-	-	++
10		*Enterobacter aerogenes*	Yeosu dolsan park soil	MA	37	7	3	-	-	-	-
11		*Enterobacter aerogenes*	Yeosu dolsan park soil	MA	37	7	3	-	-	-	-
12		*Bacillus megaterium*	Yeosu dolsan park soil	MA	37	7	3	+++	-	-	+++
13		*Staphylococcus sciuri*	Yeosu dolsan park soil	MA	37	7	3	-	-	-	+++
14		*Bacillus simplex*	Yeosu dolsan park soil	MA	37	7	3	-	-	-	-
15		*Enterobacter aerogenes*	Yeosu dolsan park soil	MA	37	7	3	-	-	-	-
16		*Bacillus toyonensis*	Yeosu dolsan park soil	MA	37	7	3	+++	-	-	+++
17		*Bacillus megaterium*	Yeosu dolsan park soil	MA	37	7	3	+++	-	-	+++
18		*Plautia stali*	Yeosu dolsan park soil	MA	37	7	3	-	-	-	-
19		*Bacillus aryabhattai*	Yeosu dolsan park soil	MA	37	7	3	-	-	-	+
20		*Plautia stali*	Yeosu dolsan park soil	MA	37	7	3	-	-	-	-
21		*Bacillus aryabhattai*	Yeosu dolsan park soil	MA	37	7	3	+++	-	-	+++
22		*Serratia marcescens*	Yeosu dolsan park soil	MA	37	7	3	-	-	-	-
23	CP-02	*Bacillus megaterium*	Yeosu dolsan park soil	MA	37	7	3	+++	-	+++	+++
24		*Acinetobacter radioresistens*	Yeosu dolsan park soil	MA	37	7	3	-	-	-	-
25		*Pantoea calida*	Yeosu dolsan park soil	MA	37	7	3	-	-	-	+
26		*Acinetobacter pittii*	Yeosu dolsan park soil	MA	37	7	3	-	-	+++	-
27		*Kordiimonas gwangyangensis*	Suncheon bay soil	MA	37	7	3	++	-	-	-
28		*Labrenzia alba*	Suncheon bay soil	MA	37	7	3	-	-	++	-
29		*Labrenzia aggregata*	Suncheon bay soil	MA	37	7	3	-	-	-	-
30		*Kangiella profundi*	Suncheon bay soil	MA	37	7	3	-	-	+++	+++
31		*Algoriphagus halophilus*	Suncheon bay soil	MA	37	7	3	-	-	+	++
32		*Echinicola shivajiensis*	Suncheon bay soil	MA	37	7	3	-	-	-	++
33		*Polymorphum gilvum*	Suncheon bay soil	MA	37	7	3	-	-	-	-
34	CP-11	*Bacillus hwajinpoensis*	Suncheon bay soil	MA	37	7	3	+++	-	++	+++
35		*Labrenzia alba*	Suncheon bay soil	MA	37	7	3	-	-	++	-
36		*Labrenzia alba*	Suncheon bay soil	MA	37	7	3	-	-	++	-
37		*Pleionea mediterranea*	Suncheon bay soil	MA	37	7	3	-	-	++	++

38		*Labrenzia alba*	Suncheon bay soil	MA	37	7	3	-	-	-	-
39		*Micrococcus aloeverae*	Suncheon bay soil	MA	37	7	3	-	+	+++	+++
40		*Pseudomonas tolaasii*	Suncheon bay soil	MA	37	7	3	++	-	-	-
41		*Micrococcus luteus*	Suncheon bay soil	MA	37	7	3	-	-	+	-
42		*Micrococcus aloeverae*	Suncheon bay soil	MA	37	7	3	-	-	+++	-
43		*Erythrobacter pelagi*	Chaeseokgang cliff soil	MA	37	7	3	-	-	-	-
44		*Vibrio parahaemolyticus*	Chaeseokgang cliff soil	MA	37	7	3	-	-	-	-
45		*Pseudoalteromonas shioyasakiensis*	Chaeseokgang cliff soil	MA	37	7	3	+++	-	+++	+++
46		*Bacillus massiliosenegalensis*	Chaeseokgang cliff soil	MA	37	7	3	-	-	++	-
47		*Vibrio alginolyticus*	Chaeseokgang cliff soil	MA	37	7	3	+++	-	+	+
48		*Staphylococcus caprae*	Chaeseokgang cliff soil	MA	37	7	3	-	-	-	+
49		*Bacillus simplex*	Chaeseokgang cliff soil	MA	37	7	3	-	-	++	-
50		*Yokenella regensburgei*	Seocheon specialized market creek soil	MA	37	7	3	-	-	-	-
51		*Pseudomonas alcaligenes*	Seocheon specialized market creek soil	MA	37	7	3	-	-	+++	+++
52	CP-30	*Bacillus megaterium*	Seocheon specialized market creek soil	MA	37	7	3	+++	-	-	+++
53		*Micrococcus aloeverae*	Seocheon specialized market creek soil	MA	37	7	3	-	-	+++	-
54		*Staphylococcus saprophyticus*	Saemangeum fish market soil	MA	37	7	3	-	-	-	-
55		*Bacillus vietnamensis*	Saemangeum fish market soil	MA	37	7	3	+++	-	-	-
56		*Staphylococcus saprophyticus*	Saemangeum fish market soil	MA	37	7	3	-	-	-	-
57		*Bacillus aryabhattai*	Saemangeum fish market soil	MA	37	7	3	+++	-	-	+
58		*Kocuria dechangensis*	Saemangeum fish market soil	MA	37	7	3	++	-	+++	-
59		*Cronobacter zurichensis*	Saemangeum fish market soil	MA	37	7	3	-	-	-	-
60		*Arthrobacter crystallopoietes*	Saemangeum fish market soil	MA	37	7	3	-	+	+++	-
61		*Micrococcus luteus*	Saemangeum fish market soil	MA	37	7	3	-	-	-	+++
62		*Bacillus gibsonii*	Saemangeum fish market soil	MA	37	7	3	+++	-	-	-
63		*Staphylococcus epidermidis*	Saemangeum fish market soil	MA	37	7	3	-	-	++	++
64		*Leclercia adecarboxylata*	Miryang danjangcheon soil	MA	37	7	3	-	-	+	-
65		*Kosakonia cowanii*	Miryang danjangcheon soil	MA	37	7	3	-	-	+	-
66	CP-32	*Exiguobacterium indicum*	Miryang danjangcheon soil	MA	37	7	3	+++	-	+++	+++
67		*Microbacterium oleivorans*	Miryang danjangcheon soil	MA	37	7	3	-	-	+	+++
68		*Bacillus cereus*	Miryang danjangcheon soil	MA	37	7	3	+++	-	+++	+++
69		*Bacillus marisflavi*	Miryang danjangcheon soil	MA	37	7	3	+	-	++	+++
70	CP-33	*Exiguobacterium indicum*	Miryang danjangcheon soil	MA	37	7	3	+++	-	+++	+++
71		*Cronobacter sakazakii*	Miryang danjangcheon soil	MA	37	7	3	+	-	+	-
72		*Staphylococcus petrasii*	Miryang danjangcheon soil	MA	37	7	3	-	-	-	-
73		*Erythrobacter vulgaris*	Chaeseokgang cliff shell	MA	37	7	3	++	-	-	+
74		*Micrococcus luteus*	Chaeseokgang cliff shell	MA	37	7	3	-	-	-	+++
75		*Micrococcus luteus*	Chaeseokgang cliff shell	MA	37	7	3	-	-	-	+++
76		*Erythrobacter pelagi*	Chaeseokgang cliff shell	MA	37	7	3	-	-	-	+
77		*Erythrobacter pelagi*	Chaeseokgang cliff shell	MA	37	7	3	-	-	-	+
78		*Micrococcus aloeverae*	Chaeseokgang cliff shell	MA	37	7	3	-	+	++	+++
79		*Brevundimonas aurantiaca*	Chaeseokgang cliff shell	MA	37	7	3	+	-	-	-

80	*Erythrobacter pelagi*	Chaeseokgang cliff shell	MA	37	7	3	-	-	-	-
81	*Halobacillus profundi*	Gomso saltpan mud	MA	37	7	3	+++	-	+	-
82	*Halobacillus salinus*	Gomso saltpan mud	MA	37	7	3	-	-	-	-
83	*Micrococcus luteus*	Gomso saltpan mud	MA	37	7	3	-	+	++	-
84	*Micrococcus luteus*	Gomso saltpan mud	MA	37	7	3	-	-	-	-
85	*Micrococcus luteus*	Gomso saltpan mud	MA	37	7	3	-	-	-	-
86	*Micrococcus luteus*	Gomso saltpan salt	MA	37	7	3	-	-	++	-
87	*Bacillus niabensis*	Gomso saltpan salt	MA	37	7	3	+++	-	-	-
88	*Pseudomonas oryzihabitans*	Gomso saltpan salt	MA	37	7	3	-	-	++	+
89	*Desemzia incerta*	Gomso saltpan salt	MA	37	7	3	-	-	-	-
90	*Acinetobacter pittii*	Gomso saltpan salt	MA	37	7	3	-	-	++	-
91	*Acinetobacter pittii*	Gomso saltpan salt	MA	37	7	3	-	-	++	-
92	*Bacillus vietnamensis*	Gomso saltpan salt	MA	37	7	3	+++	-	-	-
93	*Bacillus boroniphilus*	Gomso saltpan salt	MA	37	7	3	+++	-	-	-
94	*Staphylococcus capitis*	Gomso saltpan salt	MA	37	7	3	+++	-	+++	-
95	*Staphylococcus cohnii subsp urealyticus*	Gomso saltpan salt	MA	37	7	3	-	-	++	-
96	*Staphylococcus aureus*	Gomso saltpan salt	MA	37	7	3	-	-	+++	++
97	*Staphylococcus epidermidis*	Gomso saltpan salt	MA	37	7	3	-	-	-	-
98	*Aeromonas bivalvium*	Samchonpo fish market gizzard shad guts	MA	37	7	3	+++	-	+++	+
99	*Psychrobacter sp.*	Samchonpo fish market gizzard shad guts	MA	37	7	3	-	-	+	-
100	*Janibacter hoylei*	Samchonpo fish market gizzard shad guts	MA	37	7	3	-	-	+++	+++
101	*Kurthia gibsonii*	Samchonpo fish market gizzard shad guts	MA	37	7	3	-	-	-	-
102	*Psychrobacter sp.*	Samchonpo fish market gizzard shad guts	MA	37	7	3	-	-	+	-
103	*Psychrobacter sp.*	Samchonpo fish market gizzard shad guts	MA	37	7	3	-	-	+	-
104	*Aeromonas popoffii*	Samchonpo fish market gizzard shad guts	MA	37	7	3	+++	-	+++	-
105	*Shewanella putrefaciens*	Samchonpo fish market gizzard shad guts	MA	37	7	3	-	-	+++	+
106	*Aeromonas bivalvium*	Samchonpo fish market gizzard shad guts	MA	37	7	3	+++	-	+++	+
107	*Aeromonas veronii*	Samchonpo fish market gizzard shad guts	MA	37	7	3	+++	-	+++	-
108	*Aeromonas bivalvium*	Samchonpo fish market gizzard shad guts	MA	37	7	3	+++	-	+	++
109	*Pseudomonas pseudoalcaligenes*	Samchonpo fish market gizzard shad guts	MA	37	7	3	-	-	+++	-

110	*Shewanella indica*	Samchonpo fish market eel guts	MA	37	7	3	-	-	-	+++
111	*Photobacterium damselae*	Samchonpo fish market eel guts	MA	37	7	3	-	-	+++	-
112	*Shewanella indica*	Samchonpo fish market eel guts	MA	37	7	3	-	-	-	-
113	*Proteus vulgaris*	Samchonpo fish market eel guts	MA	37	7	3	-	-	+++	-
114	*Proteus mirabilis*	Samchonpo fish market eel guts	MA	37	7	3	-	-	+++	-
115	*Proteus hauseri*	Samchonpo fish market eel guts	MA	37	7	3	-	-	-	-
116	*Shewanella indica*	Samchonpo fish market eel guts	MA	37	7	3	-	-	++	-
117	*Pseudomonas parafulva*	Samchonpo fish market eel guts	MA	37	7	3	-	-	-	-
118	*Citrobacter freundii*	Samchonpo fish market eel guts	MA	37	7	3	-	-	-	-
119	*Shewanella indica*	Samchonpo fish market eel guts	MA	37	7	3	-	-	++	-
120	*Shewanella algae*	Samchonpo fish market eel guts	MA	37	7	3	-	-	++	-
121	*Citrobacter freundii*	Samchonpo fish market eel guts	MA	37	7	3	-	-	-	-
122	*Proteus hauseri*	Samchonpo fish market eel guts	MA	37	7	3	-	-	-	-
123	*Fictibacillus phosphorivorans*	Samchonpo fish market eel guts	MA	37	7	3	+++	-	+++	-
124	*Micrococcus aloeverae*	Samchonpo fish market eel guts	MA	37	7	3	-	-	-	-
125	*Bacillus idriensis*	Samchonpo fish market eel guts	MA	37	7	3	+++	-	-	-
126	*Bacillus circulans*	Samchonpo fish market eel guts	MA	37	7	3	-	-	-	-
127	*Micrococcus antarcticus*	Samchonpo fish market eel guts	MA	37	7	3	+	-	-	-
128	*Desemzia incerta*	Samchonpo fish market eel guts	MA	37	7	3	-	-	-	-
129	*Psychrobacter sanguinis*	Samchonpo fish market yellow corvina guts	MA	37	7	3	-	-	-	-
130	*Shewanella algae*	Samchonpo fish market yellow corvina guts	MA	37	7	3	-	-	-	-
131	*Proteus mirabilis*	Samchonpo fish market yellow corvina guts	MA	37	7	3	-	-	-	-
132	*Psychrobacter pulmonis*	Samchonpo fish market yellow corvina guts	MA	37	7	3	-	-	+++	-
133	*Proteus hauseri*	Samchonpo fish market yellow corvina guts	MA	37	7	3	-	-	-	-
134	*Psychrobacter faecalis*	Samchonpo fish market yellow corvina guts	MA	37	7	3	-	-	++	-
135	*Psychrobacter pulmonis*	Samchonpo fish market yellow corvina guts	MA	37	7	3	-	-	++	-
136	*Shewanella indica*	Samchonpo fish market yellow corvina guts	MA	37	7	3	-	-	+	+++
137	*Psychrobacter pulmonis*	Samchonpo fish market yellow corvina guts	MA	37	7	3	-	-	+++	-
138	*Morganella morganii*	Samchonpo fish market yellow corvina guts	MA	37	7	3	-	-	-	-
139	*Aeromonas bivalvium*	Samchonpo fish market yellow corvina guts	MA	37	7	3	-	-	+	-
140	*Staphylococcus warneri*	Samchonpo fish market yellow corvina guts	MA	37	7	3	-	-	++	-

141	*Micrococcus luteus*	Samchonpo fish market yellow corvina guts	MA	37	7	3	-	-	+++	-
142	*Shewanella indica*	Samchonpo fish market yellow corvina guts	MA	37	7	3	-	-	+	-
143	*Micrococcus aloeverae*	Samchonpo fish market yellow corvina guts	MA	37	7	3	+++	-	+++	-
144	*Shewanella indica*	Samchonpo fish market yellow corvina guts	MA	37	7	3	-	-	+	-
145	*Salinicoccus hispanicus*	Samchonpo fish market yellow corvina guts	MA	37	7	3	-	-	+++	-
146	*Shewanella indica*	Samchonpo fish market yellow corvina guts	MA	37	7	3	-	-	+	-
147	*Shewanella algae*	Samchonpo fish market yellow corvina guts	MA	37	7	3	-	-	+	-
148	*Psychrobacter pulmonis*	Yeosu fish market eel guts	MA	37	7	3	-	-	+++	-
149	*Aeromonas bivalvium*	Yeosu fish market eel guts	MA	37	7	3	+++	-	+++	-
150	*Aeromonas bivalvium*	Yeosu fish market eel guts	MA	37	7	3	++	-	+++	-
151	*Aeromonas veronii*	Yeosu fish market eel guts	MA	37	7	3	+++	-	+++	++
152	*Aeromonas bivalvium*	Yeosu fish market eel guts	MA	37	7	3	+++	-	+++	-
153	*Shewanella seohaensis*	Yeosu fish market eel guts	MA	37	7	3	-	-	+++	-
154	*Aeromonas encheleia*	Yeosu fish market eel guts	MA	37	7	3	+++	-	+++	-
155	*Aeromonas bivalvium*	Yeosu fish market eel guts	MA	37	7	3	+++	-	+++	-
156	*Shewanella seohaensis*	Yeosu fish market eel guts	MA	37	7	3	-	-	+++	-
157	*Shewanella putrefaciens*	Seocheon specialized market several fish guts	MA	37	7	3	+	-	+++	-
158	*Shewanella algae*	Seocheon specialized market several fish guts	MA	37	7	3	-	-	++	-
159	*Staphylococcus caprae*	Seocheon specialized market several fish guts	MA	37	7	3	-	-	-	-
160	*Pseudomonas marincola*	Seocheon specialized market several fish guts	MA	37	7	3	-	-	-	-
161	*Acinetobacter venetianus*	Seocheon specialized market several fish guts	MA	37	7	3	-	-	+++	-
162	*Kluyvera cryocrescens*	Seocheon specialized market several fish guts	MA	37	7	3	-	-	++	-
163	*Micrococcus luteus*	Seocheon specialized market several fish guts	MA	37	7	3	-	-	-	+++
164	*Ewingella americana*	Seocheon specialized market several fish guts	MA	37	7	3	-	-	+++	+++
165	*Staphylococcus epidermidis*	Seocheon specialized market several fish guts	MA	37	7	3	-	-	++	+++
166	*Macrococcus caseolyticus*	Seocheon specialized market several fish guts	MA	37	7	3	-	-	-	+++

167	*Aeromonas media*	Seocheon specialized market several fish guts	MA	37	7	3	+++	-	+++	-
168	*Buttiauxella izardii*	Seocheon specialized market several fish guts	MA	37	7	3	-	-	-	-
169	*Bacillus herbersteinensis*	Seocheon specialized market several fish guts	MA	37	7	3	+++	-	-	-
170	*Macrococcus caseolyticus*	Seocheon specialized market several fish guts	MA	37	7	3	-	-	-	+++
171	*Aeromonas salmonicida*	Seocheon specialized market several fish guts	MA	37	7	3	+++	-	+++	+++
172	*Lelliottia nimipressuralis*	Seocheon specialized market several fish guts	MA	37	7	3	-	-	-	-
173	*Aeromonas media*	Seocheon specialized market eel guts	MA	37	7	3	+++	-	+++	-
174	*Vibrio plantisponsor*	Seocheon specialized market eel guts	MA	37	7	3	+++	-	-	-
175	*Shewanella xiamenensis*	Seocheon specialized market eel guts	MA	37	7	3	-	-	+++	-
176	*Aeromonas salmonicida*	Seocheon specialized market eel guts	MA	37	7	3	+++	-	+++	-
177	*Aeromonas media*	Seocheon specialized market eel guts	MA	37	7	3	+++	-	+++	-
178	*Pseudoalteromonas haloplanktis*	Seocheon specialized market eel guts	MA	37	7	3	++	-	+++	-
179	*Aeromonas salmonicida*	Seocheon specialized market eel guts	MA	37	7	3	+++	-	+++	-
180	*Shewanella baltica*	Seocheon specialized market eel guts	MA	37	7	3	-	-	+++	-
181	*[Haemophilus] piscium*	Seocheon specialized market eel guts	MA	37	7	3	+++	-	+++	-
182	*Vibrio alginolyticus*	Chaeseokgang cliff sea grass	MA	37	7	3	+++	-	+++	-
183	*Vibrio alginolyticus*	Chaeseokgang cliff sea grass	MA	37	7	3	+++	-	-	-
184	*Planococcus plakortidis*	Chaeseokgang cliff sea grass	MA	37	7	3	+++	-	-	-
185	*Vibrio alginolyticus*	Chaeseokgang cliff sea grass	MA	37	7	3	+++	-	-	-
186	*Vibrio rumoiensis*	Chaeseokgang cliff sea grass	MA	37	7	3	-	-	-	+
187	*Vibrio alginolyticus*	Chaeseokgang cliff sea grass	MA	37	7	3	+++	-	-	-
188	*Pseudoalteromonas prydzensis*	Chaeseokgang cliff sea grass	MA	37	7	3	-	-	-	-
189	*Vibrio alginolyticus*	Chaeseokgang cliff sea grass	MA	37	7	3	+++	-	-	-
190	*Vibrio rumoiensis*	Chaeseokgang cliff sea grass	MA	37	7	3	-	-	-	-
191	*Pseudoalteromonas mariniglutinosa*	Chaeseokgang cliff sea grass	MA	37	7	3	-	-	+++	-
192	*Bacillus subtilis*	Yeosu dolsan mustard kimchi	MA	37	7	3	+++	-	-	-
193	*Bacillus subtilis*	Yeosu dolsan mustard kimchi	MA	37	7	3	+++	-	-	-
194	*Bacillus subtilis*	Yeosu dolsan mustard kimchi	MA	37	7	3	+++	-	++	-
195	*Bacillus licheniformis*	Yeosu dolsan mustard kimchi	MA	37	7	3	-	-	-	++
196	*Staphylococcus warneri*	Tongyoung anchovy fermented seafood	MA	37	7	10	-	-	-	-
197	*Tetragenococcus halophilus*	Tongyoung anchovy fermented seafood	MA	37	7	10	-	-	-	-
198	*Planococcus plakortidis*	Suncheon bay crab preserved in soy sauce	MA	37	7	10	-	-	-	-
199	*Staphylococcus sciuri*	Suncheon bay crab preserved in soy sauce	MA	37	7	10	-	-	-	+++

200	*Vibrio rumoiensis*	Suncheon bay crab preserved in soy sauce	MA	37	7	10	-	-	+++	-
201	*Staphylococcus sciuri*	Suncheon bay crab preserved in soy sauce	MA	37	7	10	-	-	-	-
202	*Planococcus plakortidis*	Suncheon bay crab preserved in soy sauce	MA	37	7	10	-	-	-	+++
203	*Staphylococcus sciuri*	Suncheon bay crab preserved in soy sauce	MA	37	7	10	-	-	-	+++
204	*Vibrio rumoiensis*	Suncheon bay crab preserved in soy sauce	MA	37	7	10	-	-	+++	-
205	*Staphylococcus saprophyticus*	Suncheon bay crab preserved in soy sauce	MA	37	7	10	-	-	-	-
206	*Vibrio rumoiensis*	Suncheon bay crab preserved in soy sauce	MA	37	7	10	+++	-	+++	-
207	*Staphylococcus equorum*	Suncheon bay crab preserved in soy sauce	MA	37	7	10	-	-	-	-
208	*Vibrio rumoiensis*	Suncheon bay crab preserved in soy sauce	MA	37	7	10	+++	+++	-	-
209	*Vibrio rumoiensis*	Suncheon bay crab preserved in soy sauce	MA	37	7	10	+++	-	-	-
210	*Vibrio rumoiensis*	Suncheon bay crab preserved in soy sauce	MA	37	7	10	+++	+++	-	-
211	*Staphylococcus saprophyticus*	Suncheon bay crab preserved in soy sauce	MA	37	7	10	-	-	-	-
212	*Planococcus plakortidis*	Suncheon bay crab preserved in soy sauce	MA	37	7	10	-	-	-	+++
213	*Vibrio rumoiensis*	Suncheon bay crab preserved in soy sauce	MA	37	7	10	-	+++	-	-
214	*Planococcus rifietoensis*	Suncheon bay crab preserved in soy sauce	MA	37	7	10	-	-	-	+++
215	*Staphylococcus saprophyticus*	Suncheon bay crab preserved in soy sauce	MA	37	7	10	-	-	-	-
216	*Psychrobacter celer*	Suncheon bay crab preserved in soy sauce	MA	37	7	10	-	-	+++	+++
217	*Vibrio rumoiensis*	Suncheon bay crab preserved in soy sauce	MA	37	7	10	+++	-	-	+++
218	*Psychrobacter celer*	Suncheon bay crab preserved in soy sauce	MA	37	7	10	+++	-	+++	-
219	*Staphylococcus equorum*	Suncheon bay crab preserved in soy sauce	MA	37	7	10	-	-	-	-
220	*Oceanobacillus kimchii*	Suncheon bay crab preserved in soy sauce	MA	37	7	10	++	-	-	-

221		*Oceanobacillus kimchii*	Suncheon bay crab preserved in soy sauce	MA	37	7	10	-	-	-	-
222		*Vibrio rumoiensis*	Suncheon bay crab preserved in soy sauce	MA	37	7	10	+++	-	-	-
223		*Staphylococcus saprophyticus*	Suncheon bay crab preserved in soy sauce	MA	37	7	10	-	-	-	-
224		*Psychrobacter marincola*	Suncheon bay crab preserved in soy sauce	MA	37	7	10	-	-	-	-
225		*Bacillus stratosphericus*	Suncheon bay fermented food	MA	37	7	10	-	-	-	-
226		*Bacillus pumilus*	Suncheon bay fermented food	MA	37	7	10	+++	-	-	-
227		*Oceanobacillus manasiensis*	Suncheon bay fermented food	MA	37	7	10	-	-	-	+++
228		*Staphylococcus equorum*	Suncheon bay fermented food	MA	37	7	10	-	-	-	-
229		*Bacillus clausii*	Suncheon bay fermented food	MA	37	7	10	+++	-	-	-
230		*Staphylococcus equorum*	Suncheon bay fermented food	MA	37	7	10	+++	-	-	-
231		*Staphylococcus equorum*	Suncheon bay fermented food	MA	37	7	10	-	-	-	-
232		*Staphylococcus equorum*	Suncheon bay fermented food	MA	37	7	10	-	-	-	-
233		*Staphylococcus equorum*	Suncheon bay fermented food	MA	37	7	10	-	-	-	-
234		*Staphylococcus saprophyticus*	Gomso fermented food	MA	37	7	10	-	-	-	-
235	CP-03	*Bacillus clausii*	Gomso fermented food	MA	37	7	10	++	-	+++	+++
236	CP-04	*Bacillus licheniformis*	Gomso fermented food	MA	37	7	10	+++	-	-	+++
237	CP-05	*Bacillus stratosphericus*	Gomso fermented food	MA	37	7	10	-	-	+++	+++
238		*Bacillus clausii*	Gomso fermented food	MA	37	7	10	++	-	-	+++
239		*Bacillus stratosphericus*	Gomso fermented food	MA	37	7	10	-	-	-	-
240		*Bacillus atrophaeus*	Gomso fermented food	MA	37	7	10	+++	-	-	-
241		*Bacillus pumilus*	Gomso fermented food	MA	37	7	10	-	-	-	-
242		*Bacillus stratosphericus*	Gomso fermented food	MA	37	7	10	-	-	-	-
243		*Bacillus siamensis*	Gomso fermented food	MA	37	7	10	+++	-	-	-
244		*Bacillus pumilus*	Gomso fermented food	MA	37	7	10	-	-	-	-
245		*Bacillus mojavensis*	Gomso fermented food	MA	37	7	10	+++	-	-	-
246		*Bacillus clausii*	Gomso fermented food	MA	37	7	10	+	-	-	-
247	CP-06	*Bacillus licheniformis*	Tongyoung sea	MA	37	7	10	+++	-	-	+++
248		*Pseudoalteromonas tetraodonis*	Yeosu sea	MA	37	7	10	-	-	-	-
249		*Pseudoalteromonas tetraodonis*	Saemangeum Gareokdo port	MA	37	7	10	-	-	-	++
250		*Alteromonas macleodii*	Chaeseokgang cliff sea	MA	37	7	10	+++	-	+++	+++
251		*Staphylococcus aureus*	Chaeseokgang cliff sea	MA	37	7	10	-	-	-	-
252		*Vibrio alfinolyticus*	Chaeseokgang cliff sea	MA	37	7	10	+++	-	-	+++
253		*Alteromonas mediterranea*	Chaeseokgang cliff sea	MA	37	7	10	+++	-	-	+++
254		*Kocuria palustris*	Chaeseokgang cliff sea	MA	37	7	10	-	-	-	-
255		*Pseudomonas aestusnigri*	Yeosu Yeondeung cheonnamsangyo	MA	37	7	3	-	-	+++	-
256	CP-07	*Bacillus subtilis*	Yeosu Yeondeung cheonnamsangyo	MA	37	7	3	+++	-	+++	+++
257		*Pseudomonas marincola*	Yeosu Yeondeung cheonnamsangyo	MA	37	7	3	-	-	+++	-
258		*Pseudomonas aestusnigri*	Yeosu Yeondeung cheonnamsangyo	MA	37	7	3	-	-	+++	-
259		*Acinetobacter radioresistens*	Yeosu Yeondeung cheonnamsangyo	MA	37	7	3	-	-	+++	-

260	*Desemzia incerta*	Suncheon bay pond	MA	37	7	3	+++	-	+++	-
261	*Stenotrophomonas pavanii*	Suncheon bay pond	MA	37	7	3	+++	-	+++	-
262	*Acinetobacter radioresistens*	Suncheon bay pond	MA	37	7	3	+	-	+++	-
263	*Desemzia incerta*	Suncheon bay pond	MA	37	7	3	-	-	-	-
264	*Stenotrophomonas pavanii*	Suncheon bay pond	MA	37	7	3	-	-	+++	+++
265	*Stenotrophomonas maltophilia*	Suncheon bay pond	MA	37	7	3	-	-	+++	+++
266	*Pseudomonas parafulva*	Suncheon bay pond	MA	37	7	3	-	-	-	-
267	*Desemzia incerta*	Seocheon specialized market water	MA	37	7	3	-	-	-	-
268	*Bacillus pumilus*	Seocheon specialized market water	MA	37	7	3	-	-	-	+++
269	*Bacillus licheniformis*	Seocheon specialized market water	MA	37	7	3	-	-	-	+++
270	*Brevibacillus limnophilus*	Seocheon specialized market water	MA	37	7	3	+++	-	-	-
271	*Staphylococcus epidermidis*	Seocheon specialized market water	MA	37	7	3	+++	-	-	+++
272	*Micrococcus luteus*	Seocheon specialized market water	MA	37	7	3	-	-	-	+++
273	*Desemzia incerta*	Seocheon specialized market water	MA	37	7	3	+++	-	-	+++
274	*Microbacterium hydrocarbonoxydans*	Miryang danjangcheon	MA	37	7	3	-	-	-	-
275	*Acinetobacter pittii*	Miryang danjangcheon	MA	37	7	3	-	-	-	++
276	*Aeromonas media*	Miryang danjangcheon	MA	37	7	3	+++	-	+++	-
277	*Aeromonas hydrophila*	Miryang danjangcheon	MA	37	7	3	+++	-	+++	-
278	*Kosakonia cowanii*	Miryang danjangcheon	MA	37	7	3	-	-	-	-
279	*Aeromonas hydrophila*	Miryang danjangcheon	MA	37	7	3	+++	-	+++	-
280	*Aeromonas hydrophila*	Miryang danjangcheon	MA	37	7	3	+++	-	+++	-
281	*Aeromonas media*	Miryang danjangcheon	MA	37	7	3	-	-	-	-
282	*Aeromonas media*	Miryang danjangcheon	MA	37	7	3	+++	-	-	-
283	*Acinetobacter tandoii*	Miryang danjangcheon	MA	37	7	3	-	-	-	-
284	*Bacillus kokeshiiformis*	Tongyeong organic industry fish fertilizer	MA	60	7	3	-	-	+++	+
285	*Geobacillus thermodenitrificans*	Tongyeong organic industry fish fertilizer	MA	60	7	3	++	-	+	-
286	*Geobacillus thermodenitrificans*	Tongyeong organic industry fish fertilizer	MA	60	7	3	++	-	+	-
287	*Bacillus kokeshiiformis*	Tongyeong organic industry fish fertilizer	MA	60	7	3	-	-	++	++
288	*Bacillus kokeshiiformis*	Tongyeong organic industry fish fertilizer	MA	60	7	3	-	-	++	++
289	*Aeribacillus pallidus*	Tongyeong organic industry fish fertilizer	MA	60	7	3	+++	-	-	-
290	*Bacillus kokeshiiformis*	Tongyeong organic industry fish fertilizer	MA	60	7	3	+++	-	-	-
291	*Bacillus kokeshiiformis*	Tongyeong organic industry fish fertilizer	MA	60	7	3	-	-	-	-
292	*Bacillus kokeshiiformis*	Tongyeong organic industry fish fertilizer	MA	60	7	3	-	-	-	-

293		*Bacillus firmus*	yellow soil oak pyroligneous liquor	MA	45	7	3	+++	-	-	+++
294	CP-08	*Bacillus firmus*	yellow soil oak pyroligneous liquor	MA	45	7	3	+++	-	-	+++
295		*Bacillus aryabhattai*	Kangwon hardwood charcoal	MA	45	7	3	+++	-	-	-
296	CP-09	*Bacillus cereus*	Kangwon hardwood charcoal	MA	45	7	3	+++	-	-	+++
297		*Bacillus licheniformis*	Kangwon hardwood charcoal	MA	45	7	3	-	-	-	+
298		*Bacillus hisashii*	Kangwon hardwood charcoal	MA	45	7	3	+	-	-	-
299		*Bacillus licheniformis*	Kangwon hardwood charcoal	MA	45	7	3	+	-	-	+++
300		*Bacillus licheniformis*	Kangwon hardwood charcoal	MA	45	7	3	+	-	-	-
301		*Brachybacterium nesterenkovii*	Neungam carbonate hot spring	MA	45	7	3	+	-	-	-
302		*Bacillus firmus*	Neungam carbonate hot spring	MA	45	7	3	+	-	-	+++
303		*Staphylococcus warneri*	Neungam carbonate hot spring	MA	45	7	3	-	-	-	-
304		*Bacillus oceanisediminis*	Neungam carbonate hot spring	MA	45	7	3	++	-	-	-
305		*Microbacterium esteraromaticum*	Neungam carbonate hot spring	MA	45	7	3	+++	-	-	-
306		*Bacillus subtilis*	Neungam carbonate hot spring	MA	45	7	3	+	-	-	+++
307		*Microbacterium esteraromaticum*	Neungam carbonate hot spring	MA	45	7	3	-	-	-	-
308		*Microbacterium esteraromaticum*	Neungam carbonate hot spring	MA	45	7	3	++	-	-	-
309		*Brevibacillus agri*	Munjiang sulphur hot spring	MA	45	7	3	-	-	-	-
310		*Paenibacillus lautus*	Munjiang sulphur hot spring	MA	45	7	3	+	-	-	-
311		*Bacillus circulans*	Munjiang sulphur hot spring	MA	45	7	3	-	-	-	+++
312		*Bacillus circulans*	Munjiang sulphur hot spring	MA	45	7	3	-	-	-	+
313		*Micrococcus flavus*	Munjiang sulphur hot spring	MA	45	7	3	-	-	-	-
314	CP-10	*Bacillus flexus*	Munjiang sulphur hot spring	MA	45	7	3	+++	-	-	+++
315		*Micrococcus flavus*	Munjiang sulphur hot spring	MA	45	7	3	-	-	-	-
316		*Micrococcus flavus*	Munjiang sulphur hot spring	MA	45	7	3	-	-	-	-
317		*Bacillus circulans*	Munjiang sulphur hot spring	MA	45	7	3	-	-	-	+
318		*Bacillus hisashii*	Munjiang sulphur hot spring	MA	45	7	3	-	-	-	-
319		*Escherichia fergusonii*	Munjiang sulphur hot spring	MA	45	7	3	+	-	-	-
320		*Microbacterium esteraromaticum*	Munjiang sulphur hot spring	MA	45	7	3	+++	-	-	-
321		*Brevibacillus limnophilus*	Munjiang sulphur hot spring	MA	45	7	3	-	-	-	+++
322		*Bacillus thermoamylovorans*	Munjiang sulphur hot spring	MA	45	7	3	+++	-	+	++
323		*Bacillus canaveralius*	Munjiang sulphur hot spring	MA	45	7	3	-	-	-	+++
324		*Geobacillus toebii*	Munjiang sulphur hot spring	MA	45	7	3	+	-	-	-
325		*Bacillus badius*	Munjiang sulphur hot spring	MA	45	7	3	-	-	-	+++
326		*Pseudomonas guguanensis*	Munjiang sulphur hot spring	MA	45	7	3	-	-	-	-
327		*Pseudomonas guguanensis*	Munjiang sulphur hot spring	MA	45	7	3	+	-	-	-
328		*Pseudomonas alcaliphila*	Munjiang sulphur hot spring	MA	45	7	3	-	-	-	-
329		*Pseudomonas guguanensis*	Munjiang sulphur hot spring	MA	45	7	3	+	-	-	-
330		*Paracoccus versutus*	Munjiang sulphur hot spring	MA	45	7	3	-	-	-	-
331		*Pseudomonas alcaliphila*	Munjiang sulphur hot spring	MA	45	7	3	-	-	-	-
332		*Pseudomonas guguanensis*	Munjiang sulphur hot spring	MA	45	7	3	-	-	+	-
333		*Bacillus thermoamylovorans*	Deokgu hot spring	MA	45	7	3	+	-	-	+++
334		*Pseudomonas boreopolis*	Deokgu hot spring	MA	45	7	3	+++	-	-	+
335		*Bacillus circulans*	Baekam hot spring	MA	45	7	3	-	-	-	-

336		*Bacillus firmus*	Baekam hot spring	MA	45	7	3	-	-	-	+++
337		*Paenibacillus lautus*	Baekam hot spring	MA	45	7	3	-	++	-	++
338		*Bacillus circulans*	Baekam hot spring	MA	45	7	3	-	++	-	-
339		*Paenibacillus lactis*	Baekam hot spring	MA	45	7	3	-	-	-	+
340		*Bacillus siralis*	Baekam hot spring	MA	45	7	3	-	-	-	+++
341		*Bacillus thermoamylovorans*	Baekam hot spring	MA	45	7	3	-	-	-	+++
342		*Bacillus circulans*	Baekam hot spring	MA	45	7	3	-	-	-	+++
343		*Bacillus firmus*	Baekam hot spring	MA	45	7	3	-	-	-	+++
344	CP-12	*Bacillus hisashii*	Dongnae bathhouse	MA	45	7	3	++	+++	-	+
345		*Bacillus circulans*	Dongnae bathhouse	MA	45	7	3	-	+	+++	-
346		*Geobacillus stearothermophilus*	Neungam carbonate hot spring	MA	60	7	3	-	-	+++	+
347		*Bacillus thermoamylovorans*	Neungam carbonate hot spring	MA	60	7	3	-	-	-	++
348		*Caldibacillus debilis*	Neungam carbonate hot spring	MA	60	7	3	-	-	-	++
349		*Geobacillus stearothermophilus*	Neungam carbonate hot spring	MA	60	7	3	-	-	+	-
350		*Geobacillus stearothermophilus*	Munjiang sulphur hot spring	MA	60	7	3	+	-	+++	+
351		*Caldibacillus debilis*	Munjiang sulphur hot spring	MA	60	7	3	+	-	-	++
352		*Geobacillus stearothermophilus*	Munjiang sulphur hot spring	MA	60	7	3	+	-	++	-
353		*Bacillus thermoamylovorans*	Munjiang sulphur hot spring	MA	60	7	3	+	-	-	-
354		*Geobacillus stearothermophilus*	Munjiang sulphur hot spring	MA	60	7	3	+	-	++	++
355		*Geobacillus stearothermophilus*	Munjiang sulphur hot spring	MA	60	7	3	-	-	-	-
356		*Geobacillus stearothermophilus*	Munjiang sulphur hot spring	MA	60	7	3	-	-	-	-
357		*Geobacillus stearothermophilus*	Munjiang sulphur hot spring	MA	60	7	3	++	-	-	-
358		*Geobacillus stearothermophilus*	Munjiang sulphur hot spring	MA	60	7	3	++	-	-	-
359		*Bacillus thermoamylovorans*	Munjiang sulphur hot spring	MA	60	7	3	+	-	-	++
360		*Caldibacillus debilis*	Munjiang sulphur hot spring	MA	60	7	3	-	-	-	-
361		*Aeribacillus pallidus*	Munjiang sulphur hot spring	MA	60	7	3	-	-	-	-
362		*Aeribacillus pallidus*	Munjiang sulphur hot spring	MA	60	7	3	-	-	-	-
363		*Bacillus thermoamylovorans*	Munjiang sulphur hot spring	MA	60	7	3	-	-	-	-
364		*Caldibacillus debilis*	Munjiang sulphur hot spring	MA	60	7	3	-	-	-	-
365		*Aeribacillus pallidus*	Munjiang sulphur hot spring	MA	60	7	3	-	-	-	-
366		*Aeribacillus pallidus*	Munjiang sulphur hot spring	MA	60	7	3	-	-	-	-
367		*Geobacillus stearothermophilus*	Munjiang sulphur hot spring	MA	60	7	3	++	-	-	-
368		*Geobacillus stearothermophilus*	Munjiang sulphur hot spring	MA	60	7	3	-	-	+	+++
369		*Geobacillus stearothermophilus*	Munjiang sulphur hot spring	MA	60	7	3	++	-	+	-
370		*Geobacillus stearothermophilus*	Munjiang sulphur hot spring	MA	60	7	3	+	-	+	-
371		*Geobacillus stearothermophilus*	Munjiang sulphur hot spring	MA	60	7	3	+++	-	-	-
372		*Caldibacillus debilis*	Deokgu hot spring	MA	60	7	3	-	-	-	-
373		*Geobacillus stearothermophilus*	Deokgu hot spring	MA	60	7	3	+	-	-	-
374		*Geobacillus stearothermophilus*	Baekam hot spring	MA	60	7	3	-	-	-	-
375		*Bacillus hisashii*	Baekam hot spring	MA	60	7	3	++	-	-	-
376		*Bacillus thermoamylovorans*	Baekam hot spring	MA	60	7	3	+	-	-	-
377		*Aeribacillus pallidus*	Baekam hot spring	MA	60	7	3	-	-	-	-
378		*Geobacillus icigianus*	Baekam hot spring	MA	60	7	3	-	-	-	-

379		*Geobacillus stearothermophilus*	Baekam hot spring	MA	60	7	3	++	-	++	-
380		*Geobacillus kaustophilus*	Baekam hot spring	MA	60	7	3	-	-	+	-
381		*Geobacillus kaustophilus*	Dongnae bathhouse	MA	60	7	3	-	-	-	-
382		*Thermoactinomyces vulgaris*	Dongnae bathhouse	MA	60	7	3	-	-	-	-
383		*Bacillus licheniformis*	volcanic salts	MA	45	7	3	++	-	+	+++
384		*Oceanobacillus limi*	volcanic salts	MA	45	7	3	-	-	-	+++
385		*Bacillus licheniformis*	volcanic salts	MA	45	7	3	++	-	+	+++
386		*Oceanobacillus limi*	volcanic salts	MA	45	7	3	-	-	+	+++
387		*Bacillus sonorensis*	volcanic salts	MA	45	7	3	++	-	+	+++
388		*Bacillus licheniformis*	volcanic salts	MA	45	7	3	++	-	+	+++
389		*Virgibacillus senegalensis*	volcanic salts	MA	45	5.5	10	+++	-	+	+++
390		*Virgibacillus senegalensis*	volcanic salts	MA	45	5.5	10	-	-	-	+++
391		*Virgibacillus senegalensis*	volcanic salts	MA	45	5.5	10	-	-	-	+++
392		*Virgibacillus senegalensis*	volcanic salts	MA	45	5.5	10	++	-	-	-
393		*Bacillus sonorensis*	volcanic salts	MA	45	5.5	10	++	-	-	-
394		*Bacillus sonorensis*	volcanic salts	MA	45	5.5	10	++	-	-	-
395		*Bacillus sonorensis*	volcanic salts	MA	45	5.5	10	++	-	-	-
396		*Bacillus sonorensis*	volcanic salts	MA	45	5.5	10	+++	-	-	+++
397	CP-13	*Bacillus licheniformis*	volcanic salts	MA	45	8	3	+++	-	++	+++
398	CP-14	*Bacillus licheniformis*	volcanic salts	MA	45	8	3	+++	-	++	+++
399		*Bacillus licheniformis*	volcanic salts	MA	45	8	3	+++	-	-	+++
400		*Bacillus licheniformis*	volcanic salts	MA	45	8	3	++	-	-	+++
401		*Bacillus sonorensis*	volcanic salts	MA	45	8	3	-	-	-	-
402		*Gracilibacillus lacisalsi*	volcanic salts	MA	45	8	3	++	-	-	-
403		*Gracilibacillus thailandensis*	volcanic salts	MA	45	8	10	++	-	-	-
404		*Bacillus iranensis*	volcanic salts	MA	45	8	10	-	-	-	-
405		*Oceanobacillus limi*	volcanic salts	MA	45	8	10	-	-	-	+++
406		*Bacillus iranensis*	volcanic salts	MA	45	8	10	-	-	-	-
407		*Oceanobacillus limi*	volcanic salts	MA	45	8	10	-	-	-	+++
408		*Oceanobacillus limi*	volcanic salts	MA	45	8	10	-	-	-	+++
409		*Bacillus iranensis*	volcanic salts	MA	45	8	10	-	-	-	-
410		*Morganella psychrotolerans*	Fermented skate	MA	37	7	3	-	-	-	-
411		*Marinococcus luteus*	Fermented skate	MA	37	7	3	-	-	-	-
412		*Staphylococcus lentus*	Fermented skate	MA	37	7	3	-	-	-	-
413		*Micrococcus aloeverae*	Fermented skate	MA	37	7	3	-	+	+++	+
414		*Psychrobacter maritimus*	Fermented skate	MA	37	7	3	-	-	-	-
415		*Stenotrophomonas maltophilia*	Fermented skate	MA	37	7	3	-	-	+++	+++
416		*Staphylococcus cohnii*	Fermented skate	MA	37	7	3	-	-	+++	-
417		*Staphylococcus saprophyticus*	Fermented skate	MA	37	7	3	-	-	-	-
418		*Moellerella wisconsensis*	Fermented skate	MA	37	7	3	-	-	++	-
419		*Cellulophaga lytica*	Cheongsapo port	MA	37	7	3	-	-	-	+
420		*Bacillus atrophaeus*	Daebeon port seawater	MA	37	7	3	++	-	-	-
421		*Bacillus amyloliquefaciens*	Daebeon port seawater	MA	37	7	3	+++	-	-	-

422		*Micrococcus yunnanensis*	Daebeon port seawater	MA	37	7	3	-	-	-	++
423		*Escherichia fergusonii*	Daebeon port seawater	MA	37	7	3	-	-	-	-
424		*Planococcus citreus*	Daebeon port seawater	MA	37	7	3	-	-	-	+++
425		*Exiguobacterium mexicanum*	Daebeon port seawater	MA	37	7	3	+++	-	-	++
426		*Bacillus amyloliquefaciens*	Daebeon port seawater	MA	37	7	3	+++	-	-	-
427		*Klebsiella pneumoniae*	Daebeon port seawater	MA	37	7	3	-	-	-	-
428		*Bacillus licheniformis*	Dadaepo port seawater	MA	37	7	3	+++	-	-	+
429		*Enterobacter xiangfangensis*	Dadaepo port seawater	MA	37	7	3	-	-	-	-
430		*Shigella sonnei*	Dadaepo port seawater	MA	37	7	3	-	-	-	-
431		*Enterobacter ludwigii*	Dadaepo port seawater	MA	37	7	3	-	-	-	-
432		*Kocuria salsicia*	Dadaepo port seawater	MA	37	7	3	-	-	-	-
433		*Exiguobacterium himgiriensis*	Dadaepo port seawater	MA	37	7	3	++	-	-	-
434		*Cellulosimicrobium funkei*	Dadaepo port seawater	MA	37	7	3	++	-	-	+++
435	CP-15	*Bacillus cibi*	Dadaepo port seawater	MA	37	7	3	+++	-	-	+++
436		*Klebsiella oxitoca*	Dadaepo port seawater	MA	37	7	3	-	-	-	-
437		*Shigella sonnei*	Dadaepo port seawater	MA	37	7	3	-	-	-	-
438		*Escherichia fergusonii*	Dadaepo port seawater	MA	37	7	3	-	+++	-	-
439		*Kocuria salsicia*	Dadaepo port seawater	MA	37	7	3	-	-	-	-
440		*Bacillus megaterium*	Daejeo ecological park wetland soil	MA	37	7	3	+++	-	-	++
441		*Brachybacterium paraconglomeratum*	Daejeo ecological park wetland soil	MA	37	7	3	-	-	-	-
442		*Fictibacillus phosphorivorans*	Daejeo ecological park wetland soil	MA	37	7	3	++	-	-	+++
443		*Bacillus aryabhattai*	Daejeo ecological park wetland soil	MA	37	7	3	+++	-	-	++
444		*Bacillus soli*	Daejeo ecological park wetland soil	MA	37	7	3	+++	-	-	+++
445		*Streptomyces avermitilis*	Daejeo ecological park wetland soil	MA	37	7	3	-	-	-	+++
446		*Streptomyces phaeoluteigriseus*	Daejeo ecological park wetland soil	MA	37	7	3	-	-	-	++
447		*Bacillus marisflavi*	Daejeo ecological park wetland soil	MA	37	7	3	-	-	-	++
448	CP-16	*Bacillus cibi*	Daejeo ecological park wetland soil	MA	37	7	3	+++	-	-	+++
449		*Streptomyces filamentosus*	Daejeo ecological park wetland soil	MA	37	7	3	+++	-	-	-
450	CP-17	*Halobacillus trueperi*	Daejeo ecological park wetland soil	MA	37	7	3	++	-	-	+++
451		*Microbacterium testaceum*	Daejeo ecological park wetland soil	MA	37	7	3	-	-	-	++
452		*Serratia glossinae*	Daejeo ecological park wetland soil	MA	37	7	3	-	-	-	-
453		*Aeromonas media*	Daejeo ecological park wetland soil	MA	37	7	3	++	-	-	-
454		*Planococcus citreus*	Daejeo ecological park wetland soil	MA	37	7	3	-	-	-	+++
455		*Planococcus citreus*	Daejeo ecological park wetland soil	MA	37	7	3	-	-	-	++
456		*Halobacillus profundi*	volcanic salts	MA	37	7	3	-	-	-	-
457		*Oceanabacillus kimchii*	volcanic salts	MA	37	7	3	-	-	-	+
458		*Marinococcus halotolerans*	volcanic salts	MA	37	7	3	-	-	-	-
459		*Marinococcus luteus*	volcanic salts	MA	37	7	3	-	-	-	-
460		*Chromohalobacter canadensis*	volcanic salts	MA	37	7	3	+++	-	+	++
461		*Halobacillus profundi*	volcanic salts	MA	37	7	3	+	-	-	+++
462	CP-18	*Halobacillus dabanensis*	volcanic salts	MA	37	7	3	+++	-	-	+++
463	CP-19	*Bacillus toyonensis*	Pusan Amnam park area soil	MA	37	7	3	+++	-	-	+++

464		*Bacillus megaterium*	Pusan Amnam park area soil	MA	37	7	3	+++	-	-	++
465		*Fictibacillus phosphorivorans*	Pusan Amnam park area soil	MA	37	7	3	-	-	-	+++
466		*Solibacillus silvestris*	Pusan Amnam park area soil	MA	37	7	3	-	-	-	+++
467		*Streptomyces xiamenensis*	Pusan Amnam park area soil	MA	37	7	3	+++	-	-	+++
468		*Bacillus licheniformis*	Gamcheon port seawater	MA	37	7	3	-	-	-	+
469	CP-20	*Exiguobacterium mexicanum*	Gamcheon port seawater	MA	37	7	3	+++	-	-	+++
470		*Staphylococcus warneri*	Gamcheon port seawater	MA	37	7	3	-	-	-	+++
471		*Stenotrophomonas rhizophila*	Fermented skate	MA	37	7	3	-	-	-	+++
472		*Moellerella wisconsensis*	Fermented skate	MA	37	7	3	-	-	-	-
473		*Stenotrophomonas rhizophila*	Fermented skate	MA	37	7	3	-	-	-	+++
474		*Proteus hauseri*	Fermented skate	MA	37	7	3	-	-	-	-
475		*Enterobacter kobei*	Jagalchi market seawater	MA	37	7	3	-	-	-	-
476		*Oceanobacillus iheyensis*	Jagalchi market seawater	MA	37	7	3	-	-	-	+++
477		*Staphylococcus equorum*	Jagalchi market seawater	MA	37	7	3	-	-	-	=
478		*Bacillus firmus*	Jagalchi market seawater	MA	37	7	3	-	-	-	+++
479		*Pseudoalteromonas tetraodonis*	Jagalchi market seawater	MA	37	7	3	++	-	-	-
480		*Staphylococcus equorum*	Jagalchi market seawater	MA	37	7	3	-	-	-	-
481		*Exiguobacterium oxidotolerans*	Jagalchi market seawater	MA	37	7	3	+++	-	-	++
482	CP-21	*Halobacillus trueperi*	Korean Topan salt	MA	37	7	3	+++	-	++	+++
483		*Halobacillus alkaliphilus*	Korean Topan salt	MA	37	7	3	+++	-	-	++
484		*Halobacillus halophilus*	Korean Topan salt	MA	37	7	3	+++	-	-	+++
485		*Bacillus hwajinpoensis*	Korean Topan salt	MA	37	7	3	++	-	++	+++
486		*Pontibacillus chunwhensis*	Korean Topan salt	MA	37	7	3	+++	-	+++	+
487		*Maribacter aestuarii*	Minrak port seawater	MA	37	7	3	-	-	-	-
488		*Formosa spongicola*	Minrak port seawater	MA	37	7	3	-	-	-	+
489		*Kocuria salsicia*	Minrak port seawater	MA	37	7	3	-	-	-	-
490		*Staphylococcus hominis*	Minrak port seawater	MA	37	7	3	-	-	+++	-
491		*Staphylococcus warneri*	Minrak port seawater	MA	37	7	3	-	-	++	-
492		*Bacillus algicola*	Geojedo seawater	MA	25	7	3	++	-	-	-
493		*Bacillus lehensis*	Geojedo seawater	MA	25	7	3	+++	-	+	-
494	CP-22	*Bacillus horikoshii*	Dadaepo port seawater	MA	25	7	3	+++	-	+	+++
495		*Bacillus catenulatus*	Dadaepo port seawater	MA	25	7	3	+++	-	-	+++
496	CP-23	*Bacillus hwajinpoensis*	Dadaepo port seawater	MA	25	7	3	+++	-	+	+++
497		*Bacillus aquimaris*	Dadaepo port seawater	MA	25	7	3	++	-	+	++
498		*Bacillus marisflavi*	Dadaepo port seawater	MA	25	7	3	-	-	-	-
499		*Bacillus humi*	Wando seawater	MA	25	7	3	+++	-	-	-
500		*Bacillus amyloliquefaciens*	Wando seawater	MA	25	7	3	+++	-	-	-
501		*Bacillus halmapalus*	Wando seawater	MA	25	7	3	++	-	+	++
502		*Bacillus aquimaris*	Wando seawater	MA	25	7	3	++	-	+	++
503		*Bacillus gibsonii*	Wando seawater	MA	25	7	3	++	-	+	+++
504		*Bacillus amyloliquefaciens*	Wando seawater	MA	25	7	3	+	-	+	-
505	CP-24	*Bacillus ferrariarum*	Wando seawater	MA	25	7	3	++	-	+	+++
506	CP-25	*Bacillus alcalophilus*	Wando seawater	MA	25	7	3	+++	-	+	+++

507	CP-26	*Bacillus licheniformis*	Wando seawater	MA	25	7	3	++	+++	-	-
508	CP-27	*Exiguobacterium oxidotolerans*	Songjeong port seawater	MA	37	7	3	+++	-	-	+++
509		*Bacillus pumilus*	Samrak ecological park wetland water	MA	37	7	3	-	-	-	+++
510	CP-28	*Fictibacillus phosphorivorans*	Samrak ecological park wetland water	MA	37	7	3	++	-	-	+++
511		*Bacillus marisflavi*	Samrak ecological park wetland water	MA	37	7	3	-	-	-	+++
512		*Bacillus aryabhattai*	Samrak ecological park wetland water	MA	37	7	3	+++	-	-	++
513		*Bacillus licheniformis*	Samrak ecological park wetland water	MA	37	7	3	+++	-	-	+
514		*Kangiella koreensis*	Sinan taepyeong salt field	MA	37	7	3	-	-	+++	+
515		*Gramella aestuariivivens*	Sinan taepyeong salt field	MA	37	7	3	-	-	+++	+
516		*Marinobacter algicola*	Sinan taepyeong salt field	MA	37	7	3	-	-	+++	-
517		*Halomonas denitrificans*	Sinan taepyeong salt field	MA	37	7	3	-	-	-	-
518		*Marinobacterium nitratireducens*	Sinan taepyeong salt field	MA	37	7	3	-	-	+++	+
519	CP-29	*Halobacillus mangrovi*	Sinan taepyeong salt field	MA	37	7	3	+++	-	+	+++
520		*Salinimonas chungwhensis*	Sinan taepyeong salt field	MA	37	7	3	+	-	+++	-
521		*Halomonas ventosae*	Sinan taepyeong salt field	MA	37	7	3	-	-	-	-
522		*Marinobacter algicola*	Sinan taepyeong salt field	MA	37	7	3	-	-	-	-
523	CP-31	*Bacillus subtilis*	Sinan taepyeong salt field	MA	37	7	3	+++	-	-	+++
524		*Staphylococcus equorum*	Pusan baekyangsan bamboo forest soil	MA	37	7	3	-	-	-	-
525		*Micrococcus luteus*	estuary of Eulsukdo ecological park	MA	37	7	3	-	-	-	+
526		*Bacillus subtilis*	Korean traditional fermented food (soybean)	LB	37	7	3	+++	-	-	-
527		*Bacillus amyloliquefaciens*	Korean traditional fermented food (soybean)	LB	37	7	3	+	-	-	-
528		*Bacillus subtilis*	Korean traditional fermented food (soybean)	LB	37	7	3	+++	-	-	-
529		*Bacillus subtilis*	Korean traditional fermented food (soybean)	LB	37	7	3	+++	-	-	-
530		*Bacillus amyloliquefaciens*	Korean traditional fermented food (seafood)	LB	37	7	3	+++	-	-	-
531		*Bacillus subtilis*	Korean traditional fermented food (seafood)	LB	37	7	3	+++	-	-	-
532		*Bacillus amyloliquefaciens*	Korean traditional fermented food (seafood)	LB	37	7	3	+++	-	-	-
533		*Bacillus atrophaeus*	Korean traditional fermented food (soybean)	LB	37	7	3	+++	-	-	-
534		*Bacillus amyloliquefaciens*	Korean traditional fermented food (soybean)	LB	37	7	3	+++	-	-	-
535		*Exiguobacterium aestuarii*	Korean traditional fermented food (seafood)	LB	37	7	3	+	-	-	-
536		*Stenotrophomonas maltophilia*	Korean traditional fermented food (seafood)	LB	37	7	3	-	-	-	-
537		*Bacillus subtilis*	Korean traditional fermented food (soybean)	LB	37	7	3	+	-	-	-

538	*Bacillus amyloliquefaciens*	Korean traditional fermented food (soybean)	LB	37	7	3	++	-	-	-
539	*Bacillus paralicheniformis*	Korean traditional fermented food (soybean)	LB	37	7	3	+	-	-	-
540	*Bacillus amyloliquefaciens*	Korean traditional fermented food (soybean)	LB	37	7	3	++	-	-	-
541	*Bacillus toyonensis*	Korean traditional fermented food (soybean)	LB	37	7	3	++	-	-	-
542	*Bacillus paralicheniformis*	Korean traditional fermented food (soybean)	LB	37	7	3	+++	-	-	-
543	*Bacillus amyloliquefaciens*	Korean traditional fermented food (soybean)	LB	37	7	3	+++	-	-	-
544	*Bacillus paralicheniformis*	Korean traditional fermented food (soybean)	LB	37	7	3	+++	-	-	-
545	*Bacillus paralicheniformis*	Korean traditional fermented food (soybean)	LB	37	7	3	+++	-	-	-
546	*Bacillus subtilis*	Korean traditional fermented food (soybean)	LB	37	7	3	++	-	-	-
547	*Bacillus amyloliquefaciens*	Korean traditional fermented food (soybean)	LB	37	7	3	+	-	-	-
548	*Bacillus amyloliquefaciens*	Korean traditional fermented food (soybean)	LB	37	7	3	+	-	-	-
549	*Bacillus amyloliquefaciens*	Korean traditional fermented food (soybean)	LB	37	7	3	+	-	-	-
550	*Bacillus amyloliquefaciens*	Korean traditional fermented food (soybean)	LB	37	7	3	+++	-	-	-
551	*Bacillus amyloliquefaciens*	Korean traditional fermented food (soybean)	LB	37	7	3	+++	-	-	-
552	*Bacillus amyloliquefaciens*	Korean traditional fermented food (soybean)	LB	37	7	3	+++	-	-	-
553	*Bacillus atrophaeus*	Korea Southern sea water and sand	LB	37	7	3	+++	-	-	-
554	*Bacillus atrophaeus*	Korea Southern sea water and sand	LB	37	7	3	+++	-	-	-
555	*Bacillus atrophaeus*	Korea Southern sea water and sand	LB	37	7	3	+++	-	-	-
556	*Bacillus atrophaeus*	Korea Southern sea water and sand	LB	37	7	3	+++	-	-	-
557	*Bacillus atrophaeus*	Korea Southern sea water and sand	LB	37	7	3	+++	-	-	-
558	*Bacillus atrophaeus*	Korea Southern sea water and sand	LB	37	7	3	+++	-	-	-
559	*Bacillus atrophaeus*	Korea Southern sea water and sand	LB	37	7	3	+++	-	-	-
560	*Bacillus atrophaeus*	Korea Southern sea water and sand	LB	37	7	3	+++	-	-	-
561	*Bacillus atrophaeus*	Korea Southern sea water and sand	LB	37	7	3	+++	-	-	-
562	*Bacillus atrophaeus*	Korea Southern sea water and sand	LB	37	7	3	+++	-	-	-
563	*Bacillus atrophaeus*	Korea Southern sea water and sand	LB	37	7	3	+++	-	-	-
564	*Bacillus atrophaeus*	Korean traditional fermented food (soybean)	LB	37	7	3	+++	-	-	-

565	*Bacillus atrophaeus*	Korean traditional fermented food (soybean)	LB	37	7	3	+	-	-	-
566	*Bacillus atrophaeus*	Korean traditional fermented food (soybean)	LB	37	7	3	+	-	-	-
567	*Bacillus atrophaeus*	Korean traditional fermented food (soybean)	LB	37	7	3	+	-	-	-
568	*Bacillus atrophaeus*	Korean traditional fermented food (soybean)	LB	37	7	3	+++	-	-	-
569	*Bacillus atrophaeus*	Korean traditional fermented food (soybean)	LB	37	7	3	+	-	-	-
570	*Bacillus cereus*	Korea Southern sea soil	LB	37	7	3	++	-	-	-
571	*Staphylococcus hominis*	Korea Southern sea soil	LB	37	7	3	+++	-	-	-
572	*Bacillus cereus*	Korea Southern sea soil	LB	37	7	3	+++	-	-	-
573	*Bacillus atrophaeus*	Korea Southern sea soil	LB	37	7	3	++	-	-	-
574	*Bacillus marisflavi*	Korea Southern sea soil	LB	37	7	3	++	-	-	-
575	*Bacillus atrophaeus*	Korea Southern sea soil	LB	37	7	3	+	-	-	-
576	*Bacillus atrophaeus*	Korean traditional fermented food (seafood)	LB	37	7	3	+	-	-	-
577	*Stenotrophomonas maltophilia*	fermented honey	LB	37	7	3	+++	-	-	-
578	*Bacillus subtilis*	fermented honey	LB	37	7	3	+++	-	-	-
579	*Bacillus cereus*	Korea Southern sea soil	LB	37	7	3	++	-	-	-
580	*Bacillus atrophaeus*	Korea Southern sea soil	LB	37	7	3	+	-	-	-
581	*Bacillus atrophaeus*	Korean traditional fermented food (seafood)	LB	37	7	3	+	-	-	-
582	*Bacillus paralicheniformis*	Korea Southern seawater	LB	37	7	3	+++	-	-	-
583	*Bacillus subtilis*	Korea Southern seawater	LB	37	7	3	+	-	-	-
584	*[Brevibacterium] halotolerans*	Korea Southern seawater	LB	37	7	3	+++	-	-	-
585	*Bacillus aerius*	Korea Southern seawater	LB	37	7	3	+++	-	-	-
586	*Bacillus paralicheniformis*	Korea Southern seawater	LB	37	7	3	+++	-	-	-
587	*Bacillus paralicheniformis*	Korea Southern seawater	LB	37	7	3	+++	-	-	-
588	*Bacillus paralicheniformis*	Korea Southern seawater	LB	37	7	3	+++	-	-	-
589	*Bacillus paralicheniformis*	Korea Southern seawater	LB	37	7	3	++	-	-	-
590	*Bacillus paralicheniformis*	Korea Southern seawater	LB	37	7	3	++	-	-	-
591	*Bacillus paralicheniformis*	Korea Southern seawater	LB	37	7	3	++	-	-	-
592	*Bacillus subtilis*	Korean traditional fermented food (soybean)	LB	37	7	3	++	-	-	-
593	*Bacillus subtilis*	peatmoss (Silla univ. field)	LB	37	7	3	++	-	-	-
594	*Bacillus subtilis*	peatmoss (Silla univ. field)	LB	37	7	3	+	-	-	-
595	*Bacillus subtilis*	peatmoss (Silla univ. field)	LB	37	7	3	++	-	-	-
596	*Bacillus tequilensis*	peatmoss (Silla univ. field)	LB	37	7	3	+++	-	-	-
597	*Bacillus tequilensis*	peatmoss (Silla univ. field)	LB	37	7	3	+++	-	-	-
598	*Bacillus subtilis*	Korean traditional fermented food (soybean)	LB	37	7	3	++	-	-	-

599		*Bacillus atrophaeus*	Korean traditional fermented food (soybean)	LB	37	7	3	+++	-	-	-
600		*Bacillus amyloliquefaciens*	Korea Southern seawater	LB	37	7	3	+++	-	-	-
601		*Staphylococcus capitis*	Aragonite hot spring	MA	37	7	10	-	-	+	-
602		*Staphylococcus epidermidis*	Aragonite hot spring	MA	37	7	10	-	-	-	++
603		*Staphylococcus saprophyticus subsp. Saprophyticus*	Aragonite hot spring	MA	37	7	10	-	-	++	-
604		*Staphylococcus haemolyticus*	Aragonite hot spring	MA	37	7	10	-	-	+	++
605		*Staphylococcus hominis subsp. Novobiosepticus*	Aragonite hot spring	MA	37	7	5	-	-	-	+
606		*Staphylococcus hominis*	Aragonite hot spring	MA	37	7	5	-	-	-	-
607		*Staphylococcus hominis subsp. Novobiosepticus*	Unjin port seawater	MA	37	7	10	-	-	-	-
608		*Staphylococcus epidermidis*	Unjin port seawater	MA	37	7	10	-	-	-	+
609		*Staphylococcus hominis*	Unjin port seawater	MA	37	7	10	-	-	-	+
610		*Micrococcus aloeverae*	Unjin port seawater	MA	37	7	5	-	-	-	+
611		*Pseudoalteromonas agarivorans*	Unjin port seawater	MA	37	7	5	-	-	-	-
612		*Pseudoalteromonas agarivorans*	Unjin port seawater	MA	37	7	5	-	-	-	-
613		*Pseudoalteromonas hodoensis*	Unjin port seawater	MA	37	7	5	+++	-	-	+
614		*Marinomonas arctica*	Aewol port seawater	MA	37	7	5	-	-	-	-
615		*Marinomonas arctica*	Aewol port seawater	MA	37	7	5	-	-	-	-
616		*Marinomonas arctica*	Aewol port seawater	MA	37	7	5	-	-	-	-
617		*Brevibacterium casei*	Aewol port seawater	MA	37	7	5	-	-	+	+
618		*Marinomonas arctica*	Aewol port seawater	MA	37	7	5	-	-	-	-
619		*Pseudomonas marincola*	Aewol port seawater	MA	37	7	5	-	-	-	-
620		*Pseudomonas sabulinigri*	Aewol port seawater	MA	37	7	5	-	-	-	-
621		*Aeromicrobium alkaliterrae*	Aewol port seawater	MA	37	7	5	-	-	-	-
622		*Aeromicrobium alkaliterrae*	Aewol port seawater	MA	37	7	5	-	-	-	-
623		*Psychrobacter sanguinis*	Daepo port seawater	MA	37	7	10	-	-	-	-
624		*Pseudoalteromonas issachenkonii*	Daepo port seawater	MA	37	7	10	-	-	-	+
625		*Roseivirga ehrenbergii*	Daepo port seawater	MA	37	7	5	-	-	-	-
626		*Roseivirga ehrenbergii*	Daepo port seawater	MA	37	7	5	-	-	-	-
627		*Pseudoalteromonas hodoensis*	Daepo port seawater	MA	37	7	5	+++	-	-	-
628		*Micrococcus yunnanensis*	Daepo port seawater	MA	37	7	5	-	-	-	+
629		*Pseudoalteromonas carrageenovora*	Daepo port seawater	MA	37	7	5	-	-	-	+++
630		*Pseudomonas marincola*	Daepo port seawater	MA	37	7	5	-	-	-	++
631	CP-39	*Bacillus firmus*	Daepo port seawater	MA	37	7	5	+++	-	-	+++
632		*Pseudoalteromonas issachenkonii*	Daepo port seawater	MA	37	7	5	-	-	-	++
633		*Pseudoalteromonas carrageenovora*	Daepo port seawater	MA	37	7	5	-	-	-	+
634		*Pseudoalteromonas carrageenovora*	Daepo port seawater	MA	37	7	5	++	-	-	++

635	CP-40	*Bacillus megaterium*	Yerae port seawater	MA	37	7	5	+++	-	-	+++
636		*Bacillus megaterium*	Yerae port seawater	MA	37	7	5	+++	-	-	-
637		*Pseudomonas salina*	Yerae port seawater	MA	37	7	10	-	-	-	-
638		*Sulfitobacter pontiacus*	Yerae port seawater	MA	37	7	10	-	-	-	-
639		*Brevibacterium luteolum*	Yerae port seawater	MA	37	7	10	-	-	-	-
640		*Bacillus drentensis*	Yerae port seawater	MA	37	7	10	+++	-	-	+
641		*Sulfitobacter dubius*	Yerae port seawater	MA	37	7	10	-	-	-	+
642		*Erythrobacter vulgaris*	Yerae port seawater	MA	37	7	10	-	-	-	-
643		*Erythrobacter vulgaris*	Yerae port seawater	MA	37	7	10	-	-	-	-
644		*Sulfitobacter pontiacus*	Yerae port seawater	MA	37	7	10	-	-	-	++
645		*Alteromonas addita*	Yerae port seawater	MA	37	7	10	+++	-	-	+
646		*Bacillus safensis*	Hwasun port seawater	MA	37	7	5	-	-	-	+++
647		*Cobetia marina*	Hwasun port seawater	MA	37	7	5	-	-	-	-
648		*Bacillus berkeleyi*	Hwasun port seawater	MA	37	7	10	-	-	-	-
649		*Bacillus aryabhattai*	Gangjeong port seawater	MA	37	7	5	+++	-	-	-
650		*Abyssicoccus albus*	Gangjeong port seawater	MA	37	7	5	-	-	-	-
651		*Abyssicoccus albus*	Gangjeong port seawater	MA	37	7	5	-	-	-	-
652		*Staphylococcus epidermidis*	Gangjeong port seawater	MA	37	7	5	-	-	-	++
653		*Staphylococcus hominis*	Gangjeong port seawater	MA	37	7	5	-	-	-	-
654		*Bacillus zhangzhouensis*	Gangjeong port seawater	MA	37	7	10	-	-	-	-
655		*Micrococcus yunnanensis*	Gangjeong port seawater	MA	37	7	10	-	-	-	++
656		*Staphylococcus hominis*	Gangjeong port seawater	MA	37	7	10	-	-	-	-
657		*Micrococcus yunnanensis*	Gangjeong port seawater	MA	37	7	10	-	-	-	+
658		*Pseudoalteromonas hodoensis*	Gangjeong port seawater	MA	37	7	10	+++	-	-	-
659		*Staphylococcus hominis*	Gangjeong port seawater	MA	37	7	10	+++	-	-	-
660		*Staphylococcus haemolyticus*	Gangjeong port seawater	MA	37	7	10	+++	-	-	-
661		*Staphylococcus petrasii subsp. Pragensis*	Gangjeong port seawater	MA	37	7	10	+++	-	-	++
662		*Micrococcus yunnanensis*	Moslpo port seawater	MA	37	7	5	-	-	-	-
663		*Staphylococcus hominis*	Moslpo port seawater	MA	37	7	5	-	-	-	-
664	CP-42	*Bacillus velezensis*	Moslpo port seawater	MA	37	7	10	+++	-	-	+++
665		*Bacillus vietnamensis*	Daepo port soil	MA	37	7	5	-	-	-	-
666	CP-43	*Bacillus hwajinpoensis*	Daepo port soil	MA	37	7	5	+++	-	-	+++
667	CP-44	*Bacillus hwajinpoensis*	Daepo port soil	MA	37	7	5	+++	-	-	+++
668		*Bacillus cereus*	Daepo port soil	MA	37	7	5	+++	-	-	+++
669		*Kocuria palustris*	Daepo port soil	MA	37	7	5	-	-	-	+
670		*Bacillus paralicheniformis*	Daepo port soil	MA	37	7	5	+	-	-	+++
671		*Kocuria palustris*	Daepo port soil	MA	37	7	5	-	-	-	+
672		*Bacillus hwajinpoensis*	Daepo port soil	MA	37	7	5	+++	-	-	+++
673		*Halobacillus salinus*	Daepo port soil	MA	37	7	5	-	-	-	++
674		*Bacillus aryabhattai*	Daepo port soil	MA	37	7	5	+++	-	-	-
675		*Bacillus subterraneus*	Daepo port soil	MA	37	7	10	+++	-	-	-
676		*Oceanobacillus iheyensis*	Daepo port soil	MA	37	7	10	+++	-	-	-

677	*Exiguobacterium marinum*	Daepo port soil	MA	37	7	10	+++	-	-	-
678	*Halobacillus trueperi*	Daepo port soil	MA	37	7	10	+++	-	-	-
679	*Bacillus horikoshii*	Daepo port soil	MA	37	7	10	+++	-	-	-
680	*Marinobacter sediminum*	Daepo port soil	MA	37	7	10	-	-	++	-
681	*Halobacillus trueperi*	Daepo port soil	MA	37	7	10	+++	-	-	-
682	*Bacillus paralicheniformis*	Daepo port soil	MA	37	7	10	-	-	-	-
683	*Halomonas venusta*	Hwasun port soil	MA	37	9	10	-	-	-	-
684	*Halomonas venusta*	Hwasun port soil	MA	37	9	10	-	-	-	-
685	*Exiguobacterium aestuarii*	Hwasun port soil	MA	37	9	5	+++	-	-	+++
686	*Vibrio alginolyticus*	Hwasun port soil	MA	37	9	5	+++	-	-	+++
687	*Exiguobacterium aestuarii*	Hwasun port soil	MA	37	9	5	+++	-	-	+++
688	*Halomonas venusta*	Hwasun port soil	MA	37	9	5	-	-	-	-
689	*Halomonas venusta*	Hwasun port soil	MA	37	9	5	-	-	-	-
690	*Vibrio sp. SRB-6-16*	Hwasun port soil	MA	37	9	5	-	-	-	-
691	*Vibrio alginolyticus*	Hwasun port soil	MA	37	9	5	+++	-	-	+++
692	*Vibrio alginolyticus*	Hwasun port soil	MA	37	9	5	+++	-	-	+++
693	*Vibrio alginolyticus*	Hwasun port soil	MA	37	9	5	-	-	-	-
694	*Planococcus maritimus*	Hwasun port soil	MA	37	7	10	-	-	-	-
695	*Planococcus maritimus*	Hwasun port soil	MA	37	7	10	-	-	-	-
696	*Planococcus maritimus*	Hwasun port soil	MA	37	7	10	-	-	-	-
697	*Cobetia amphilecti*	Hwasun port soil	MA	37	7	10	-	-	-	-
698	*Halomonas venusta*	Hwasun port soil	MA	37	7	10	-	-	-	+
699	*Cobetia marina*	Hwasun port soil	MA	37	7	10	-	-	-	-
700	*Cobetia marina*	Hwasun port soil	MA	37	7	5	-	-	-	-
701	*Planococcus citreus*	Hwasun port soil	MA	37	7	5	-	-	-	+++
702	*Cobetia marina*	Hwasun port soil	MA	37	7	5	+++	-	-	-
703	*Cobetia amphilecti*	Hwasun port soil	MA	37	7	5	+++	-	-	-
704	*Exiguobacterium oxidotolerans*	Hwasun port soil	MA	37	7	5	+++	-	-	+
705	*Planococcus citreus*	Hwasun port soil	MA	37	7	5	+++	-	-	+++
706	*Vibrio alginolyticus*	Hwasun port soil	MA	37	7	5	+++	-	-	+
707	*Oceanisphaera psychrotolerans*	Hwasun port soil	MA	37	7	5	+++	-	-	-
708	*Oceanisphaera psychrotolerans*	Hwasun port soil	MA	37	7	5	+++	-	-	-
709	*Exiguobacterium oxidotolerans*	Hwasun port soil	MA	37	7	5	+++	-	-	+
710	*Halomonas venusta*	Hwasun port soil	MA	37	9	5	-	-	-	-
711	*Halomonas zhanjiangensis*	Jeju sheep ranch soil	MA	37	7	10	+++	-	-	-
712	*Isoptericola halotolerans*	Jeju sheep ranch soil	MA	37	7	10	+++	-	-	++
713	*Staphylococcus equorum*	Jeju sheep ranch soil	MA	37	7	10	-	-	-	+++
714	*Staphylococcus equorum*	Jeju sheep ranch soil	MA	37	7	10	-	-	-	++
715	*Arthrobacter gandavensis*	Jeju sheep ranch soil	MA	37	7	5	-	-	+	-
716	*Pseudomonas xanthomarina*	Jeju sheep ranch soil	MA	37	7	5	-	-	-	-
717	*Microbacterium profundi*	Jeju sheep ranch soil	MA	37	7	5	+++	-	-	+
718	*Bacillus aryabhattai*	Jeju sheep ranch soil	MA	37	7	5	+++	-	-	-
719	*Bacillus aryabhattai*	Jeju sheep ranch soil	MA	37	7	5	+++	-	-	+

720		*Staphylococcus equorum*	Jeju sheep ranch soil	MA	37	7	5	+++	-	-	+
721		*Paracoccus alcaliphilus*	Jeju sheep ranch soil	MA	37	7	5	-	-	-	+++
722		*Sanguibacter antarcticus*	Jeju sheep ranch soil	MA	37	7	5	+++	-	-	+++
723		*Planomicrobium okeanokoites*	Jeju sheep ranch soil	MA	37	7	5	-	-	-	+++
724		*Sanguibacter antarcticus*	Jeju sheep ranch soil	MA	37	7	5	+++	-	-	+++
725		*Arthrobacter luteolus*	Jeju sheep ranch soil	MA	37	7	10	-	-	-	-
726		*Corynebacterium faecale*	Jeju sheep ranch soil	MA	37	7	5	-	-	-	+
727		*Planomicrobium chinense*	Jeju sheep ranch soil	MA	37	7	5	-	-	-	+++
728		*Staphylococcus epidermidis*	Jeju sheep ranch soil	MA	37	7	5	-	-	-	++
729		*Corynebacterium faecale*	Jeju sheep ranch soil	MA	37	7	5	-	-	-	+
730		*Planomicrobium chinense*	Jeju sheep ranch soil	MA	37	7	5	-	-	-	+++
731		*Staphylococcus epidermidis*	Jeju sheep ranch soil	MA	37	7	5	-	-	-	++
732		*Demequina activiva*	Jeju sheep ranch soil	MA	37	7	5	-	-	-	-
733		*Halomonas zhanjiangensis*	Jeju sheep ranch soil	MA	37	9	5	-	-	-	+
734		*Agrococcus casei*	Jeju sheep ranch soil	MA	37	9	5	-	-	-	++
735		*Agrococcus casei*	Jeju sheep ranch soil	MA	37	9	5	-	-	-	++
736		*Pseudomonas bauzanensis*	Jeju sheep ranch soil	MA	37	9	5	-	-	-	-
737		*Jonesia luteola*	Jeju sheep ranch soil	MA	37	9	5	+++	-	-	+
738		*Jonesia quinghaiensis*	Jeju sheep ranch soil	MA	37	9	5	+++	-	+	+++
739		*Aeromicrobium halocynthiae*	Jeju sheep ranch soil	MA	37	9	5	-	-	-	+++
740		*Halomonas alkaliantarctica*	Jeju sheep ranch soil	MA	37	9	5	+++	-	+	+
741		*Halomonas alkaliantarctica*	Jeju sheep ranch soil	MA	37	9	5	+++	-	-	-
742		*Jonesia luteola*	Jeju sheep ranch soil	MA	37	9	5	++	-	-	+
743		*Pseudomonas bauzanensis*	Jeju sheep ranch soil	MA	37	9	5	-	-	-	-
744		*Jonesia quinghaiensis*	Jeju sheep ranch soil	MA	37	9	5	+++	-	-	-
745		*Halomonas zhanjiangensis*	Jeju sheep ranch soil	MA	37	9	5	-	-	-	-
746		*Jonesia luteola*	Jeju sheep ranch soil	MA	37	9	5	+++	-	-	+
747		*Arthrobacter gandavensis*	Jeju sheep ranch soil	MA	37	7	5	-	-	+	-
748		*Aeromicrobium halocynthiae*	Jeju sheep ranch soil	MA	37	9	5	-	-	-	+++
749		*Bacillus subtilis*	Greenfield ranch soil	MA	50	7	10	+++	-	-	++
750		*Bacillus paralicheniformis*	Greenfield ranch soil	MA	50	7	10	+	-	-	+
751		*Bacillus paralicheniformis*	Greenfield ranch soil	MA	50	7	5	-	-	-	+++
752	CP-34	*Bacillus paralicheniformis*	Greenfield ranch soil	MA	50	7	5	+++	-	-	+++
753		*Bacillus thermolactis*	Greenfield ranch soil	MA	50	7	5	-	-	-	-
754		*Bacillus kokeshiiformis*	Greenfield ranch soil	MA	50	7	5	-	-	-	-
755		*Bacillus thermolactis*	Greenfield ranch soil	MA	50	7	5	+	-	-	+
756		*Bacillus thermolactis*	Greenfield ranch soil	MA	50	7	5	-	-	-	++
757		*Ruania albidiflava*	Greenfield ranch soil	MA	37	7	5	-	-	-	++
758		*Microbacterium suwonense*	Greenfield ranch soil	MA	37	7	5	+++	-	-	-
759		*Staphylococcus warneri*	Greenfield ranch soil	MA	37	7	5	-	-	-	-
760		*Sinorhizobium saheli*	Greenfield ranch soil	MA	37	7	5	-	-	-	-
761		*Bacillus paralicheniformis*	Greenfield ranch soil	MA	37	7	5	+++	-	-	+++
762		*Hephaestia caeni*	Greenfield ranch soil	MA	37	7	5	-	-	-	-

763		*Luteimonas lutimaris*	Greenfield ranch soil	MA	37	7	5	-	-	-	++
764		*Sinorhizobium saheli*	Greenfield ranch soil	MA	37	7	5	-	-	-	-
765		*Luteimonas lutimaris*	Greenfield ranch soil	MA	37	7	5	-	-	-	++
766		*Luteimonas lutimaris*	Greenfield ranch soil	MA	37	7	5	-	-	-	++
767		*Hephaestia caeni*	Greenfield ranch soil	MA	37	7	5	-	-	-	-
768		*Galbibacter marinus*	Greenfield ranch soil	MA	37	7	5	++	-	-	-
769		*Sinorhizobium saheli*	Greenfield ranch soil	MA	37	7	5	-	-	-	-
770		*Sinorhizobium saheli*	Greenfield ranch soil	MA	37	7	5	-	-	-	+
771		*Sinorhizobium saheli*	Greenfield ranch soil	MA	37	7	5	-	-	-	-
772		*Arthrobacter humicola*	Greenfield ranch soil	MA	37	7	5	-	-	-	+
773		*Arthrobacter oryzae*	Greenfield ranch soil	MA	37	7	5	-	-	-	++
774		*Ruania albidiflava*	Greenfield ranch soil	MA	37	7	10	-	-	-	+
775		*Bacillus paralicheniformis*	Greenfield ranch soil	MA	37	7	10	+++	-	-	+
776		*Bacillus subtilis*	Greenfield ranch soil	MA	37	7	10	+++	-	-	+
777		*Bacillus subtilis*	Greenfield ranch soil	MA	37	7	10	+++	-	-	+
778		*Arthrobacter humicola*	Greenfield ranch soil	MA	37	7	10	-	-	-	+
779		*Ruania albidiflava*	Greenfield ranch soil	MA	37	7	5	-	-	-	++
780		*Curtobacterium oceanosedimentum*	Greenfield ranch soil	MA	37	7	5	-	-	-	+++
781		*Pusillimonas ginsengisoli*	Greenfield ranch soil	MA	37	7	5	-	-	-	-
782		*Curtobacterium oceanosedimentum*	Greenfield ranch soil	MA	37	7	5	-	-	-	+++
783		*Nocardia vermiculata*	Greenfield ranch soil	MA	37	7	5	-	-	-	-
784	CP-35	*Bacillus subtilis*	Greenfield ranch soil	MA	37	7	5	+++	-	+	+++
785		*Bacillus paralicheniformis*	Greenfield ranch soil	MA	37	7	5	++	-	-	+++
786		*Pusillimonas ginsengisoli*	Greenfield ranch soil	MA	37	7	5	-	-	-	-
787		*Nocardia vermiculata*	Greenfield ranch soil	MA	37	7	5	-	-	-	-
788		*Curtobacterium luteum*	Greenfield ranch soil	MA	37	7	5	-	-	-	+
789		*Bacillus subtilis*	Greenfield ranch soil	MA	37	7	5	+++	-	-	+++
790		*Galbibacter marinus*	Greenfield ranch soil	MA	37	7	5	-	-	-	-
791		*Microbacterium suwonense*	Greenfield ranch soil	MA	37	7	5	-	-	-	+
792		*Pusillimonas ginsengisoli*	Greenfield ranch soil	MA	37	7	5	-	-	-	-
793		*Pusillimonas ginsengisoli*	Greenfield ranch soil	MA	37	7	5	-	-	-	-
794		*Pusillimonas ginsengisoli*	Greenfield ranch soil	MA	37	7	5	-	-	-	-
795		*Curtobacterium luteum*	Greenfield ranch soil	MA	37	7	5	-	-	-	+
796		*Pantoea ananatis*	Greenfield ranch soil	MA	37	4	5	+++	-	-	+++
797		*Pantoea ananatis*	Greenfield ranch soil	MA	37	4	5	-	-	++	+++
798		*Bacillus paralicheniformis*	Greenfield ranch soil	MA	37	7	5	-	-	-	+++
799		*Bacillus firmus*	Greenfield ranch soil	MA	37	7	5	-	-	-	+++
800		*Streptomyces avermitilis*	Greenfield ranch soil	MA	37	7	5	-	-	-	-
801		*Bacillus firmus*	Greenfield ranch soil	MA	37	7	5	-	-	-	+++
802		*Isoptericola variabilis*	Greenfield ranch soil	MA	37	7	5	+++	-	-	+
803		*Arthrobacter ureafaciens*	Greenfield ranch soil	MA	37	7	5	-	-	-	+

804		*Isoptericola variabilis*	Greenfield ranch soil	MA	37	7	5	+++	-	-	+
805		*Bacillus thermolactis*	Greenfield ranch soil	MA	50	7	5	-	-	-	++
806		*Bacillus paralicheniformis*	Greenfield ranch soil	MA	50	7	5	+++	-	-	+++
807		*Bacillus paralicheniformis*	Greenfield ranch soil	MA	50	7	5	+++	-	-	+++
808		*Bacillus timonensis*	Greenfield ranch soil	MA	37	7	10	+++	-	-	-
809		*Bacillus paralicheniformis*	Greenfield ranch soil	MA	37	7	10	+++	-	-	-
810		*Bacillus oryzaecorticis*	Greenfield ranch soil	MA	37	7	10	+++	-	-	-
811		*Bacillus paralicheniformis*	Greenfield ranch soil	MA	37	7	10	+++	-	-	-
812		*Bacillus paralicheniformis*	Greenfield ranch soil	MA	50	7	10	+++	-	-	++
813		*Bacillus paralicheniformis*	Ole market hairtail fermented food	MA	37	7	10	+++	-	-	-
814		*Bacillus subtilis*	Ole market hairtail fermented food	MA	37	7	10	+++	-	-	-
815		*Bacillus aerius*	Ole market hairtail fermented food	MA	37	7	10	+++	-	-	-
816		*Bacillus subtilis*	Ole market hairtail fermented food	MA	37	7	10	+++	-	-	-
817		*Bacillus paralicheniformis*	Ole market hairtail fermented food	MA	37	7	10	+++	-	-	++
818		*Bacillus aerius*	Ole market hairtail fermented food	MA	37	7	10	+++	-	-	-
819	CP-36	*Bacillus lehensis*	Ole market hairtail fermented food	MA	37	7	5	+++	-	-	+++
820		*Bacillus subtilis*	Ole market hairtail fermented food	MA	37	7	5	+++	-	-	+++
821		*Bacillus aerius*	Ole market hairtail fermented food	MA	37	7	5	-	-	-	+++
822		*Bacillus aerius*	Ole market hairtail fermented food	MA	37	7	5	-	-	-	+++
823		*Bacillus subtilis*	Ole market hairtail fermented food	MA	37	7	5	+++	-	-	+
824		*Bacillus altitudinis*	Ole market hairtail fermented food	MA	37	7	5	+++	-	-	-
825		*Bacillus aerius*	Ole market hairtail fermented food	MA	37	7	5	+++	-	-	-
826		*Bacillus aerius*	Ole market hairtail fermented food	MA	37	7	5	+++	-	-	+
827		*Bacillus aerius*	Ole market hairtail fermented food	MA	37	7	5	-	-	-	-
828		*Bacillus aerius*	Ole market hairtail fermented food	MA	37	7	5	-	-	-	-
829		*Bacillus paralicheniformis*	Ole market hairtail fermented food	MA	37	7	5	-	-	-	-
830		*Bacillus velezensis*	Ole market hairtail fermented food	MA	50	7	5	+++	-	-	+++
831		*Bacillus paralicheniformis*	Ole market hairtail fermented food	MA	50	7	5	+++	-	-	+++
832	CP-37	*Bacillus subtilis*	Ole market hairtail fermented food	MA	50	7	5	+++	-	-	+++
833		*Bacillus aerius*	Ole market hairtail fermented food	MA	50	7	5	-	-	-	+
834		*Bacillus aerius*	Ole market hairtail fermented food	MA	50	7	5	-	-	-	+++
835		*Bacillus stratosphericus*	Ole market hairtail fermented food	MA	50	7	10	-	-	-	-
836		*Bacillus subtilis*	Ole market hairtail fermented food	MA	50	7	10	+++	-	-	+++
837		*Bacillus zhangzhouensis*	Ole market hairtail fermented food	MA	50	7	10	-	-	-	+++
838		*Bacillus stratosphericus*	Ole market hairtail fermented food	MA	37	7	5	-	-	-	-
839		*Bacillus altitudinis*	Ole market hairtail fermented food	MA	37	7	5	+++	-	-	-
840		*Staphylococcus capitis*	Morning smile ranch soil	MA	37	7	5	+++	-	+	+
841		*Bacillus velezensis*	Morning smile ranch soil	MA	37	7	5	-	-	+	+
842		*Massilia varians*	Morning smile ranch soil	MA	37	4	10	+++	-	-	-
843		*Massilia varians*	Morning smile ranch soil	MA	37	4	10	+++	-	-	-
844		*Micrococcus yunnanensis*	Morning smile ranch soil	MA	37	7	10	+++	-	-	-
845		*Paenibacillus campinasensis*	peatmoss (Silla univ. field)	MA	37	7	3	+++	-	-	-
846		*Streptomyces pulveraceus*	peatmoss (Silla univ. field)	MA	37	7	3	+++	-	-	-

847		*Paenibacillus tundrae*	peatmoss (Silla univ. field)	MA	37	7	3	+++	-	-	-
848		*Bacillus zhangzhouensis*	peatmoss (Silla univ. field)	MA	37	7	3	-	-	-	-
849		*Paenibacillus tundrae*	peatmoss (Silla univ. field)	MA	37	7	3	+++	-	-	-
850		*Streptomyces griseolus*	peatmoss (Silla univ. field)	MA	37	7	3	+++	-	-	-
851		*Bacillus mycoides*	peatmoss (Silla univ. field)	MA	37	7	3	+++	-	-	-
852		*Streptomyces halstedii*	peatmoss (Silla univ. field)	MA	37	7	3	+++	-	-	-
853		*Streptomyces costaricanus*	peatmoss (Silla univ. field)	MA	37	7	3	++	-	-	-
854		*Bacillus mycoides*	peatmoss (Silla univ. field)	MA	37	7	3	+++	-	-	-
855		*Bacillus mycoides*	peatmoss (Silla univ. field)	MA	37	7	3	+++	-	-	-
856		*Bacillus siralis*	peatmoss (Silla univ. field)	MA	50	7	3	-	-	-	+++
857		*Bacillus mycoides*	peatmoss (Silla univ. field)	MA	37	7	3	++	-	-	-
858		*Paenibacillus amylolyticus*	peatmoss (Silla univ. field)	MA	37	7	3	-	-	-	-
859		*Bacillus mycoides*	peatmoss (Silla univ. field)	MA	37	7	3	-	-	-	-
860		*Domibacillus robiginosus*	peatmoss (Silla univ. field)	MA	37	7	3	-	-	-	-
861		*Bacillus mycoides*	peatmoss (Silla univ. field)	MA	37	7	3	-	-	-	-
862		*Bacillus okhensis*	peatmoss (Silla univ. field)	MA	37	7	3	-	-	-	-
863		*Paenibacillus tundrae*	peatmoss (Silla univ. field)	MA	37	7	3	+++	-	-	-
864		*Paenibacillus tundrae*	peatmoss (Silla univ. field)	MA	37	7	3	+++	-	-	-
865		*Bacillus muralis*	peatmoss (Silla univ. field)	MA	37	7	3	-	-	-	-
866		*Bacillus wiedmannii*	peatmoss (Silla univ. field)	MA	37	7	3	+++	-	-	-
867		*Paenibacillus tundrae*	peatmoss (Silla univ. field)	MA	37	7	3	+++	-	-	-
868		*Paenibacillus tundrae*	peatmoss (Silla univ. field)	MA	37	7	3	+++	-	-	-
869		*Streptomyces fulvissimus*	peatmoss (Silla univ. field)	MA	37	7	3	-	-	-	-
870		*Paenibacillus tundrae*	peatmoss (Silla univ. field)	MA	37	7	3	+++	-	-	+
871		*Paenibacillus tundrae*	peatmoss (Silla univ. field)	MA	37	7	3	+++	-	-	-
872		*Bacillus weihenstephanensis*	peatmoss (Silla univ. field)	MA	37	7	3	+++	-	-	-
873		*Bacillus halmapalus*	peatmoss (Silla univ. field)	MA	37	7	3	+++	-	-	-
874		*Bacillus mycoides*	peatmoss (Silla univ. field)	MA	37	7	3	+++	-	-	-
875		*Bacillus halmapalus*	peatmoss (Silla univ. field)	MA	37	7	3	+++	-	-	-
876		*Bacillus lindianensis*	peatmoss (Silla univ. field)	MA	37	7	3	-	-	-	-
877		*Bacillus okhensis*	peatmoss (Silla univ. field)	MA	37	7	3	-	-	-	-
878		*Bacillus halmapalus*	peatmoss (Silla univ. field)	MA	37	7	3	++	-	-	-
879		*Streptomyces sampsonii*	peatmoss (Silla univ. field)	MA	37	7	3	+++	-	-	-
880		*Bacillus weihenstephanensis*	peatmoss (Silla univ. field)	MA	37	7	3	+++	-	-	-
881	CP-38	*Bacillus thermoamylovorans*	peatmoss (Silla univ. field)	MA	50	7	3	+++	-	-	+++
882		*Thermobacillus composti*	peatmoss (Silla univ. field)	MA	50	7	3	+++	-	-	+++
883		*Bacillus siralis*	peatmoss (Silla univ. field)	MA	50	7	3	-	-	-	+++
884		*Streptomyces griseolus*	peatmoss (Silla univ. field)	MA	37	7	3	+++	-	-	-
885		*Paenibacillus cineris*	peatmoss (Silla univ. field)	MA	37	7	3	+	-	-	-
886		*Paenibacillus cineris*	peatmoss (Silla univ. field)	MA	37	7	3	+	-	-	-
887		*Lysinibacillus fusiformis*	peatmoss (Silla univ. field)	MA	37	7	3	-	-	-	-
888		*Virgibacillus halophilus*	peatmoss (Silla univ. field)	MA	37	7	3	-	-	-	-
889		*Bacillus wiedmannii*	peatmoss (Silla univ. field)	MA	37	7	3	+++	-	-	-

890	*Bacillus mycoides*	peatmoss (Silla univ. field)	MA	37	7	3	+++	-	-	-
891	*Bacillus mycoides*	peatmoss (Silla univ. field)	MA	37	7	3	+++	-	-	-
892	*Bacillus mycoides*	peatmoss (Silla univ. field)	MA	37	7	3	+++	-	-	-
893	*Lysinibacillus fusiformis*	peatmoss (Silla univ. field)	MA	37	7	3	-	-	-	-
894	*Lysinibacillus fusiformis*	peatmoss (Silla univ. field)	MA	37	7	3	-	-	-	-
895	*Streptomyces griseolus*	peatmoss (Silla univ. field)	MA	37	7	3	+++	-	-	-
896	*Bacillus firmus*	peatmoss (Silla univ. field)	MA	37	7	3	-	-	-	-
897	*Lysinibacillus fusiformis*	peatmoss (Silla univ. field)	MA	37	7	3	-	-	-	-
898	*Lysinibacillus fusiformis*	peatmoss (Silla univ. field)	MA	37	7	3	-	-	-	-
899	*Paenibacillus cookii*	peatmoss (Silla univ. field)	MA	50	7	3	+++	-	-	-
900	*Paenibacillus phoenicis*	peatmoss (Silla univ. field)	MA	50	7	3	+++	-	-	-
901	*Brevibacillus borstelensis*	peatmoss (Silla univ. field)	MA	37	7	3	-	-	-	-
902	*Bacillus halmapalus*	peatmoss (Silla univ. field)	MA	37	7	3	+++	-	-	-
903	*Brevibacillus thermoruber*	peatmoss (Silla univ. field)	MA	50	7	3	-	-	-	+++
904	*Aeribacillus pallidus*	peatmoss (Silla univ. field)	MA	50	7	3	-	-	-	-
905	*Aeribacillus pallidus*	peatmoss (Silla univ. field)	MA	50	7	3	-	-	-	-
906	*Aneurinibacillus danicus*	peatmoss (Silla univ. field)	MA	50	7	3	-	-	-	-
907	*Brevibacillus thermoruber*	peatmoss (Silla univ. field)	MA	50	7	3	+++	-	-	-
908	*Brevibacillus thermoruber*	peatmoss (Silla univ. field)	MA	50	7	3	+++	-	-	+++
909	*Aeribacillus pallidus*	peatmoss (Silla univ. field)	MA	50	7	3	-	-	-	-
910	*Bacillus methanolicus*	peatmoss (Silla univ. field)	MA	50	7	3	-	-	-	-
911	*Brevibacillus thermoruber*	peatmoss (Silla univ. field)	MA	50	7	3	+++	-	-	+++
912	*Brevibacillus thermoruber*	peatmoss (Silla univ. field)	MA	50	7	3	+++	-	-	+++
913	*Aeribacillus pallidus*	peatmoss (Silla univ. field)	MA	50	7	3	-	-	-	-
914	*Paenibacillus naphthalenovorans*	peatmoss (Silla univ. field)	MA	50	7	3	-	-	-	-
915	*Aeribacillus pallidus*	peatmoss (Silla univ. field)	MA	50	7	3	-	-	-	-
916	*Bacillus vietnamensis*	Sinan taepyeong salts	MA	37	6	15	+++	-	-	-
917	*Halobacillus trueperi*	Sinan taepyeong salts	MA	37	8	15	+++	-	-	-
918	*Halobacillus trueperi*	Sinan taepyeong salts	MA	37	6	15	+++	-	-	-
919	*Pontibacillus salipaludis*	Sinan taepyeong salts	MA	37	6	15	+++	-	-	-
920	*Halobacillus hunanensis*	Sinan taepyeong salts	MA	37	6	15	+++	-	-	-
921	*Pontibacillus salipaludis*	Sinan taepyeong salts	MA	37	6	15	+++	-	-	-
922	*Bacillus aquimaris*	Sinan taepyeong salts	MA	37	6	15	+++	-	-	-
923	*Halobacillus trueperi*	Sinan taepyeong salts	MA	37	6	15	+++	-	-	-
924	*Halobacillus campisalis*	Sinan taepyeong salts	MA	37	6	15	+++	-	-	-
925	*Halobacillus mangrovi*	Sinan taepyeong salts	MA	37	6	15	+++	-	-	-
926	*Halobacillus trueperi*	Sinan taepyeong salts	MA	37	6	15	+++	-	-	-
927	*Halobacillus campisalis*	Sinan taepyeong salts	MA	37	6	15	+++	-	-	-
928	*Bacillus aquimaris*	Sinan taepyeong salts	MA	37	6	15	+++	-	-	-
929	*Bacillus hwajinpoensis*	Sinan taepyeong salts	MA	37	6	15	+	-	-	-
930	*Halobacillus trueperi*	Sinan taepyeong salts	MA	37	6	15	+++	-	-	-
931	*Halobacillus mangrovi*	Sinan taepyeong salts	MA	37	6	15	+++	-	-	-
932	*Halobacillus trueperi*	Sinan taepyeong salts	MA	37	6	15	+++	-	-	-

933	*Thalassobacillus devorans*	Sinan taepyeong salts	MA	37	6	15	+++	-	+++	-
934	*Halobacillus trueperi*	Sinan taepyeong salts	MA	37	6	15	+++	-	-	+++
935	*Halobacillus halophilus*	Sinan taepyeong salts	MA	37	6	15	+++	-	-	-
936	*Pontibacillus salipaludis*	Sinan taepyeong salts	MA	37	6	15	++	-	-	+++
937	*Bacillus aquimaris*	Sinan taepyeong salts	MA	37	6	15	+++	-	-	-
938	*Halobacillus halophilus*	Sinan taepyeong salts	MA	37	6	15	+++	-	-	-
939	*Halobacillus trueperi*	Sinan taepyeong salts	MA	37	6	15	+++	-	-	-
940	*Halobacillus halophilus*	Sinan taepyeong salts	MA	37	8	15	+++	-	-	+++
941	*Thalassobacillus devorans*	Sinan taepyeong salts	MA	37	8	15	+++	-	-	-
942	*Halobacillus campisalis*	Sinan taepyeong salts	MA	37	8	15	+++	-	-	-
943	*Halobacillus halophilus*	Sinan taepyeong salts	MA	37	8	15	+++	-	-	-
944	*Thalassobacillus devorans*	Sinan taepyeong salts	MA	37	8	15	-	-	-	-
945	*Halobacillus hunanensis*	Sinan taepyeong salts	MA	37	8	15	+++	-	-	-
946	*Halobacillus trueperi*	Sinan taepyeong salts	MA	37	8	15	+++	-	-	-
947	*Bacillus aquimaris*	Sinan taepyeong salts	MA	37	8	15	+++	-	-	-
948	*Halobacillus trueperi*	Sinan taepyeong salts	MA	37	8	15	+++	-	-	-
949	*Halobacillus trueperi*	Sinan taepyeong salts	MA	37	8	15	+++	-	-	-
950	*Bacillus hwajinpoensis*	Sinan taepyeong salts	MA	37	8	15	+++	-	-	-
951	*Pontibacillus salipaludis*	Sinan taepyeong salts	MA	37	8	15	+++	-	-	-
952	*Halobacillus campisalis*	Sinan taepyeong salts	MA	37	8	15	+++	-	-	-
953	*Halobacillus trueperi*	Sinan taepyeong salts	MA	37	8	15	+++	-	-	-
954	*Halobacillus halophilus*	Sinan taepyeong salts	MA	37	8	15	+++	-	-	-
955	*Thalassobacillus devorans*	Sinan taepyeong salts	MA	37	8	15	+++	-	-	-
956	*Halobacillus halophilus*	Sinan taepyeong salts	MA	37	8	15	+++	-	-	-
957	*Halobacillus mangrovi*	Sinan taepyeong salts	MA	37	6	15	+++	-	-	-
958	*Halobacillus halophilus*	Sinan taepyeong salts	MA	37	6	15	++	-	-	++
959	*Halobacillus halophilus*	Sinan taepyeong salts	MA	37	6	15	++	-	-	-
960	*Halobacillus halophilus*	Sinan taepyeong salts	MA	37	6	15	+++	-	++	-
961	*Bacillus aquimaris*	Sinan taepyeong salts	MA	37	6	15	+++	-	-	+
962	*Halobacillus trueperi*	Sinan taepyeong salts	MA	37	6	15	+++	-	-	++
963	*Halobacillus halophilus*	Sinan taepyeong salts	MA	37	6	15	+++	-	-	-
964	*Halobacillus halophilus*	Sinan taepyeong salts	MA	37	6	15	+++	-	-	-
965	*Bacillus aquimaris*	Sinan taepyeong salts	MA	37	6	15	+++	-	+	+
966	*Pontibacillus salipaludis*	Sinan taepyeong salts	MA	37	6	15	+++	-	-	++
967	*Halobacillus hunanensis*	Sinan taepyeong salts	MA	37	6	15	+++	-	-	++
968	*Thalassobacillus devorans*	Sinan taepyeong salts	MA	37	6	15	+++	-	-	-
969	*Halobacillus halophilus*	Sinan taepyeong salts	MA	37	6	15	+++	-	-	++
970	*Halobacillus mangrovi*	Sinan taepyeong salts	MA	37	6	15	+++	-	-	-
971	*Halobacillus trueperi*	Sinan taepyeong salts	MA	37	6	15	+++	-	-	-
972	*Halobacillus trueperi*	Sinan taepyeong salts	MA	37	6	15	+++	-	-	-
973	*Bacillus aquimaris*	Sinan taepyeong salts	MA	37	6	15	+++	-	-	-
974	*Halobacillus campisalis*	Sinan taepyeong salts	MA	37	6	15	+++	-	-	-
975	*Halobacillus campisalis*	Sinan taepyeong salts	MA	37	6	15	+++	-	-	-

976		*Halobacillus trueperi*	Sinan taepyeong salts	MA	37	8	15	+++	-	-	-
977		*Halobacillus halophilus*	Sinan taepyeong salts	MA	37	8	15	+++	-	-	-
978		*Halobacillus trueperi*	Sinan taepyeong salts	MA	37	8	15	+++	-	-	-
979		*Halobacillus halophilus*	Sinan taepyeong salts	MA	37	8	15	+++	-	-	-
980		*Bacillus aquimaris*	Sinan taepyeong salts	MA	37	8	15	+++	-	-	-
981		*Halobacillus mangrovi*	Sinan taepyeong salts	MA	37	8	15	+++	-	-	-
982		*Pontibacillus salipaludis*	Sinan taepyeong salts	MA	37	8	15	+++	-	-	-
983	CP-41	*Bacillus aquimaris*	Sinan taepyeong salts	MA	37	8	15	+++	-	++	+++
984		*Halobacillus halophilus*	Sinan taepyeong salts	MA	37	8	15	+++	-	-	-
985		*Halobacillus trueperi*	Sinan taepyeong salts	MA	37	8	15	+++	-	-	-
986		*Pontibacillus salipaludis*	Sinan taepyeong salts	MA	37	8	15	+++	-	+++	-
987		*Halobacillus campisalis*	Sinan taepyeong salts	MA	37	8	15	+++	-	-	-
988		*Halobacillus halophilus*	Sinan taepyeong salts	MA	37	8	15	+++	-	-	-
989		*Halobacillus hunanensis*	Sinan taepyeong salts	MA	37	8	15	++	-	-	-
990		*Halobacillus trueperi*	Sinan taepyeong salts	MA	37	8	15	+++	-	-	+
991		*Halobacillus campisalis*	Sinan taepyeong salts	MA	37	8	15	+++	-	-	++
992		*Halobacillus trueperi*	Sinan taepyeong salts	MA	37	8	15	+++	-	-	-
993		*Halobacillus halophilus*	Sinan taepyeong salts	MA	37	8	15	+++	-	-	-
994		*Halobacillus trueperi*	Gomso saltpan	MA	37	6	15	+++	-	-	-
995		*Halobacillus trueperi*	Gomso saltpan	MA	37	6	15	+++	-	+	-
996		*Halobacillus trueperi*	Gomso saltpan	MA	37	6	15	+++	-	-	++
997		*Halobacillus locisalis*	Gomso saltpan	MA	37	6	15	+++	-	-	-
998		*Pontibacillus salipaludis*	Gomso saltpan	MA	37	6	15	-	-	++	-
999		*Halobacillus locisalis*	Gomso saltpan	MA	37	6	15	-	-	-	-
1000		*Pontibacillus yanchengensis*	Gomso saltpan	MA	37	6	15	-	-	-	-
1001		*Halobacillus trueperi*	Gomso saltpan	MA	37	6	15	+++	-	-	-
1002		*Virgibacillus albus*	Gomso saltpan	MA	37	6	15	-	-	-	-
1003		*Halobacillus locisalis*	Gomso saltpan	MA	37	6	15	-	-	-	-
1004		*Halobacillus trueperi*	Gomso saltpan	MA	37	6	15	+++	-	-	-
1005		*Thalassobacillus hwangdonensis*	Gomso saltpan	MA	37	6	15	-	-	-	-
1006		*Thalassobacillus hwangdonensis*	Gomso saltpan	MA	37	6	15	-	-	-	-
1007		*Pontibacillus yanchengensis*	Gomso saltpan	MA	37	6	15	-	-	-	-
1008		*Virgibacillus albus*	Gomso saltpan	MA	37	6	15	-	-	-	-
1009		*Virgibacillus dokdonensis*	Gomso saltpan	MA	37	8	15	-	-	-	-
1010		*Halobacillus trueperi*	Gomso saltpan	MA	37	8	15	+++	-	++	-
1011		*Pontibacillus marinus*	Gomso saltpan	MA	37	8	15	-	-	-	-
1012		*Halobacillus trueperi*	Gomso saltpan	MA	37	8	15	+++	-	-	-
1013		*Bacillus hwajinpoensis*	Gomso saltpan	MA	37	8	15	+++	-	-	-
1014		*Filobacillus milosensis*	Gomso saltpan	MA	37	8	15	-	-	-	-
1015		*Halobacillus halophilus*	Gomso saltpan	MA	37	8	15	-	-	-	-
1016		*Virgibacillus halodenitrificans*	Gomso saltpan	MA	37	8	15	-	-	-	+
1017		*Halobacillus locisalis*	Gomso saltpan	MA	37	8	15	+++	-	-	-
1018		*Oceanobacillus picturae*	lake salt	MA	37	6	15	+++	-	-	+

1019		*Oceanobacillus picturae*	lake salt	MA	37	6	15	-	-	-	+
1020		*Oceanobacillus picturae*	lake salt	MA	37	6	15	+++	-	+++	+
1021		*Virgibacillus senegalensis*	lake salt	MA	37	6	15	-	-	+++	-
1022		*Oceanobacillus picturae*	lake salt	MA	37	6	15	+++	-	+++	+
1023		*Oceanobacillus picturae*	lake salt	MA	37	6	15	+++	-	+++	-
1024		*Virgibacillus senegalensis*	lake salt	MA	37	6	15	-	-	+++	-
1025		*Oceanobacillus picturae*	lake salt	MA	37	8	15	+++	-	-	+
1026		*Oceanobacillus picturae*	lake salt	MA	37	8	15	-	-	-	-
1027		*Tenuibacillus halotolerans*	lake salt	MA	37	8	15	-	-	-	-
1028		*Tenuibacillus halotolerans*	lake salt	MA	37	8	15	+++	-	-	-
1029		*Oceanobacillus picturae*	lake salt	MA	37	8	15	+++	-	-	-
1030		*Oceanobacillus picturae*	lake salt	MA	37	8	15	+++	-	-	-
1031		*Oceanobacillus picturae*	lake salt	MA	37	8	15	+++	-	-	-
1032		*Virgibacillus carmonensis*	volcanic salts	MA	37	6	15	-	-	+	++
1033		*Virgibacillus carmonensis*	volcanic salts	MA	37	6	15	-	-	+	++
1034		*Oceanobacillus picturae*	volcanic salts	MA	37	6	15	+++	-	-	+
1035		*Oceanobacillus picturae*	volcanic salts	MA	37	6	15	-	-	-	+++
1036		*Halobacillus naozhouensis*	volcanic salts	MA	37	6	15	-	-	-	+
1037		*Halobacillus hunanensis*	volcanic salts	MA	37	6	15	-	-	-	-
1038		*Halobacillus hunanensis*	volcanic salts	MA	37	6	15	-	-	-	-
1039		*Halobacillus hunanensis*	volcanic salts	MA	37	6	15	-	-	-	-
1040		*Halobacillus hunanensis*	volcanic salts	MA	37	6	15	+++	-	-	-
1041		*Halobacillus hunanensis*	volcanic salts	MA	37	6	15	+++	-	-	-
1042		*Halobacillus naozhouensis*	volcanic salts	MA	37	6	15	-	-	-	+
1043		*Halobacillus locisalis*	volcanic salts	MA	37	8	15	-	-	-	-
1044		*Oceanobacillus picturae*	volcanic salts	MA	37	8	15	+++	-	++	+
1045		*Oceanobacillus iheyensis*	volcanic salts	MA	37	8	15	-	-	-	-
1046		*Oceanobacillus picturae*	volcanic salts	MA	37	8	15	-	-	-	-
1047		*Oceanobacillus picturae*	volcanic salts	MA	37	8	15	+++	-	++	-
1048		*Oceanobacillus picturae*	volcanic salts	MA	37	8	15	-	-	+	++
1049		*Halobacillus locisalis*	volcanic salts	MA	37	8	15	-	-	++	+
1050		*Oceanobacillus picturae*	volcanic salts	MA	37	8	15	-	-	-	-
1051		*Oceanobacillus picturae*	volcanic salts	MA	37	8	15	-	-	+	-
1052	CP-45	*Bacillus velezensis*	fermented food (soybean)	MA	25	5	3	+++	-	-	+++
1053	CP-53	*Bacillus velezensis*	fermented food (soybean)	MA	25	5	3	+++	-	-	+++
1054		*Bacillus velezensis*	fermented food (soybean)	MA	25	5	3	+++	-	-	+++
1055		*Bacillus velezensis*	fermented food (soybean)	MA	25	5	3	+++	-	-	+++
1056		*Bacillus velezensis*	fermented food (soybean)	MA	25	5	3	+++	-	-	+++
1057		*Bacillus velezensis*	fermented food (soybean)	MA	25	5	3	+++	-	-	+++
1058		*Bacillus velezensis*	fermented food (soybean)	MA	25	5	3	+++	-	-	+++
1059		*Bacillus velezensis*	fermented food (soybean)	MA	25	5	3	+++	-	-	+++
1060		*Bacillus velezensis*	fermented food (soybean)	MA	25	5	3	+++	-	-	+++
1061		*Bacillus velezensis*	fermented food (soybean)	MA	25	5	3	+++	-	-	+++

1062		*Bacillus velezensis*	fermented food (soybean)	MA	25	5	3	+++	-	-	+++
1063	CP-47	*Bacillus velezensis*	fermented food (soybean)	MA	25	5	3	+++	-	-	+++
1064		*Bacillus safensis*	fermented food (soybean)	MA	25	5	3	-	-	+	+++
1065		*Staphylococcus epidermidis*	fermented food (soybean)	MA	25	5	3	-	-	-	++
1066		*Bacillus amyloliquefaciens*	fermented food (soybean)	MA	25	5	3	++	-	-	+++
1067		*Bacillus amyloliquefaciens*	fermented food (soybean)	MA	25	5	3	+	-	-	+++
1068	CP-52	*Bacillus amyloliquefaciens*	fermented food (soybean)	MA	25	5	3	+++	-	-	+++
1069		*Pseudoalteromonas arctica*	beach seawater	MA	25	5	3	+	-	+++	+++
1070		*Sulfitobacter pontiacus*	beach seawater	MA	25	5	3	-	-	-	-
1071		*Psychrobacter fjordensis*	Hitakatsu port seawater	MA	25	5	3	-	-	-	-
1072		*Shewanella vesiculosa*	Hitakatsu port seawater	MA	25	5	3	+	-	+++	+++
1073		*Psychrobacter nivimaris*	Hitakatsu port seawater	MA	25	5	3	-	-	++	+
1074		*Ewingella americana*	Hitakatsu port seawater	MA	25	5	3	-	-	-	-
1075		*Salinibacterium amurskyense*	Hitakatsu port seawater	MA	25	5	3	-	-	+	+
1076		*Psychrobacter faecalis*	Hitakatsu port seawater	MA	25	5	3	-	-	++	-
1077		*Brevundimonas nasdae*	Hitakatsu port seawater	MA	25	5	3	+	-	-	-
1078		*Escherichia fergusonii*	Hotarunoyu hot spring	MA	25	5	3	-	-	-	-
1079		*Sulfitobacter pontiacus*	beach soil	MA	25	5	3	-	-	-	-
1080		*Pseudomonas reinekei*	beach soil	MA	25	5	3	-	-	++	++
1081		*Bacillus wiedmannii*	beach soil	MA	25	5	3	-	-	-	-
1082		*Arthrobacter mysorens*	beach soil	MA	25	5	3	-	-	-	+++
1083		*Bacillus wiedmannii*	Izhara pension hill soil	MA	25	5	3	+++	-	++	+++
1084		*Pseudomonas brassicacearum subsp. neoaurantiaca*	Izhara pension hill soil	MA	25	5	3	-	-	++	+
1085		*Bacillus aryabhattai*	cypress forest soil soil	MA	25	5	3	+++	-	-	+++
1086		*Bacillus weihenstephanensis*	cypress forest soil soil	MA	25	5	3	-	-	+	-
1087	CP-37	*Bacillus weihenstephanensis*	cypress forest soil soil	MA	25	5	3	+++	-	++	+++
1088		*Bacillus wiedmannii*	Korean obsevatory soil	MA	25	5	3	-	-	-	-
1089		*Leclercia adecarboxylata*	Korean obsevatory soil	MA	25	5	3	-	-	-	-
1090		*Pseudomonas reinekei*	Korean obsevatory soil	MA	25	5	3	-	-	+++	++
1091		*Erwinia billingiae*	Korean obsevatory soil	MA	25	5	3	-	-	-	-
1092		*Oceanimonas doudoroffii*	beach soil	MA	25	5	3	-	-	-	-
1093		*Sulfitobacter pontiacus*	beach soil	MA	25	5	3	-	-	-	-
1094		*Sulfitobacter pontiacus*	beach soil	MA	25	5	3	-	-	-	-
1095		*Alteromonas marina*	Haegando port seawater	MA	25	5	3	+	-	+++	+++
1096		*Salinicola salarius*	Haegando port seawater	MA	25	5	3	-	-	+	-
1097		*Pseudoalteromonas shioyasakiensis*	Haegando port seawater	MA	25	5	3	-	-	+++	+++
1098		*Salinimonas lutimaris*	Haegando port seawater	MA	25	5	3	-	-	+++	+++
1099		*Cobetia pacifica*	Haegando port seawater	MA	25	5	3	-	-	-	-
1100		*Pseudoalteromonas issachenkonii*	Haegando port seawater	MA	25	5	3	-	-	+++	+++
1101		*Cobetia pacifica*	Yeongi village inner harbor seawater	MA	25	5	3	+	-	-	-
1102		*Cobetia amphilecti*	Yeongi village inner harbor seawater	MA	25	5	3	-	-	-	-

1103		*Salinicola salarius*	Yeongi village inner harbor seawater	MA	25	5	3	-	-	-	-
1104		*Pseudoalteromonas shioyasakiensis*	Yeongi village inner harbor seawater	MA	25	5	3	+	-	++	+++
1105		*Bacillus velezensis*	fermented food (soybean)	MA	25	7	3	+++	-	-	+
1106		*Bacillus velezensis*	fermented food (soybean)	MA	25	7	3	+++	-	-	+
1107		*Bacillus velezensis*	fermented food (soybean)	MA	25	7	3	+++	-	-	+
1108		*Bacillus velezensis*	fermented food (soybean)	MA	25	7	3	+++	-	-	+
1109		*Bacillus velezensis*	fermented food (soybean)	MA	25	7	3	+++	-	-	+
1110		*Bacillus velezensis*	fermented food (soybean)	MA	25	7	3	+++	-	-	+
1111		*Bacillus safensis*	fermented food (soybean)	MA	25	7	3	-	-	+	-
1112		*Bacillus velezensis*	fermented food (soybean)	MA	25	7	3	++	-	-	+
1113		*Bacillus velezensis*	fermented food (soybean)	MA	25	7	3	+++	-	-	+
1114		*Bacillus velezensis*	fermented food (soybean)	MA	25	7	3	+++	-	-	-
1115		*Bacillus stratosphericus*	fermented food (soybean)	MA	25	7	3	-	-	+	-
1116		*Staphylococcus warneri*	fermented food (soybean)	MA	25	7	3	-	-	-	-
1117	CP-58	*Bacillus amyloliquefaciens*	fermented food (soybean)	MA	25	7	3	+++	-	-	+++
1118		*Bacillus amyloliquefaciens*	fermented food (soybean)	MA	25	7	3	+	-	-	+++
1119		*Staphylococcus warneri*	fermented food (soybean)	MA	25	7	3	-	-	-	-
1120		*Sulfitobacter pontiacus*	beach seawater	MA	25	7	3	-	-	-	-
1121		*Glaciecola nitratireducens*	beach seawater	MA	25	7	3	+++	-	++	+++
1122		*Sediminicola luteus*	beach seawater	MA	25	7	3	+	-	+	+++
1123		*Pseudoalteromonas arctica*	Hitakatsu port seawater	MA	25	7	3	+	-	+++	+
1124		*Pseudoalteromonas paragorgicola*	Hitakatsu port seawater	MA	25	7	3	+	-	+++	++
1125		*Pseudoalteromonas mariniglutinosa*	Hitakatsu port seawater	MA	25	7	3	+++	-	++	+++
1126		*Cyclobacterium caenipelagi*	Hitakatsu port seawater	MA	25	7	3	-	-	-	-
1127		*Roseovarius aestuarii*	Hitakatsu port seawater	MA	25	7	3	-	-	-	-
1128		*Salinibacterium amurskyense*	Hitakatsu port seawater	MA	25	7	3	-	-	-	-
1129		*Rhodococcus cercidiphylli*	Hitakatsu port seawater	MA	25	7	3	-	-	++	-
1130		*Pseudoalteromonas issachenkonii*	Hitakatsu port seawater	MA	25	7	3	+++	-	+	+++
1131		*Cellulophaga fucicola*	Hitakatsu port seawater	MA	25	7	3	+	-	-	-
1132		*Pseudorhodobacter collinsensis*	Hitakatsu port seawater	MA	25	7	3	-	-	-	-
1133		*Bacillus firmus*	beach soil	MA	25	7	3	++	-	+	+++
1134		*Sulfitobacter pontiacus*	beach soil	MA	25	7	3	-	-	+	-
1135		*Arenibacter palladensis*	beach soil	MA	25	7	3	-	-	-	-
1136		*Marinobacter sediminum*	beach soil	MA	25	7	3	-	-	-	-
1137		*Pseudomonas reinekei*	beach soil	MA	25	7	3	-	-	++	+
1138		*Streptomyces microflavus*	beach soil	MA	25	7	3	+	-	-	+
1139		*Algoriphagus locisalis*	beach soil	MA	25	7	3	-	-	++	+
1140		*Shewanella xiamenensis*	beach soil	MA	25	7	3	-	-	-	+++
1141	CP-59	*Bacillus wiedmannii*	Izhara pension hill soil	MA	25	7	3	+++	-	+++	+++
1142		*Bacillus vietnamensis*	Izhara pension hill soil	MA	25	7	3	++	-	++	+++

1143		*Microbacterium hydrocarbonoxydans*	Izhara pension hill soil	MA	25	7	3	++	-	+	+
1144		*Pseudomonas koreensis*	Izhara pension hill soil	MA	25	7	3	-	-	-	++
1145		*Arthrobacter humicola*	Izhara pension hill soil	MA	25	7	3	-	-	+	+++
1146		*Bacillus aquimaris*	Izhara pension hill soil	MA	25	7	3	+++	-	-	+++
1147		*Fictibacillus halophilus*	beach soil	MA	25	7	3	++	-	-	+++
1148		*Bacillus algicola*	beach soil	MA	25	7	3	++	-	-	+++
1149		*Bacillus algicola*	beach soil	MA	25	7	3	+	-	-	+++
1150	CP-60	*Bacillus hwajinpoensis*	beach soil	MA	25	7	3	+++	-	+++	+++
1151	CP-46	*Bacillus wiedmannii*	cypress forest soil soil	MA	25	7	3	+++	-	+++	+++
1152		*Bacillus firmus*	Korean obsevatory soil	MA	25	7	3	++	-	-	+
1153		*Pseudomonas reinekei*	Korean obsevatory soil	MA	25	7	3	-	-	++	++
1154		*Marinobacterium profundum*	beach soil	MA	25	7	3	-	-	-	-
1155		*Sulfitobacter pontiacus*	beach soil	MA	25	7	3	-	-	+	-
1156		*Rheinheimera aquimaris*	beach soil	MA	25	7	3	++	-	+++	+
1157		*Pseudoalteromonas marina*	Yeongi village inner harbor seawater	MA	25	7	3	-	-	+++	+++
1158		*Octadecabacter ascidiaceicola*	Yeongi village inner harbor seawater	MA	25	7	3	-	-	-	+
1159		*Pseudoalteromonas marina*	Yeongi village inner harbor seawater	MA	25	7	3	++	-	+++	+
1160		*Vibrio owensii*	Yeongi village inner harbor seawater	MA	25	7	3	-	-	+++	+++
1161		*Pseudoalteromonas spongiae*	Yeongi village inner harbor seawater	MA	25	7	3	-	-	+++	+++
1162		*Salinimicrobium terrae*	Haegando port seawater	MA	25	7	3	-	-	+++	+
1163		*Pseudoalteromonas issachenkonii*	Haegando port seawater	MA	25	7	3	+++	-	+++	+++
1164		*Pseudoalteromonas hodoensis*	Haegando port seawater	MA	25	7	3	+++	-	+++	+++
1165		*Pseudoalteromonas gelatinilytica*	Haegando port seawater	MA	25	7	3	+	-	+++	+++
1166		*Alteromonas mediterranea*	Haegando port seawater	MA	25	7	3	+	-	+++	+++
1167		*Cobetia amphilecti*	Haegando port seawater	MA	25	7	3	-	-	-	-
1168		*Salinicola salarius*	Yeongi village inner harbor seawater	MA	25	7	3	-	-	++	-
1169		*Halomonas denitrificans*	Yeongi village inner harbor seawater	MA	25	7	3	-	-	-	-
1170		*Salinicola salarius*	Yeongi village inner harbor seawater	MA	25	7	3	-	-	-	-
1171		*Sulfitobacter faviae*	Yeongi village inner harbor seawater	MA	25	7	3	-	-	+++	+++
1172		*Salinimicrobium terrae*	Yeongi village inner harbor seawater	MA	25	7	3	-	-	+++	+
1173		*Vibrio alginolyticus*	Yeongi village inner harbor seawater	MA	25	7	3	+++	-	-	+++
1174		*Bacillus aerius*	fermented food (soybean)	MA	25	9	3	-	-	-	+++
1175		*Staphylococcus warneri*	fermented food (soybean)	MA	25	9	3	-	-	-	-
1176		*Bacillus wiedmannii*	fermented food (soybean)	MA	25	9	3	-	-	++	+++
1177	CP-48	*Bacillus wiedmannii*	beach seawater	MA	25	9	3	+++	-	+++	+++
1178		*Sulfitobacter pontiacus*	beach seawater	MA	25	9	3	-	-	-	-
1179		*Pseudoalteromonas issachenkonii*	Hitakatsu port seawater	MA	25	9	3	-	-	+	+++
1180		*Pseudoalteromonas issachenkonii*	Hitakatsu port seawater	MA	25	9	3	+++	-	+	+++
1181		*Pseudoalteromonas paragorgicola*	Hitakatsu port seawater	MA	25	9	3	-	-	+++	+++
1182		*Micrococcus luteus*	Hitakatsu port seawater	MA	25	9	3	-	-	+++	++
1183		*Pseudoalteromonas neustonica*	Hitakatsu port seawater	MA	25	9	3	-	-	+++	++
1184		*Pseudoalteromonas issachenkonii*	Hitakatsu port seawater	MA	25	9	3	+	-	++	++

1185		*Salinibacterium amurskyense*	Hitakatsu port seawater	MA	25	9	3	-	-	-	+
1186		*Arenibacter palladensis*	Hitakatsu port seawater	MA	25	9	3	-	-	+	-
1187		*Pseudoalteromonas issachenkonii*	Hitakatsu port seawater	MA	25	9	3	+++	-	++	+++
1188		*Rhodococcus cercidiphylli*	Hitakatsu port seawater	MA	25	9	3	-	-	+++	-
1189	CP-49	*Bacillus hwajinpoensis*	beach soil	MA	25	9	3	+++	-	+++	+++
1190		*Marinobacter sediminum*	beach soil	MA	25	9	3	-	-	+++	-
1191		*Bacillus algicola*	beach soil	MA	25	9	3	-	-	-	-
1192		*Sulfitobacter pontiacus*	beach soil	MA	25	9	3	-	-	+	-
1193		*Pseudomonas reinekei*	beach soil	MA	25	9	3	-	-	-	+++
1194		*Arthrobacter mysorens*	beach soil	MA	25	9	3	-	-	-	+++
1195		*Halomonas songnenensis*	beach soil	MA	25	9	3	-	-	-	+++
1196		*Shewanella xiamenensis*	beach soil	MA	25	9	3	-	-	+++	+++
1197		*Pseudomonas reinekei*	Izhara pension hill soil	MA	25	9	3	-	-	-	+
1198		*Bacillus wiedmannii*	Izhara pension hill soil	MA	25	9	3	-	-	+	+++
1199		*Bacillus wiedmannii*	Izhara pension hill soil	MA	25	9	3	-	-	+++	+++
1200		*Bacillus marisflavi*	Izhara pension hill soil	MA	25	9	3	-	-	+	+++
1201		*Pseudomonas reinekei*	Izhara pension hill soil	MA	25	9	3	-	-	-	+
1202		*Bacillus firmus*	Izhara pension hill soil	MA	25	9	3	+	-	-	++
1203		*Pseudomonas reinekei*	Izhara pension hill soil	MA	25	9	3	-	-	-	+
1204		*Bacillus cibi*	Izhara pension hill soil	MA	25	9	3	++	-	-	+++
1205		*Bacillus algicola*	beach soil	MA	25	9	3	+	-	-	++
1206		*Sulfitobacter pontiacus*	beach soil	MA	25	9	3	-	-	-	-
1207		*Bacillus weihenstephanensis*	cypress forest soil soil	MA	25	9	3	+++	-	+	+++
1208	CP-50	*Bacillus weihenstephanensis*	cypress forest soil soil	MA	25	9	3	+++	-	++	+++
1209		*Pseudomonas reinekei*	cypress forest soil soil	MA	25	9	3	-	-	+++	+
1210	CP-51	*Bacillus cereus*	Korean obsevatory soil	MA	25	9	3	+++	-	+++	+++
1211		*[Brevibacterium] frigoritolerans*	Korean obsevatory soil	MA	25	9	3	-	-	-	++
1212		*Oceanimonas doudoroffii*	beach soil	MA	25	9	3	-	-	-	-
1213		*Sulfitobacter pontiacus*	beach soil	MA	25	9	3	-	-	-	+
1214		*Marinobacterium rhizophilum*	beach soil	MA	25	9	3	-	-	-	-
1215		*Pseudoalteromonas shioyasakiensis*	Yeongi village inner harbor seawater	MA	25	9	3	-	-	+++	-
1216		*Vibrio rotiferianus*	Yeongi village inner harbor seawater	MA	25	9	3	-	-	+++	+++
1217		*Pseudoalteromonas marina*	Yeongi village inner harbor seawater	MA	25	9	3	-	-	+++	+++
1218		*Sulfitobacter faviae*	Yeongi village inner harbor seawater	MA	25	9	3	-	-	-	-
1219		*Halomonas xianhensis*	Yeongi village inner harbor seawater	MA	25	9	3	-	-	-	-
1220		*Salinimicrobium terrae*	Haegando port seawater	MA	25	9	3	-	-	++	+
1221		*Pseudoalteromonas shioyasakiensis*	Haegando port seawater	MA	25	9	3	-	-	+++	+++
1222		*Salinimonas lutimaris*	Haegando port seawater	MA	25	9	3	-	-	+++	+++
1223		*Marinomonas posidonica*	Haegando port seawater	MA	25	9	3	-	-	++	-
1224		*Pseudoalteromonas shioyasakiensis*	Haegando port seawater	MA	25	9	3	-	-	+	+

1225	*Pseudoalteromonas phenolica*	Haegando port seawater	MA	25	9	3	-	-	+	-
1226	*Pseudoalteromonas issachenkonii*	Haegando port seawater	MA	25	9	3	-	-	+++	+++
1227	*Pseudoalteromonas rubra*	Yeongi village harbor seawater	MA	25	9	3	-	-	+++	-
1228	*Shewanella waksmanii*	Yeongi village harbor seawater	MA	25	9	3	-	-	+++	+++
1229	*Halomonas denitrificans*	Yeongi village harbor seawater	MA	25	9	3	-	-	-	+
1230	*Salinicola salarius*	Yeongi village harbor seawater	MA	25	9	3	-	-	-	-
1231	*Cobetia marina*	Yeongi village harbor seawater	MA	25	9	3	-	-	-	-
1232	*Planomicrobium flavidum*	Yeongi village harbor seawater	MA	25	9	3	-	-	-	+++
1233	*Enterobacter ludwigii*	Geumil beach crab haegeum water	MA	30	5	3	-	-	-	-
1234	*Leclercia adecarboxylata*	Geumil beach crab haegeum water	MA	30	5	3	-	-	-	-
1235	*Shewanella algae*	Geumil beach crab haegeum water	MA	30	5	3	-	-	+++	+++
1236	*Shewanella seohaensis*	Geumil beach crab haegeum water	MA	30	7	3	++	-	+++	+++
1237	*Aeromonas dhakensis*	Geumil beach crab haegeum water	MA	30	7	3	+++	-	+++	-
1238	*Bacillus firmus*	Geumil beach red pepper field soil	MA	30	7	3	+	-	+	+++
1239	*Exiguobacterium acetylicum*	Geumil beach red pepper field soil	MA	30	7	3	+++	-	-	+++
1240	*Enterobacter cancerogenus*	Geumil beach red pepper field soil	MA	30	7	3	-	-	-	-
1241	*Exiguobacterium acetylicum*	Geumil beach red pepper field soil	MA	30	7	3	+++	-	-	+++
1242	*Bacillus aryabhattai*	Geumil beach red pepper field soil	MA	30	7	3	+++	-	-	-
1243	*Bacillus megaterium*	Geumil beach wild grass soil	MA	30	7	3	+++	-	-	-
1244	*Bacillus proteolyticus*	Iljung port pine community soil	MA	30	7	3	-	-	+	-
1245	*Bacillus aryabhattai*	Iljung port pine community soil	MA	30	7	3	+++	-	-	-
1246	*Bacillus cereus*	Iljung port pine community soil	MA	30	7	3	-	-	-	-
1247	*Vibrio alginolyticus*	Sadong port seawater	MA	30	7	3	+++	-	-	-
1248	*Pseudoalteromonas shioyasakiensis*	Sadong port seawater	MA	30	7	3	++	-	+++	-
1249	*Nautella italica*	Sadong port seawater	MA	30	9	3	-	-	-	-
1250	*Sulfitobacter dubius*	Sadong port seawater	MA	30	9	3	-	-	-	-
1251	*Nautella italica*	Sadong port seawater	MA	30	9	3	-	-	-	-
1252	*Nautella italica*	Sadong port seawater	MA	30	9	3	-	-	-	-
1253	*Bacillus subtilis*	fermented food (soybean)	MA	37	5	3	+++	-	-	+
1254	*Bacillus velezensis*	fermented food (soybean)	MA	37	5	3	+++	-	-	+
1255	*Bacillus velezensis*	fermented food (soybean)	MA	37	5	3	+++	-	-	+
1256	*Bacillus amyloliquefaciens*	fermented food (soybean)	MA	37	5	3	+++	-	-	+
1257	*Bacillus velezensis*	fermented food (soybean)	MA	37	5	3	+++	-	-	+
1258	*Bacillus velezensis*	fermented food (soybean)	MA	37	5	3	+++	-	-	+
1259	*Bacillus velezensis*	traditional fermented food (soybean)	MA	37	5	3	+++	-	-	-
1260	*Bacillus amyloliquefaciens*	traditional fermented food (soybean)	MA	37	5	3	+++	-	-	-
1261	*Staphylococcus epidermidis*	traditional fermented food (soybean)	MA	37	5	3	-	-	++	+++
1262	*Bacillus amyloliquefaciens*	fermented food (soybean)	MA	37	5	3	+++	-	-	-
1263	*Staphylococcus epidermidis*	fermented food (soybean)	MA	37	5	3	-	-	+	-
1264	*Bacillus paralicheniformis*	fermented food (soybean)	MA	37	5	3	-	-	-	+++
1265	*Staphylococcus epidermidis*	apricot seaweed	MA	37	5	3	-	-	++	++
1266	*Staphylococcus warneri*	beach seawater	MA	37	5	3	-	-	+++	-

1267	*Bacillus cereus*	beach seawater	MA	37	5	3	-	-	+++	+++
1268	*Psychrobacter pulmonis*	Hitakatsu port seawater	MA	37	5	3	-	-	+++	-
1269	*Serratia grimesii*	Hitakatsu port seawater	MA	37	5	3	-	-	+	+++
1270	*Pseudomonas marincola*	beach soil	MA	37	5	3	-	-	+	-
1271	*Streptomyces rameus*	Izhara pension hill soil	MA	37	5	3	++	-	++	-
1272	*Streptomyces chilikensis*	Izhara pension hill soil	MA	37	5	3	+++	-	+	-
1273	*Bacillus wiedmannii*	cypress forest soil soil	MA	37	5	3	++	-	-	+
1274	*Bacillus tianmuensis*	cypress forest soil soil	MA	37	5	3	++	-	-	+++
1275	*Brevibacterium frigoritolerans*	Korean obsevatory soil	MA	37	5	3	-	-	-	-
1276	*Isoptericola variabilis*	Korean obsevatory soil	MA	37	5	3	+++	-	-	+
1277	*Oceanimonas doudoroffii*	beach soil	MA	37	5	3	-	-	++	++
1278	*Ruegeria arenilitoris*	beach soil	MA	37	5	3	-	-	-	++
1279	*Enterobacter xiangfangensis*	Geumil beach crab haegeum water	MA	37	5	3	-	-	-	+++
1280	*Kurthia gibsonii*	Geumil beach crab haegeum water	MA	37	5	3	-	-	-	+
1281	*Kurthia gibsonii*	Geumil beach crab haegeum water	MA	37	5	3	-	-	-	-
1282	*Enterobacter xiangfangensis*	Geumil beach crab haegeum water	MA	37	5	3	-	-	-	-
1283	*Shewanella algae*	Geumil beach crab haegeum water	MA	37	5	3	+	-	+	+++
1284	*Kurthia gibsonii*	Geumil beach crab haegeum water	MA	37	5	3	-	-	-	-
1285	*Enterobacter xiangfangensis*	Geumil beach crab haegeum water	MA	37	5	3	-	-	+	-
1286	*Vibrio natriegens*	Haegando port seawater	MA	37	5	3	+++	-	+++	-
1287	*Salinicola salarius*	Haegando port seawater	MA	37	5	3	-	-	++	+++
1288	*Halomonas xianhensis*	Haegando port seawater	MA	37	5	3	-	-	-	+++
1289	*Salinicola salarius*	Haegando port seawater	MA	37	5	3	-	-	++	-
1290	*Pseudoalteromonas shioyasakiensis*	Haegando port seawater	MA	37	5	3	-	-	+++	+
1291	*Cobetia pacifica*	Haegando port seawater	MA	37	5	3	-	-	-	+
1292	*Vibrio harveyi*	Haegando port seawater	MA	37	5	3	+++	-	++	+++
1293	*Halomonas xianhensis*	Haegando port seawater	MA	37	5	3	-	-	+	-
1294	*Vibrio alginolyticus*	Yeongi village harbor seawater	MA	37	5	3	+++	-	-	-
1295	*Cobetia amphilecti*	Yeongi village harbor seawater	MA	37	5	3	-	-	-	-
1296	*Cobetia pacifica*	Yeongi village harbor seawater	MA	37	5	3	-	-	+	-
1297	*Salinicola salarius*	Yeongi village harbor seawater	MA	37	5	3	-	-	+	-
1298	*Halomonas xianhensis*	Yeongi village harbor seawater	MA	37	5	3	+++	-	-	-
1299	*Salinicola salarius*	Yeongi village harbor seawater	MA	37	5	3	+++	-	+	-
1300	*Alteromonas macleodii*	Yeongi village harbor seawater	MA	37	5	3	+++	-	+++	+
1301	*Vibrio alginolyticus*	Yeongi village harbor seawater	MA	37	5	3	+++	-	+++	+
1302	*Salinicola salarius*	Yeongi village harbor seawater	MA	37	5	3	-	-	+	-
1303	*Bacillus subtilis*	fermented food (soybean)	MA	37	7	3	+++	-	-	+
1304	*Bacillus velezensis*	fermented food (soybean)	MA	37	7	3	+++	-	-	+
1305	*Bacillus velezensis*	fermented food (soybean)	MA	37	7	3	+++	-	-	+
1306	*Bacillus velezensis*	fermented food (soybean)	MA	37	7	3	+++	-	-	+
1307	*Bacillus velezensis*	fermented food (soybean)	MA	37	7	3	+++	-	-	+
1308	*Bacillus velezensis*	fermented food (soybean)	MA	37	7	3	+++	-	-	+

1309		*Bacillus velezensis*	fermented food (soybean)	MA	37	7	3	+++	-	-	+
1310		*Bacillus velezensis*	fermented food (soybean)	MA	37	7	3	+++	-	-	+
1311		*Bacillus subtilis*	fermented food (soybean)	MA	37	7	3	+++	-	-	+++
1312		*Staphylococcus epidermidis*	traditional fermented food (soybean)	MA	37	7	3	-	-	++	++
1313		*Bacillus velezensis*	traditional fermented food (soybean)	MA	37	7	3	+++	-	-	+
1314		*Bacillus amyloliquefaciens*	traditional fermented food (soybean)	MA	37	7	3	+++	-	-	+
1315		*Staphylococcus epidermidis*	fermented food (soybean)	MA	37	7	3	-	-	-	-
1316		*Nocardioides marinisabuli*	beach seawater	MA	37	7	3	-	-	-	-
1317		*Marinobacter sediminum*	beach seawater	MA	37	7	3	-	-	+++	-
1318		*Bacillus wiedmannii*	Hitakatsu port seawater	MA	37	7	3	-	-	-	+
1319		*Pseudoalteromonas issachenkonii*	Hitakatsu port seawater	MA	37	7	3	-	-	++	+
1320		*Cyclobacterium caenipelagi*	Hitakatsu port seawater	MA	37	7	3	-	-	-	-
1321		*Bizionia paragorgiae*	Hitakatsu port seawater	MA	37	7	3	-	-	+	++
1322		*Bacillus hwajinpoensis*	beach soil	MA	37	7	3	+++	-	+	+
1323		*Marinobacter sediminum*	beach soil	MA	37	7	3	-	-	+++	-
1324	CP-54	*Bacillus firmus*	beach soil	MA	37	7	3	++	-	-	+++
1325		*Bacillus hwajinpoensis*	beach soil	MA	37	7	3	+++	-	+	-
1326		*Isoptericola variabilis*	beach soil	MA	37	7	3	++	-	-	++
1327		*Paracoccus marcusii*	beach soil	MA	37	7	3	-	-	+	-
1328		*Bacillus gibsonii*	Izhara pension hill soil	MA	37	7	3	-	-	-	++
1329		*Streptomyces bellus*	Izhara pension hill soil	MA	37	7	3	++	-	-	+++
1330		*Bacillus aerius*	Izhara pension hill soil	MA	37	7	3	-	-	++	+++
1331		*Bacillus wiedmannii*	Izhara pension hill soil	MA	37	7	3	+++	-	-	+++
1332		*Bacillus berkeleyi*	beach soil	MA	37	7	3	+	-	-	+
1333		*Bacillus wiedmannii*	cypress forest soil soil	MA	37	7	3	+++	-	-	-
1334		*Bacillus wiedmannii*	cypress forest soil soil	MA	37	7	3	+++	-	-	++
1335		*Bacillus aryabhattai*	cypress forest soil soil	MA	37	7	3	-	-	-	+++
1336		*Bacillus niacini*	Korean obsevatory soil	MA	37	7	3	-	-	-	-
1337		*Microbacterium hydrocarbonoxydans*	Korean obsevatory soil	MA	37	7	3	+++	-	-	-
1338		*Planococcus salinarum*	beach soil	MA	37	7	3	+	-	-	+++
1339		*Pseudomonas taeanensis*	beach soil	MA	37	7	3	-	-	-	+++
1340		*Bacillus berkeleyi*	beach soil	MA	37	7	3	-	-	-	+++
1341		*Pseudomonas taeanensis*	beach soil	MA	37	7	3	-	-	-	++
1342		*Planomicrobium alkanoclasticum*	beach soil	MA	37	7	3	+	-	-	+++
1343		*Antarctobacter jejuensis*	beach soil	MA	37	7	3	-	-	-	-
1344	CP-55	*Exiguobacterium indicum*	Geumil beach crab haegeum water	MA	37	7	3	+++	-	-	+++
1345		*Acinetobacter pittii*	Geumil beach red pepper field soil	MA	37	7	3	-	-	+++	-
1346		*Bacillus aryabhattai*	Geumil beach red pepper field soil	MA	37	7	3	-	-	-	-
1347		*Pantoea ananatis*	Geumil beach wild grass soil	MA	37	7	3	-	-	+	-
1348		*Bacillus subtilis*	Geumil beach wild grass soil	MA	37	7	3	+++	-	++	-
1349		*Bacillus aryabhattai*	Geumil beach wild grass soil	MA	37	7	3	+++	-	-	-
1350		*Bacillus aryabhattai*	Geumil beach wild grass soil	MA	37	7	3	+++	-	-	-

1351	*Bacillus wiedmannii*	Iljung port pine community soil	MA	37	7	3	+++	-	-	-
1352	*Bacillus aryabhattai*	Iljung port pine community soil	MA	37	7	3	+++	-	-	-
1353	*Bacillus megaterium*	Iljung port pine community soil	MA	37	7	3	+	-	-	-
1354	*Vibrio alginolyticus*	Sadong port seawater	MA	37	7	3	+++	-	-	-
1355	*Vibrio harveyi*	Sadong port seawater	MA	37	7	3	-	-	-	+
1356	*Marinomonas communis*	Sadong port seawater	MA	37	7	3	-	-	-	-
1357	*Nautella italica*	Sadong port seawater	MA	37	7	3	+	-	-	-
1358	*Alteromonas tagae*	Yeongi village harbor seawater	MA	37	7	3	+++	-	+++	+++
1359	*Pseudoalteromonas carrageenovora*	Yeongi village harbor seawater	MA	37	7	3	+++	-	+++	+
1360	*Pseudoalteromonas marina*	Yeongi village harbor seawater	MA	37	7	3	++	-	+++	+
1361	*Pseudoalteromonas spongiae*	Yeongi village harbor seawater	MA	37	7	3	-	-	++	+++
1362	*Alteromonas marina*	Yeongi village harbor seawater	MA	37	7	3	-	-	++	-
1363	*Microbacterium sediminis*	Yeongi village harbor seawater	MA	37	7	3	-	-	-	-
1364	*Erythrobacter citreus*	Yeongi village harbor seawater	MA	37	7	3	-	-	+++	+
1365	*Halomonas xianhensis*	Haegando port seawater	MA	37	7	3	-	-	-	-
1366	*Pseudoalteromonas issachenkonii*	Haegando port seawater	MA	37	7	3	+++	-	-	+++
1367	*Cobetia pacifica*	Haegando port seawater	MA	37	7	3	-	-	-	-
1368	*Vibrio alginolyticus*	Haegando port seawater	MA	37	7	3	+++	-	-	-
1369	*Salinimicrobium terrae*	Haegando port seawater	MA	37	7	3	++	-	+++	-
1370	*Salinimicrobium terrae*	Haegando port seawater	MA	37	7	3	+++	-	+++	+
1371	*Pseudoalteromonas shioyasakiensis*	Haegando port seawater	MA	37	7	3	+++	-	+	++
1372	*Pseudoalteromonas spongiae*	Haegando port seawater	MA	37	7	3	-	-	++	+++
1373	*Sulfitobacter faviae*	Yeongi village harbor seawater	MA	37	7	3	-	-	+	-
1374	*Halomonas denitrificans*	Yeongi village harbor seawater	MA	37	7	3	+	-	-	-
1375	*Salinimicrobium terrae*	Yeongi village harbor seawater	MA	37	7	3	+++	-	+++	-
1376	*Halomonas xianhensis*	Yeongi village harbor seawater	MA	37	7	3	-	-	-	-
1377	*Sulfitobacter faviae*	Yeongi village harbor seawater	MA	37	7	3	+	-	-	-
1378	*Alteromonas marina*	Yeongi village harbor seawater	MA	37	7	3	+++	-	+++	+++
1379	*Pseudoalteromonas shioyasakiensis*	Yeongi village harbor seawater	MA	37	7	3	+++	-	+++	+
1380	*Bacillus safensis*	fermented food (soybean)	MA	37	9	3	-	-	-	+++
1381	*Bacillus fordii*	traditional fermented food (soybean)	MA	37	9	3	++	-	++	-
1382	*Micrococcus luteus*	fermented food (soybean)	MA	37	9	3	-	-	+	+++
1383	*Marinobacter sediminum*	beach seawater	MA	37	9	3	-	-	+++	-
1384	*Pseudoalteromonas issachenkonii*	Hitakatsu port seawater	MA	37	9	3	+++	-	-	+++
1385	*Cyclobacterium caenipelagi*	Hitakatsu port seawater	MA	37	9	3	-	-	-	-
1386	*Trichococcus pasteurii*	Hitakatsu port seawater	MA	37	9	3	-	-	-	-
1387	*Marinobacter sediminum*	beach soil	MA	37	9	3	-	-	++	-
1388	*Marinobacter algicola*	beach soil	MA	37	9	3	+++	-	+++	-
1389	*Bacillus horneckiae*	beach soil	MA	37	9	3	-	-	+++	-
1390	*Bacillus wiedmannii*	beach soil	MA	37	9	3	+++	-	-	-

1391		*Halomonas songnenensis*	beach soil	MA	37	9	3	-	-	-	-
1392		*Bacillus wiedmannii*	beach soil	MA	37	9	3	-	-	++	++
1393		*Bacillus manliponensis*	Izhara pension hill soil	MA	37	9	3	-	-	-	+
1394		*Bacillus purgationiresistens*	Izhara pension hill soil	MA	37	9	3	-	-	-	+++
1395		*Bacillus megaterium*	Izhara pension hill soil	MA	37	9	3	-	-	-	++
1396		*Bacillus wiedmannii*	Izhara pension hill soil	MA	37	9	3	-	-	++	+++
1397		*Bacillus cibi*	cypress forest soil soil	MA	37	9	3	+	-	-	+++
1398		*Bacillus idriensis*	cypress forest soil soil	MA	37	9	3	+	-	-	++
1399		*Bacillus megaterium*	Korean obsevatory soil	MA	37	9	3	+++	-	-	+++
1400		*Bacillus aryabhattai*	Korean obsevatory soil	MA	37	9	3	+++	-	-	+++
1401		*Brevibacterium frigoritolerans*	Korean obsevatory soil	MA	37	9	3	+	-	-	+
1402		*Lysinibacillus alkaliphilus*	Korean obsevatory soil	MA	37	9	3	-	-	-	+++
1403		*Zobellella aerophila*	beach soil	MA	37	9	3	-	-	-	-
1404		*Marinobacter sediminum*	beach soil	MA	37	9	3	-	-	+++	-
1405		*Marinobacter sediminum*	beach soil	MA	37	9	3	-	-	+++	-
1406		*Pseudomonas taeanensis*	beach soil	MA	37	9	3	++	-	-	-
1407	CP-56	*Bacillus hwajinpoensis*	beach soil	MA	37	9	3	+++	-	+	+++
1408		*Photobacterium rosenbergii*	Sadong port seawater	MA	37	9	3	++	-	+++	-
1409		*Vibrio hepatarius*	Sadong port seawater	MA	37	9	3	+++	-	-	-
1410		*Vibrio alginolyticus*	Sadong port seawater	MA	37	9	3	+++	-	+++	-
1411		*Alteromonas marina*	Sadong port seawater	MA	37	9	3	+++	-	+++	+++
1412		*Salinicola salarius*	Yeongi village harbor seawater	MA	37	9	3	-	-	+	-
1413		*Pseudoalteromonas spongiae*	Yeongi village harbor seawater	MA	37	9	3	-	-	+++	-
1414		*Pseudoalteromonas carrageenovora*	Yeongi village harbor seawater	MA	37	9	3	++	-	+++	-
1415		*Nautella italica*	Yeongi village harbor seawater	MA	37	9	3	-	-	-	-
1416		*Vibrio alginolyticus*	Haegando port seawater	MA	37	9	3	+++	-	-	++
1417		*Alteromonas macleodii*	Haegando port seawater	MA	37	9	3	-	-	-	-
1418		*Vibrio alginolyticus*	Haegando port seawater	MA	37	9	3	+++	-	-	++
1419		*Vibrio alginolyticus*	Haegando port seawater	MA	37	9	3	+++	-	-	++
1420		*Vibrio alginolyticus*	Haegando port seawater	MA	37	9	3	+++	-	-	++
1421		*Vibrio alginolyticus*	Haegando port seawater	MA	37	9	3	+++	-	-	++
1422		*Vibrio alginolyticus*	Haegando port seawater	MA	37	9	3	+++	-	-	++
1423		*Vibrio alginolyticus*	Haegando port seawater	MA	37	9	3	+++	-	-	++
1424		*Halomonas denitrificans*	Yeongi village inner harbor seawater	MA	37	9	3	-	-	+++	++
1425		*Halomonas xianhensis*	Yeongi village inner harbor seawater	MA	37	9	3	-	-	-	-
1426		*Salinicola salarius*	Yeongi village inner harbor seawater	MA	37	9	3	-	-	+	-
1427		*Planomicrobium flavidum*	Yeongi village inner harbor seawater	MA	37	9	3	-	-	-	+++
1428		*Planomicrobium okeanokoites*	Yeongi village inner harbor seawater	MA	37	9	3	-	-	-	+++
1429		*Vibrio alginolyticus*	Yeongi village inner harbor seawater	MA	37	9	3	+++	-	-	-
1430		*Bacillus thermolactis*	Geumil beach red pepper field soil	MA	50	7	3	-	-	-	+++
1431		*Bacillus subtilis*	Geumil beach red pepper field soil	MA	50	7	3	+++	-	-	+++
1432		*Bacillus xiamenensis*	Geumil beach red pepper field soil	MA	50	7	3	-	-	-	+++

1433	*Bacillus thermocopriae*	Geumil beach red pepper field soil	MA	50	7	3	-	-	-	+++
1434	*Bacillus safensis*	Geumil beach wild grass soil	MA	50	7	3	++	-	-	+
1435	*Bacillus aerius*	Geumil beach wild grass soil	MA	50	7	3	-	-	-	+
1436	*Bacillus aerius*	Geumil beach wild grass soil	MA	50	7	3	-	-	-	+++
1437	*Bacillus australimaris*	Geumil beach wild grass soil	MA	50	7	3	-	-	-	-
1438	*Bacillus safensis*	Iljung port pine community soil	MA	50	7	3	-	-	-	-
1439	*Bacillus haynesii*	Iljung port pine community soil	MA	50	7	3	+++	-	+++	-
1440	*Bacillus subtilis*	Iljung port pine community soil	MA	50	7	3	-	-	-	-
1441	*Halobacillus trueperi*	Korean Topan Salt	MA	37	7	3	-	-	-	-
1442	*Halobacillus trueperi*	Korean Topan Salt	MA	37	7	3	-	-	-	-
1443	*Halobacillus alkaliphilus*	Korean Topan Salt	MA	37	7	3	-	-	-	-
1444	*Halobacillus trueperi*	Korean Topan Salt	MA	37	7	3	-	-	-	-
1445	*Halobacillus trueperi*	Korean Topan Salt	MA	37	7	3	-	-	++	-
1446	*Halobacillus trueperi*	Korean Topan Salt	MA	37	7	3	-	-	-	-
1447	*Halobacillus trueperi*	Korean Topan Salt	MA	37	7	3	-	-	-	-
1448	*Halobacillus trueperi*	Korean Topan Salt	MA	37	7	3	-	-	-	-
1449	*Halobacillus trueperi*	Korean Topan Salt	MA	37	7	3	-	-	-	-
1450	*Halobacillus mangrovi*	Korean Topan Salt	MA	37	7	3	-	-	-	-
1451	*Halobacillus mangrovi*	Korean Topan Salt	MA	37	7	3	-	-	-	-
1452	*Halobacillus mangrovi*	Korean Topan Salt	MA	37	7	3	-	-	-	-
1453	*Halobacillus alkaliphilus*	Korean Topan Salt	MA	37	7	3	-	-	-	-
1454	*Halobacillus alkaliphilus*	Korean Topan Salt	MA	37	7	3	-	-	-	-
1455	*Halobacillus trueperi*	Korean Topan Salt	MA	37	7	3	-	-	+	-
1456	*Halobacillus alkaliphilus*	Korean Topan Salt	MA	37	7	3	-	-	-	-
1457	*Halobacillus alkaliphilus*	Korean Topan Salt	MA	37	7	3	-	-	-	-
1458	*Halobacillus alkaliphilus*	Korean Topan Salt	MA	37	7	3	-	-	-	-
1459	*Bacillus berkeleyi*	Sinan taepyeong salt field	MA	37	7	3	-	-	-	-
1460	*Bacillus cibi*	Daejeo ecological park wetland soil	MA	37	7	3	-	-	-	-
1461	*Bacillus subtilis*	Seokmodo hot spring	MA	37	7	3	+++	-	-	-
1462	*Bacillus subtilis*	Seokmodo hot spring	MA	37	7	3	+++	-	-	-
1463	*Halobacillus alkaliphilus*	Korean Topan Salt	MA	37	7	3	-	-	-	-
1464	*Exiguobacterium mexicanum*	Daebeon port seawater	MA	37	7	3	-	-	-	-
1465	*Bacillus amyloliquefaciens*	Daebeon port seawater	MA	37	7	3	+++	-	-	-
1466	*Staphylococcus equorum*	Jagalchi market seawater	MA	37	7	3	-	-	-	-
1467	*Exiguobacterium mexicanum*	Daebeon port seawater	MA	37	7	3	-	-	-	-
1468	*Halobacillus trueperi*	Korean Topan Salt	MA	37	7	3	-	-	++	-
1469	*Halobacillus halophilus*	Korean Topan Salt	MA	37	7	3	-	-	-	-
1470	*Halobacillus profundi*	volcanic salts	MA	37	7	3	-	-	-	-
1471	*Halobacillus litoralis*	volcanic salts	MA	37	7	3	-	-	-	-
1472	*Halobacillus profundi*	volcanic salts	MA	37	7	3	-	-	-	-
1473	*Chromohalobacter canadensis*	volcanic salts	MA	37	7	3	-	-	-	-
1474	*Staphylococcus warneri*	Gamcheon port seawater	MA	37	7	3	-	-	+++	-
1475	*Bacillus pumilus*	samrak ecological park wetland water	MA	37	7	3	-	-	-	-

1476	*Bacillus pumilus*	samrak ecological park wetland water	MA	37	7	3	-	-	-	-
1477	*Bacillus pumilus*	samrak ecological park wetland water	MA	37	7	3	-	-	+	+
1478	*Kangiella koreensis*	Sinan taepyeong salt field	MA	37	7	3	-	-	+++	+++
1479	*Kangiella koreensis*	Sinan taepyeong salt field	MA	37	7	3	-	-	+++	+++
1480	*Kangiella koreensis*	Sinan taepyeong salt field	MA	37	7	3	-	-	+++	+
1481	*Kangiella koreensis*	Sinan taepyeong salt field	MA	37	7	3	-	-	+++	+
1482	*Kangiella koreensis*	Sinan taepyeong salt field	MA	37	7	3	-	-	+++	+
1483	*Kangiella koreensis*	Sinan taepyeong salt field	MA	37	7	3	-	-	+++	+++
1484	*Kangiella koreensis*	Sinan taepyeong salt field	MA	37	7	3	-	-	+++	+++
1485	*Kangiella koreensis*	Sinan taepyeong salt field	MA	37	7	3	-	-	+++	+
1486	*Kangiella koreensis*	Sinan taepyeong salt field	MA	37	7	3	-	-	+++	+
1487	*Bacillus marisflavi*	Daejeo ecological park wetland water	MA	37	7	3	+++	-	-	+
1488	*Brachybacterium paraconglomeratum*	Daejeo ecological park wetland soil	MA	37	7	3	-	-	-	+
1489	*Fictibacillus phosphorivorans*	Daejeo ecological park wetland water	MA	37	7	3	-	-	+	-
1490	*Staphylococcus cohnii subsp. urealyticus*	Fermented skate	MA	37	7	3	-	-	+	-
1491	*Marinobacter algicola*	Sinan taepyeong salt field	MA	37	7	3	+++	-	-	-
1492	*Marinococcus halotolerans*	volcanic salts	MA	37	7	3	-	-	-	-
1493	*Marinococcus luteus*	volcanic salts	MA	37	7	3	-	-	-	-
1494	*Chromohalobacter canadensis*	volcanic salts	MA	37	7	3	-	-	-	+++
1495	*Staphylococcus equorum*	Pusan baekyangsan bamboo forest soil	MA	37	7	3	-	-	-	-
1496	*Staphylococcus lentus*	Fermented skate	MA	37	7	3	-	-	-	-
1497	*Staphylococcus lentus*	Fermented skate	MA	37	7	3	-	-	-	+
1498	*Moellerella wisconsensis*	Fermented skate	MA	37	7	3	-	-	-	-
1499	*Staphylococcus saprophyticus*	Fermented skate	MA	37	7	3	-	-	-	-
1500	*Staphylococcus saprophyticus*	Fermented skate	MA	37	7	3	-	-	-	-
1501	*Halomonas ventosae*	Sinan taepyeong salt field	MA	37	7	3	+++	-	-	-

Table S2. Characteristics of the strains with excellent extracellular enzyme activities of CP series.

No	Project name	Closest match	Source	Screening condition				Extracellular enzyme activity			
				Media	Temp. (°C)	pH	NaCl % (w/v)	Amylase	CMCase	Lipase	Protease
1	CP-01	*Bacillus megaterium*	Soil near Samcheonpo Fish Market	MA	37	7	3	+++	-	-	+++
2	CP-02	*Bacillus aryabhattai*	Yeosu dolsan park soil	MA	37	7	3	+++	-	+++	+++
3	CP-03	*Bacillus clausii*	Gomso fermented food	MA	37	7	10	++	-	+++	+++
4	CP-04	*Bacillus licheniformis*	Gomso fermented food	MA	37	7	10	+++	-	-	+++
5	CP-05	*Bacillus stratosphericus*	Gomso fermented food	MA	37	7	10	-	-	+++	+++
6	CP-06	*Bacillus licheniformis*	Tongyoung sea	MA	37	7	10	+++	-	-	+++
7	CP-07	*Bacillus subtilis*	Yeosu Yeondeung cheonnamsangyo	MA	37	7	3	+++	-	+++	+++
8	CP-08	*Bacillus firmus*	yellow soil oak pyroligneous liquor	MA	45	7	3	+++	-	-	+++
9	CP-09	*Bacillus cereus*	Kangwon hardwood charcoal	MA	45	7	3	+++	-	-	+++
10	CP-10	*Bacillus flexus*	Munjiang sulphur hot spring	MA	45	7	3	+++	-	-	+++
11	CP-11	*Bacillus hwajinpoensis*	Suncheon bay soil	MA	37	7	3	+++	-	++	+++
12	CP-12	*Bacillus hisashii*	Dongnae bathhouse	MA	45	7	3	++	+++	-	+
13	CP-13	*Bacillus licheniformis*	volcanic salts	MA	45	8	3	+++	-	++	+++
14	CP-14	*Bacillus licheniformis*	volcanic salts	MA	45	8	3	+++	-	++	+++
15	CP-15	*Bacillus cibi*	Dadaepo port seawater	MA	37	7	3	+++	-	-	+++
16	CP-16	*Bacillus cibi*	Daejeo ecological park wetland soil	MA	37	7	3	+++	-	-	+++
17	CP-17	*Halobacillus trueperi*	Daejeo ecological park wetland soil	MA	37	7	3	++	-	-	+++
18	CP-18	*Halobacillus dabanensis*	volcanic salts	MA	37	7	3	+++	-	-	+++
19	CP-19	*Bacillus toyonensis*	Pusan Amnam park area soil	MA	37	7	3	+++	-	-	+++
20	CP-20	*Exiguobacterium mexicanum*	Gamcheon port seawater	MA	37	7	3	+++	-	-	+++
21	CP-21	*Halobacillus trueperi*	Korean Topan salt	MA	37	7	3	+++	-	++	+++
22	CP-22	*Bacillus horikoshii*	Dadaepo port seawater	MA	25	7	3	+++	-	+	+++
23	CP-23	*Bacillus hwajinpoensis*	Dadaepo port seawater	MA	25	7	3	+++	-	+	+++
24	CP-24	*Bacillus ferrariarum*	Wando seawater	MA	25	7	3	++	-	+	+++
25	CP-25	*Bacillus alcalophilus*	Wando seawater	MA	25	7	3	+++	-	+	+++
26	CP-26	*Bacillus licheniformis*	Wando seawater	MA	25	7	3	++	+++	-	-
27	CP-27	*Exiguobacterium oxidotolerans*	Songjeong port seawater	MA	37	7	3	+++	-	-	+++
28	CP-28	*Fictibacillus phosphorivorans*	Samrak ecological park wetland water	MA	37	7	3	++	-	-	+++
29	CP-29	*Halobacillus mangrovi*	Sinan taepyeong salt field	MA	37	7	3	+++	-	+	+++
30	CP-30	*Bacillus megaterium*	Seocheon specialized market creek soil	MA	37	7	3	+++	-	-	+++

31	CP-31	*Bacillus subtilis*	Sinan taepyeong salt field	MA	37	7	3	+++	-	-	+++
32	CP-32	*Exiguobacterium indicum*	Miryang danjangcheon soil	MA	37	7	3	+++	-	+++	+++
33	CP-33	*Exiguobacterium indicum*	Miryang danjangcheon soil	MA	37	7	3	+++	-	+++	+++
34	CP-34	*Bacillus paralicheniformis*	Greenfield ranch soil	MA	50	7	5	+++	-	-	+++
35	CP-35	*Bacillus subtilis*	Greenfield ranch soil	MA	37	7	5	+++	-	+	+++
36	CP-36	*Bacillus lehensis*	Ole market hairtail fermented food	MA	37	7	5	+++	-	-	+++
37	CP-37	*Bacillus subtilis*	Ole market hairtail fermented food	MA	50	7	5	+++	-	-	+++
38	CP-37	*Bacillus weihenstephanensis*	cypress forest soil soil	MA	25	5	3	+++	-	++	+++
39	CP-38	*Bacillus thermoamylovorans*	peatmoss (Silla univ. field)	MA	50	7	3	+++	-	-	+++
40	CP-39	*Bacillus firmus*	Daepo port seawater	MA	37	7	5	+++	-	-	+++
41	CP-40	*Bacillus megaterium*	Yerae port seawater	MA	37	7	5	+++	-	-	+++
42	CP-41	*Bacillus aquimaris*	Sinan taepyeong salts	MA	37	8	15	+++	-	++	+++
43	CP-42	*Bacillus velezensis*	Moslpo port seawater	MA	37	7	10	+++	-	-	+++
44	CP-43	*Bacillus hwajinpoensis*	Daepo port soil	MA	37	7	5	+++	-	-	+++
45	CP-44	*Bacillus hwajinpoensis*	Daepo port soil	MA	37	7	5	+++	-	-	+++
46	CP-45	*Bacillus velezensis*	fermented food (soybean)	MA	25	5	3	+++	-	-	+++
47	CP-46	*Bacillus wiedmannii*	cypress forest soil soil	MA	25	7	3	+++	-	+++	+++
48	CP-47	*Bacillus velezensis*	fermented food (soybean)	MA	25	5	3	+++	-	-	+++
49	CP-48	*Bacillus wiedmannii*	beach seawater	MA	25	9	3	+++	-	+++	+++
50	CP-49	*Bacillus hwajinpoensis*	beach soil	MA	25	9	3	+++	-	+++	+++
51	CP-50	*Bacillus weihenstephanensis*	cypress forest soil soil	MA	25	9	3	+++	-	++	+++
52	CP-51	*Bacillus cereus*	Korean obsevatory soil	MA	25	9	3	+++	-	+++	+++
53	CP-52	*Bacillus amyloliquefaciens*	fermented food (soybean)	MA	25	5	3	+++	-	-	+++
54	CP-53	*Bacillus velezensis*	fermented food (soybean)	MA	25	5	3	+++	-	-	+++
55	CP-54	*Bacillus firmus*	beach soil	MA	37	7	3	++	-	-	+++
56	CP-55	*Exiguobacterium indicum*	Geumil beach crab haegeum water	MA	37	7	3	+++	-	-	+++
57	CP-56	*Bacillus hwajinpoensis*	beach soil	MA	37	9	3	+++	-	+	+++
58	CP-58	*Bacillus amyloliquefaciens*	fermented food (soybean)	MA	25	7	3	+++	-	-	+++
59	CP-59	*Bacillus wiedmannii*	Izhara pension hill soil	MA	25	7	3	+++	-	+++	+++
60	CP-60	*Bacillus hwajinpoensis*	beach soil	MA	25	7	3	+++	-	+++	+++

*CP series strains, strains with multiple extracellular enzyme activities.
